# Deliberation during online bargaining reveals strategic information

**DOI:** 10.1073/pnas.2410956122

**Published:** 2025-02-12

**Authors:** Miruna Cotet, Wenjia Joyce Zhao, Ian Krajbich

**Affiliations:** ^a^Department of Psychology, The Ohio State University, Columbus, OH 43210; ^b^Department of Psychology, University of Warwick, Coventry CV4 7AL, United Kingdom; ^c^Department of Psychology, University of California, Los Angeles, CA 90095; ^d^Department of Economics, The Ohio State University, Columbus, OH 43210

**Keywords:** bargaining, response times, drift-diffusion model, game theory, big data

## Abstract

A standard assumption in game theory is that decision-makers have preplanned strategies telling them what actions to take for every contingency. In contrast, nonstrategic decisions often involve an on-the-spot comparison process, with longer response times (RT) for choices between more similarly appealing options. If strategic decisions also exhibit these patterns, then RT might betray private information and alter game theory predictions. Here, we examined bargaining behavior to determine whether RT reveals private information in strategic settings. Using preexisting and experimental data from eBay, we show that both buyers and sellers take hours longer to accept bad offers and to reject good offers. We find nearly identical patterns in the two datasets, indicating a causal effect of offer size on RT. However, this relationship is half as strong for rejections as for acceptances, reducing the amount of useful private information revealed by the sellers. Counter to our predictions, buyers are discouraged by slow rejections—they are less likely to counteroffer to slow sellers. We also show that a drift-diffusion model (DDM), traditionally limited to decisions on the order of seconds, can account for decisions on the order of hours, sometimes days. The DDM reveals that more experienced sellers are less cautious and more inclined to accept offers. In summary, strategic decisions are inconsistent with preplanned strategies. This underscores the need for game theory to incorporate RT as a strategic variable and broadens the applicability of the DDM to slow decisions.

## Significance

In strategic interactions like bargaining, agents often have private information that they wish to conceal from others. However, if agents make decisions on the spot, then their response times (RT) may inadvertently betray some of their private information. Indeed, we find that bargainers on eBay take hours longer to accept bad offers and reject good offers. Computational modeling reveals that more experienced sellers are hastier with their decisions and more willing to accept offers. Our results challenge the notion that strategic decisions are preplanned and show that RT can be used strategically when negotiating.

When people interact in strategic settings, do they have a plan, or do they make decisions on the spot? Do they respond immediately, or does it take time to figure out what to do? For example, does someone selling their car know what offers they will accept or reject? And does the buyer know what counteroffers they will accept or reject?

**Fig. 1.**
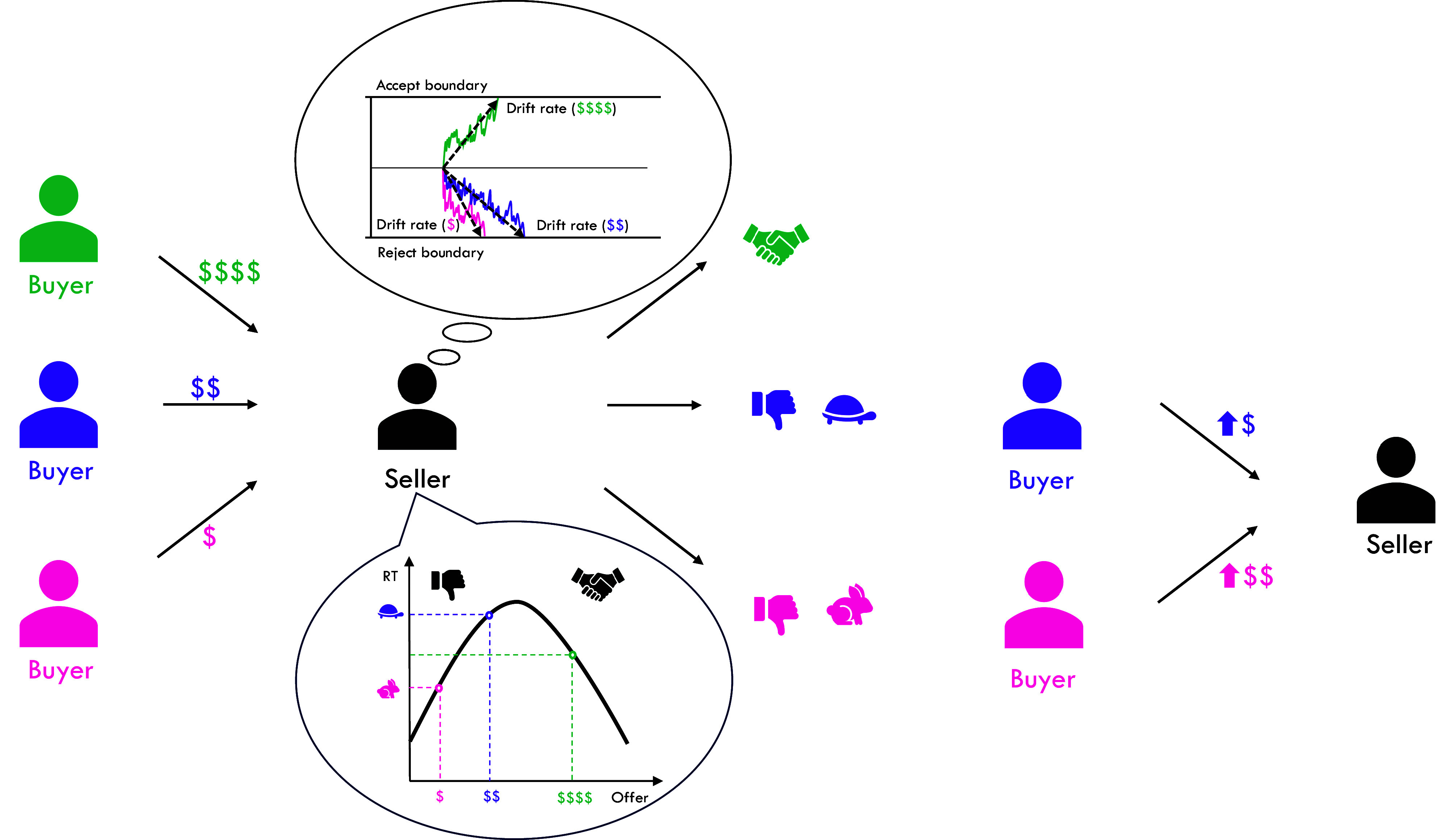
Bargaining setting. In a standard bargaining exchange, the proposer makes an offer to the responder, which the responder can either accept or reject. On eBay, buyers make offers to sellers. The size of the offer, in combination with the seller’s private value, determines the drift rate in the DDM. Here, the high offer (in green) yields a modest positive drift rate (toward the “accept” boundary), the medium offer (in purple) yields a low negative drift rate (toward the “reject” boundary), and a low offer (in pink) yields a high negative drift rate. As a result, the seller accepts the high offer with medium speed, rejects the medium offer slowly, and rejects the low offer quickly. So, a strategic buyer will increase their next offer a lot after a quick rejection, but only a little after a slow rejection.

Whether people have prepared plans is an important question, because without them, people risk revealing private information (1–9). Private information is a central concern in strategic settings—it is information that people have that gives them an advantage over others and that they would like to keep hidden (10). For example, when bargaining over a car, a seller might be willing to accept a small amount but would not want to reveal that to a potential buyer, who might be willing to pay a lot more. With a prepared plan, the seller would be able to instantly accept or reject any offer from the buyer. Without a prepared plan, the seller might require time to consider the buyer’s offer, inadvertently betraying how attractive they find it. A quick rejection could signal a noncompetitive offer while a slow rejection could signal a close call (11, 12). This information could in turn be used by the buyer to make smarter follow-up offers (Fig. 1). The central question in this paper is whether bargainers’ response times (RT) convey such information.

To tackle this question, we need to understand what choice process bargainers might be using, if not executing a prepared plan. Many quick decisions involve a process of accumulating and comparing evidence up to a predetermined boundary, a process which takes time and reflects strength of preference (13–25). The evidence reflects the person’s evaluation of the options—a person deciding between an apple and an orange must weigh the costs and benefits of the apple against those of the orange. If these two evaluations are roughly equal, the person will struggle to decide which item to choose. On the other hand, if the person finds the orange to be much more attractive than the apple, then their choice will be quick and predictable. This relation between strength of preference and RT is a basic feature of evidence-accumulation or sequential-sampling models, such as the drift-diffusion model (DDM). Sequential sampling models like the DDM are typically applied to fast perceptual judgments, but in recent years they have seen increasing application to economic choice (13, 23, 26–45).

Despite the evidence supporting the DDM in economic choice, it is still unclear whether its predictions extend to strategic settings. One recent article by Konovalov and Krajbich (12) (KK) examined the potential ways in which bargaining could play out with a DDM decision process. Theoretically, they found multiple possibilities: Bargainers might always make instantaneous decisions (to mask their private information) or they might still take time to make their decisions. In any case, KK assumed that bargainers are forced to learn their values at the time they receive an offer. Their laboratory experiments reflect this assumption: College undergraduates bargained repeatedly over vouchers with induced values and their decisions were limited to less than 10 s. These results cannot tell us whether the DDM extends to experienced sellers, bargaining over familiar goods, with RT on the order of minutes, hours, or days. In fact, the DDM has not been applied to natural decisions longer than a few seconds (46). Thus, it is still unclear whether RT is informative during bargaining and, if so, whether DDM can account for the patterns in the data.

Here, we examine DDM predictions in bargaining (47–50), using field data and field experiments, with RT ranging from a few seconds to multiple days. Our field setting is eBay—one of the world’s largest online marketplaces. Since 2005, eBay has allowed people to sell their products through an alternating-offer protocol where sellers post items for sale and buyers can make them offers. eBay recently released a dataset with millions of bargaining exchanges from May 2012 to June 2013 (51). We analyzed these data to test whether sellers’ RT reflected the quality of the offers that they received. Because these data can only provide correlational results, we additionally conducted and analyzed a preregistered field experiment on eBay where we acted as buyers, experimentally varying offer size to study sellers’ resulting RT without the potential confounds of nonexperimental data. To preview the results, we find that sellers’ (and buyers’) RT do indeed reflect the quality of the offers they receive. The size of these effects is remarkably similar between the preexisting data and the field-experiment data. Moreover, a DDM, with some adjustments to account for long nondecision times, does a reasonable job of explaining sellers’ choices and RT. We conclude that most sellers do not have prepared plans and instead evaluate offers as they come in. As a result, their RT convey useful information that can be used against them in strategic settings.

## Results

On eBay, a seller can post an item for sale along with an accompanying list price. Buyers can then purchase the item instantly at that price. A seller can additionally enable a “make an offer” option, which allows buyers to initiate a bargaining process. The process begins with the buyer making the seller an offer. The seller can accept, reject, or counter the offer. If countered, the buyer can then accept, reject, or counter that offer. This process continues until a sale is made or until 3 offers are made by each party. There is a deadline of 48 h to respond to each offer. After that time, the offer is automatically rejected. In the data, a single exchange is a collection of offers between a buyer and a seller for a specific item.

We examine two eBay bargaining datasets. The first dataset comes from Backus et al. The second dataset comes from a field experiment that we conducted.

The Backus et al. dataset consists of a year’s worth of bargaining exchanges (between May 2012 and June 2013) on eBay (51). For practical reasons, we only analyze a randomly selected ~20% of that data, resulting in 1.02 million bargaining exchanges.

We excluded all exchanges where an offer arrived from a different buyer. Our model of the seller’s decision is that they compare the attractiveness of the buyer’s offer to the potential benefits of waiting for future offers (or perhaps keeping the item). If another offer actually arrives during this decision process, that changes the calculation, and should lead to the immediate rejection of the worse offer. So, we exclude all such cases (8.6% of all exchanges, ignoring the other restrictions).

We also exclude exchanges with items listed at more than $1000 (6.7% of all exchanges, ignoring the other restrictions), where the RT was less than 10 s (including automatic acceptances and rejections; see below), where the offer expired (22.1% of all exchanges, ignoring the other restrictions), where a message was included (9.8% of all exchanges, ignoring the other restrictions), and where a buyer or seller was outside of the United States (15.8% of all exchanges, ignoring the other restrictions) (see *SI Appendix* for full details).

The second dataset was from a preregistered field experiment that we ran from 2020 to 2023. The goal of the experiment was to address the issue of causality: Does the size of the offer cause the RT, or might there be hidden variables that influence both offers and RT (e.g., unobserved seller characteristics)? Acting as buyers on eBay, we identified a set of sellers and made pseudorandom offers to them. We ran the experiment in two waves, the first with 50 sellers and 11 offers per seller, and the second with 150 sellers and 21 offers per seller. In both cases, we preselected sellers based on their number of items for sale, identified items to bid on, and then made offers between 0.3 and 0.9 of list price for the first wave and 0.1 and 0.8 for the second wave. The items that we bid on were collectible trading cards (e.g., baseball cards, Pokemon cards) valued between ~$10 and 20. In total, we made 3,586 offers (see *Materials and Methods* for additional details).

### Sellers Do Not Have Prepared Plans.

To establish whether eBay sellers have prepared plans we look at two things. First, we examine whether they use tools that allow them to automatically accept or reject certain offers. If sellers are aware of these tools and have stable plans, using such thresholds would save them the effort of having to respond to offers. Still, sellers might not be aware of these tools or might want to adjust their plans over time. Therefore, our main analysis focuses on whether sellers’ RT reflect the size of the offers that they receive. A seller who is making decisions on the spot should react to higher offers with faster acceptances and slower rejections. Conversely, a seller with a plan should show no relation between offer size and RT.

The preexisting data from eBay indicate that most sellers (62%) do not use automatic thresholds. In the subset of data that we analyzed, 17% used only rejection thresholds, 5.4% used only acceptance thresholds, and 15.7% used both thresholds. To examine the effect of seller experience on automatic threshold use, we looked at the number of best-offer listings created by each seller (dating back to 2008), The relation between seller experience and threshold usage was quadratic—both inexperienced and highly experienced sellers used thresholds more than medium-experience sellers (*SI Appendix*, Fig. S1). While these results do not rule out that sellers have plans, the low rate of automatic thresholds suggests that sellers prefer to evaluate offers as they arrive.

**Fig. 2.**
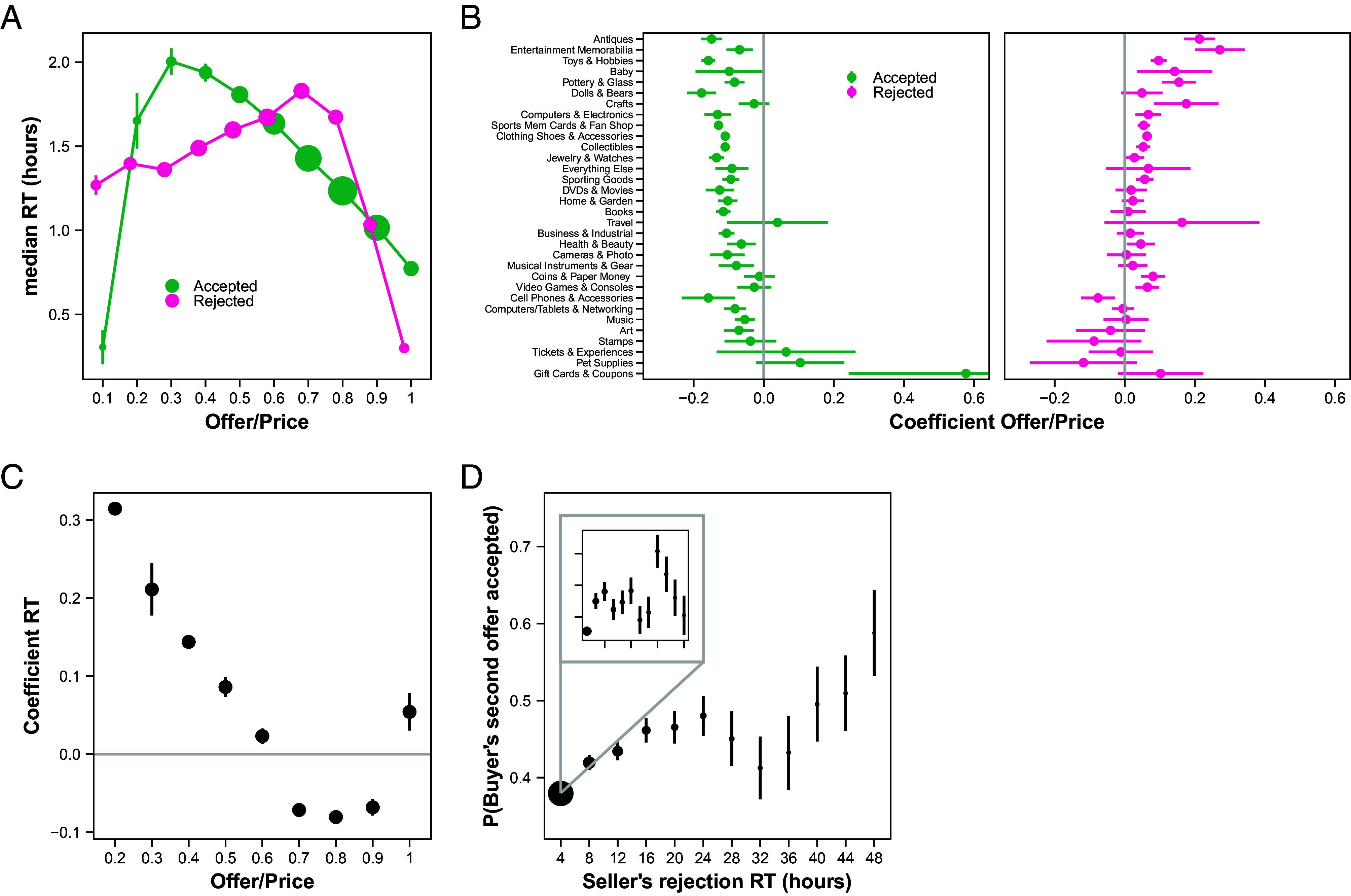
eBay RT reflect offer size. (*A*) Sellers’ median RT (in hours) as a function of buyers’ initial offer ratios (offer/list price), conditional on accepting or rejecting the offers. (*B*) Coefficients for the relation between seller’s log(RT) and buyers’ initial offer ratios, conditional on accepting or rejecting the offers and on item category. (*C*) The effect of sellers’ RT on their probability of accepting the offer, conditional on the offer ratio. Plotted are the logistic regression coefficients for offer ratios in 10% intervals. The regression is the same as in *SI Appendix*, Table S5 (column 3), but with RT (in hours) added as an explanatory variable. These regressions include random effects (clustered by seller) on the intercept. (*D*) The probability of sellers accepting buyers’ first offers as a function of the sellers’ rejection RT to the first offers. In the zoomed-in inset, the probability that the seller accepts the buyer’s second offer ranges from [0.332, 0.418]. For (*A*), the size of the dots indicates the relative amount of data in that bin, across both curves, and the bars represent bootstrapped SE. For (*B* and *C*), the bars represent SE. For (*D*), the size of the dots indicates the relative amount of data in that bin, and the bars represent bootstrapped SE.

Turning to our main analysis, we found that sellers’ RT were highly responsive to the offers they received. We focus on buyers’ initial offers and sellers’ responses to those offers (excluding sellers with automatic thresholds—*Materials and Methods*). Over most of the offer range [20%, 100%], the median acceptance RT decreased with offer size from 2 h down to 0.8 h, while over most of the offer range [0, 70%], the median rejection RT increased with offer size, from 1.3 h up to 1.8 h (Fig. 2*A*). We confirmed these results using linear regressions of log (RT) on offer size (as percent of list price) over the offer range for which both mean acceptance and rejection RT were judged to be monotonic in price (range = [0.36, 0.68]; *Materials and Methods*). Within this range, acceptances had a negative relation between offer size and log(RT) while rejections had a positive relation between offer size and log (RT) (mixed effects with full random effects at the seller level: b_accept_ = –0.24, S.E. = 0.01, 95% CI = [−0.27, −0.22], t(255,930) = −21.77, *P* < 10^−16^ vs. b_reject_ = 0.10, S.E. = 0.01, 95% CI = [0.07, 0.13], t(116,781) = 6.86, *P* = 10^−11^). These results are robust to including various controls, including the offer creation hour, the price of the item, the number of views, the number of watchers, whether the item was relisted, the listing age, the number of photos of the item, buyer experience, seller experience, the number of seller listings, seller feedback, and interactions between the offer and seller experience (*SI Appendix*, Table S2). Seller-level regressions revealed a similar pattern, with 69% showing negative offer-RT correlations for acceptances and 53% showing positive offer-RT correlations for rejections (of 1,127 sellers with at least 50 acceptances or rejections; *SI Appendix*, Fig. S13). These effects were also largely consistent across product categories, ranging from baseball cards to vehicles, with 28/32 categories showing a positive relation between log (RT) and offer size for acceptances and 26/32 categories showing the opposite relation for rejections (Fig. 2*B*). Thus, in line with the DDM, sellers on eBay were slower to reject better offers and to accept worse offers.

Consistent with the DDM, we also found that large “errors” were made very quickly. By errors, we mean low-probability decisions, i.e., rejecting very high offers (> 75%) or accepting very low offers (< 25%) (Fig. 2*A* and *SI Appendix*, Fig. S8 for breakdown by absolute offer level). A counterintuitive prediction of the DDM is that large errors are substantially faster than decisions near indifference (17). Thus, the fast errors that we observed are consistent with a DDM explanation.

Sellers’ counteroffers also displayed a significant relation between log(RT) and offer size. We had speculated that counteroffers might look more like rejections, but instead, they looked more like acceptances. These RT monotonically decreased with offer size over the range [10%, 100%] from 1.46 h to 0.54 h (*SI Appendix*, Fig. S5 and Table S4). Over the same offer range as before ([0.36, 0.68]), we found a significant negative relation between log(RT) and offer size (mixed-effects regression with full random effects at the seller level: b_counter_ = –0.06, S.E. = 0.01, 95% CI = [−0.07, −0.05], t(187,735) = −9.17, *P* < 10^−16^). This result is robust to including the same controls as before. Interestingly, the counteroffers did not display the same fast errors as the acceptances and rejections. Indeed, fast errors are implausible with counteroffers, as sellers have to produce a new offer and not just react to the buyer’s offer.

One explanation for why a seller might delay in responding to an offer is that they are waiting to see if any better offers come in. Because we only consider cases where no other offers were made on the item during the bargaining thread, additional time can only be bad news for the seller. So, an increase in RT should correspond to a higher probability of accepting the offer. Instead, for offers between 60% and 90%, longer RT corresponded to a lower probability of acceptance (Fig. 2*C*). Thus, the idea that sellers are waiting for better offers to arrive is not sufficient to explain their behavior.

An additional prediction that one can derive from the DDM is that sellers who reject an offer more slowly should be more likely to accept a subsequent offer from the buyer, controlling for the size of the offer, because a slower rejection signals a lower value from the seller’s perspective. Indeed, sellers were more likely to accept buyers’ second offers the longer they took to reject the buyers’ first offers (Fig. 2*D* and *SI Appendix*, Table S11; b_rejectionRT_ = 0.13, S.E. = 0.02, 95%CI = [0.10, 0.17], z(23,432) = 7.74, *P* < 10^−15^) controlling for the second offer ratio. This also holds when including additional controls for the item characteristics (number of watchers, views, photos, list price, and listing age) and buyer bargaining history (*SI Appendix*, Table S11; b_rejectionRT_ = 0.12, S.E. = 0.02, 95%CI = [0.08, 0.15], z(23,426) = 6.43, *P* < 10^−9^).

### eBay Field Experiment.

Our eBay field experiment largely confirms the results from the preexisting dataset.

**Fig. 3.**
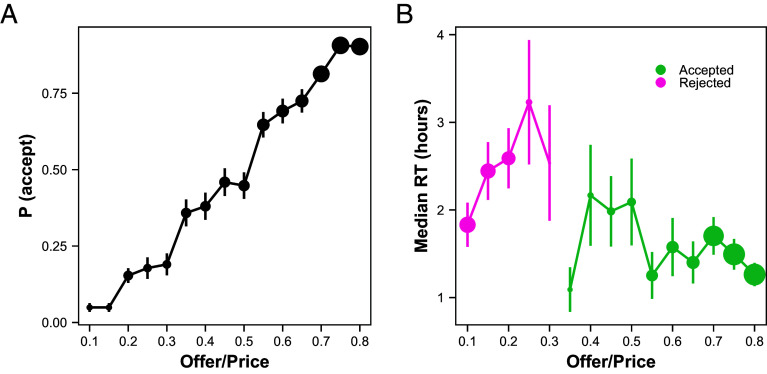
eBay field experiment mirrors preexisting eBay data. (*A*) Sellers’ probability of accepting the first offer as a function of the offer ratio (offer/list price). (*B*) Sellers’ median RT (in hours) as a function of the offer ratio, conditional on acceptance or rejection. The size of the dots indicates the relative amount of data in that bin, across both curves, and the bars represent SE across sellers. Bins with less than 40 observations are excluded.

We made 3,586 offers and had 1,550 acceptances, 644 rejections, and 1,392 counteroffers. Sellers were more likely to accept higher offers (b_Offer/Price_ = 2.64, S.E. = 0.11, 95% CI = [2.43, 2.86], z(2,192) = 24.34, *P* < 10^−16^, Fig. 3*A* and *SI Appendix*, Table S15).

In terms of RT, sellers were significantly faster to accept higher offers, and were nonsignificantly slower to reject higher offers (Fig. 3*B*; the rejection effect was significant, *P* < 0.0001, when including expired offers and assigning them the maximum RT, see *SI Appendix*, Table S22). Regressions of log(RT) on offer ratio revealed nearly identical coefficients to the preexisting eBay data (b_accept_= −0.24, SE = 0.07, 95% CI = [−0.38, −0.10], t(1,544) = −3.37, *P* = 0.001, b_reject_ = 0.11, SE = 0.10, 95% CI = [−0.09, 0.32], t(638) = 1.06, *P* = 0.29, *SI Appendix*, Table S19). Thus, the RT results from our experiment align well with those from the preexisting data.

The RT for counteroffers were one exception. Unlike in the preexisting eBay data, here the counteroffers displayed a positive (though not significant) rather than negative relation between log(RT) and offer ratio (b_counter_ = 0.05, S.E. = 0.08, 95% CI = [−0.11, 0.20], t(938) = 0.57, *P* = 0.569), though the sign was inconsistent between waves of the experiment (*SI Appendix*, Table S20).

### Buyers Do Not Have Prepared Plans Either.

Our primary focus has been on sellers because they have at least one response in every exchange, we have more information on them, and they were the target of our field experiment. However, we can also see whether buyers’ behavior is in line with DDM predictions. When sellers respond with counteroffers, the tables are turned and buyers have to decide whether to accept, reject, or counter. Like with the sellers, the DDM predicts that buyers (without plans) will respond to better counteroffers with faster acceptances and slower rejections.

To quantify the quality of a seller’s counteroffer, we calculated the seller’s “compromise”. We defined the seller’s compromise as the gap between their counteroffer and the list price as a fraction of the gap between the buyer’s offer and the list price. A full compromise (=1) corresponds to the seller counteroffering with the same price that the buyer offered, while no compromise (=0) corresponds to the seller counteroffering with their list price. The bigger the compromise, the more attractive the counteroffer.

**Fig. 4.**
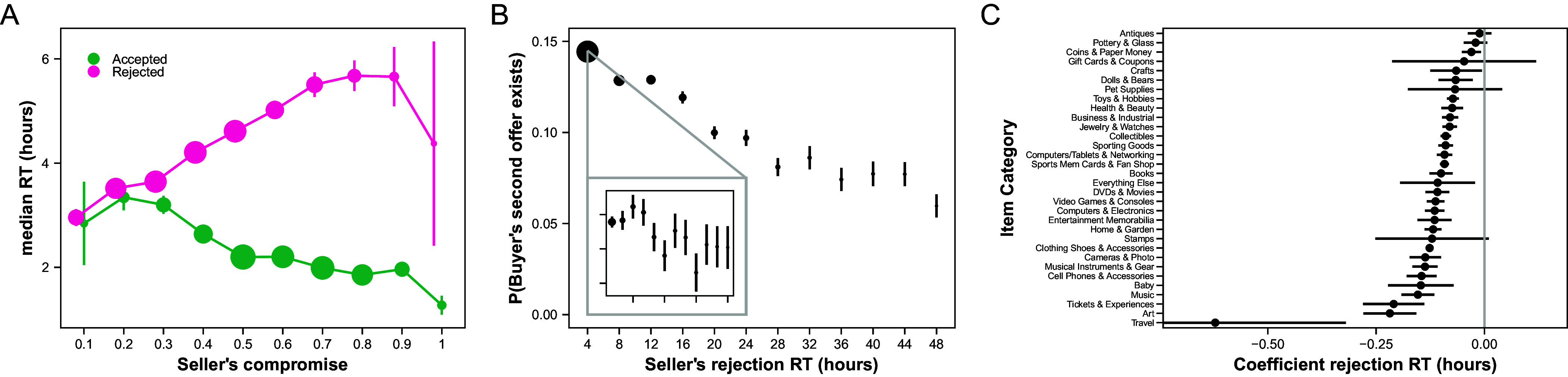
Buyers react adversely to slow sellers. (*A*) Buyers’ median RT (in hours) as a function of sellers’ compromises, conditional on the buyers accepting or rejecting the offers. The seller’s compromise is the amount that they lowered their counteroffer, divided by the gap between the list price and the buyer’s offer. A 100% compromise would be a counteroffer that matches the buyer’s offer; a 0% compromise would be a counteroffer that is the list price. (*B*) The probability that buyers make second offers as a function of sellers’ rejection RT to the first offers. The inset zooms in on rejection between 0 and 2 h. In the zoomed-in inset, the *y*-axis ranges from [0.133, 0.152]. (*C*) Coefficients for the relation between sellers’ rejection RT to the first offers and probability that buyers make second offers, by item category. For (*A*), the size of the dots indicates the relative amount of data in that bin, across both curves, and the bars represent bootstrapped SE. For (*B*), the size of the dots indicates the relative amount of data in that bin, and the bars represent bootstrapped SE. For (*C*), the bars represent SE.

Buyers’ RT did vary with the quality of sellers’ counteroffers as expected (Fig. 4*A* and *SI Appendix*, Table S8). We regressed buyers’ log(RT) on the sellers’ compromise. Larger compromises led to slower rejections and faster acceptances (b_accept_ = −0.13, S.E. = 0.009, 95% CI = [−0.15, −0.11], t(817,789) = −14.39, *P* < 10^−16^, b_reject_ = 0.18, S.E. = 0.008, 95% CI = [0.16, 0.19], t(76,587) = 21.11, *P* < 10^−16^, Fig. 4*A* and *SI Appendix*, Table S8). The difference in rejection RT was substantial, increasing from 2.96 h to 5.58 h for compromises from 10% to 100%. Interestingly, buyers’ RT were consistently longer for rejections than for acceptances. This may be because buyers are on eBay with the intent to purchase, so accepting is, on average, more attractive than rejecting.

Following Konovalov & Krajbich, we next attempted to analyze the size of buyers’ second offers conditional on sellers’ rejection RT. However, this analysis is highly contaminated by selection bias, as buyers made second offers only ~10% of the time, and those who did were surely more determined to acquire the items. In the *SI Appendix*, we report these analyses, as well as an attempt to deal with the selection bias, but given how much data are missing, we refrain from drawing any conclusions from those results.

Instead, we examined whether buyers were more likely to return with follow-up offers after being rejected more slowly. Since a fast rejection from a seller should signal to the buyer that their offer was not competitive, buyers who are rejected quickly should be discouraged from coming back with a second offer. Instead, we observed the opposite result. Using a logistic regression, we regressed whether the buyer made a second offer on the RT of the seller’s rejection. Buyers were more, not less, likely to make a second offer after a faster rejection (Fig. 4*B* and *SI Appendix*, Table S9, b_rejection RT_ = −0.098, S.E. = 0.004, 95% CI = [−0.11, −0.09], z(226,649) = −26.24, *P* < 10^−16^). This result was robust across all the item categories (Fig. 4*C*). Thus, counter to our predictions, buyers on eBay appear to be discouraged by slow rejections.

To understand why buyers are discouraged by slow rejections, we investigated several hypotheses. One possibility is that buyers are impatient and so a slow response from a seller prompts them to move on to other sellers. A second possibility is that impatient buyers choose to simply purchase the item after a slow response in order to avoid further costly delays. A third possibility is that the effect is driven by buyers making offers on impulse goods, i.e., goods that they lose interest in over time. While our data are not ideal for testing these hypotheses, we investigate each one in turn.

The presence of multiple sellers might explain why buyers are encouraged by fast rejections on eBay, opposite to the lab (12). If buyers are impatient and have the option to move on to other sellers, the positive signal from a slow rejection might not be enough to compensate for the buyer’s lost time (52). This hypothesis is difficult to test with our data because we do not know the exact identities of the items. However, we do find that buyers who were rejected more slowly by a seller were less (not more) likely to make another offer to a different seller for an item in the same category within 24 h (b_rejectionRT_ = −0.22, S.E. = 0.02, 95%CI = [−0.25, −0.19], z(226,644) = −13.15, *P* < 10^−15^, *SI Appendix*, Fig. S18), controlling for the first offer ratio, item characteristics (number of watchers, views, photos, list price, listing age), and buyer bargaining experience. Buyers may sometimes move on to other sellers, but this is not more common after slow rejections.

A second possibility is that after a slow response, buyers simply purchase the items to avoid additional costly delays from bargaining. We find a complex relationship between a seller’s rejection RT and the probability of the buyer then purchasing the item. The probability of purchasing decreases with the seller’s rejection RT, up to ~8 h. The probability then rises slightly up to ~24 h, at which point it declines again until bouncing back up at the 48-h time limit (*SI Appendix*, Fig. S19). Thus, for most of the bargaining threads (77% of rejections are under 8 h), these results do not support the idea that buyers are getting impatient and purchasing the items. Instead, the results support our initial hypothesis that fast rejections should be discouraging and so lead buyers to suspend negotiations and instead purchase.

A third possibility is that some buyers might be making impulse offers, and delays in sellers’ responses induce a “cooling-off” period after which buyers’ excitement wears off and they are less likely to return with second offers. To test this hypothesis, we looked to see whether buyers were less likely to return to slow sellers for items deemed to be more impulsive or hedonic. We prompted a Large-Language Model (ChatGPT) to rank our item categories based on impulsiveness, uniqueness, and urgency and correlated these rankings with the effect of the seller’s rejection RT on the probability of the buyer making a second offer. We did not find any significant correlations between the effect of rejection RT on the probability of a second offer and the impulsiveness ranking (Spearman r(29) = −0.18, *P* = 0.315), uniqueness ranking (Spearman r(29) = −0.07, *P* = 0.685), or urgency ranking (Spearman r(29) = 0.29, *P* = 0.103), though for urgency the effect was marginal and in the expected direction. Overall, we do not find support for the effect being driven by cooling down after impulsive offers.

### Modeling eBay Sellers with the DDM.

We have argued that bargainers do not have prepared plans, but does their decision process align with the DDM? To address this question, we adapt a value-based DDM (12) to the bargaining setting. Indeed, this adapted DDM provides an accurate account of sellers’ behavior on eBay.

We model the drift rate in the DDM as a function of the buyer’s first offer (as a fraction of the seller’s list price) and of the seller’s list price. In a typical value-based DDM, the drift rate is a function of the difference in subjective values between the two options. Here, we assume that the list price is a reasonable proxy for the seller’s subjective value for the item. If they accept the offer, they get the buyer’s cash; if they reject the offer, they get the value of the item.

We also developed a more complex formulation for nondecision time. In the DDM, nondecision time is a parameter that accounts for time that the decision-maker is not evaluating or comparing the alternatives. In lab experiments, nondecision time is typically a few hundred milliseconds and modeled with a uniform random variable (17). It is thought to capture motor latencies (i.e., the time to press a button) and initial delays in orienting to the options. In our data, there are many other factors that could contribute to nondecision time. eBay users multitask. Sellers may take hours before seeing an offer and their decision process might be interrupted by sleep, work, family, etc. Thus, we developed models of nondecision time that use Gamma distributions (Model 2), additionally include the time of day (Model 3: sleep and work), and additionally vary with the quality of the offer (Model 4: more interruptions when sellers struggle to decide) (*Materials and Methods*). Each of these models builds on the previous one (*Materials and Methods*). We compared these models using Watanabe-Akaike Information Criterion (WAIC), a standard method for comparing model fit while accounting for model complexity (53).

Relative to the model with the standard nondecision time specification (Model 1), Model 2 improved model fit for 11% of sellers, Model 3 further improved model fit for another 47% of sellers, and Model 4 even further improved model fit for 42% of sellers. Model 1 captured the choice data reasonably well but struggled to account for the RT distributions—it overpredicted the shortest RT and greatly underpredicted the longest RT.

**Fig. 5.**
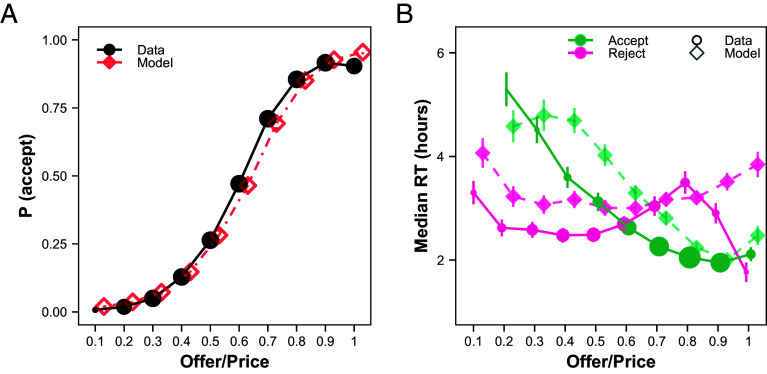
eBay choices and RT are well captured by the DDM. (*A*) Choice data and DDM fits in the preexisting eBay data. Sellers’ probability of accepting the first offer as a function of the offer ratio (offer/list price). (*B*) RT data and model fits in the preexisting eBay data. Sellers’ median RT (in hours) as a function of the offer ratio. The bars represent SE across individuals. The dot sizes represent the amount of data. Bins with less than 15 observations are excluded.

Using the best-fitting nondecision time model for each seller, the DDM accurately captured both choice and RT data from the eBay sellers (at least those that fit our inclusion criteria—*Materials and Methods*) namely the fact that sellers responded to higher offers with a higher probability of acceptance, faster acceptances, and slower rejections (Fig. 5, see *Materials and Methods* for additional posterior predictive checks, *SI Appendix*, Figs. S25–S31).

Using the best-fitting model for each seller, we could capture the aggregate acceptance probability depending on offer ratio or choice difficulty (Fig. 5*A* and *SI Appendix*, Fig. S25) and the aggregate X-shaped RT pattern (Fig. 5*B* and *SI Appendix*, Fig. S26). The model could also account reasonably well for acceptance probability (*SI Appendix*, Fig. S27) and RT quartiles (*SI Appendix*, Fig. S28) across sellers. Moreover, because the models allow nondecision time to vary with the time of offer creation, they could also capture how RT depended on the time the offer was created (*SI Appendix*, Fig. S29). The model and data regression coefficients for offer ratio on log RT (*SI Appendix*, Fig. S31) and for offer ratio on choices (*SI Appendix*, Fig. S30) were also highly correlated.

One question that we can address with the DDM is how sellers change with experience. Here, we measure experience by the number of bargaining exchanges a seller had participated in before the current offer. We find that more experienced eBay sellers exerted less response caution and evaluated offers more positively. When examining RT as a function of offer size for more experienced sellers, we observe a speeding up for acceptances, but not for rejections (b_experience_ = −0.14, S.E. = 0.02, 95% CI = [−0.18, −0.10], t(255,916) = −6.98, *P* = 10^−12^ for acceptances, b_experience_ = −0.013, S.E. = 0.03, 95% CI = [−0.07, 0.04], t(116,767) = −0.45, *P* = 0.66 for rejections, *SI Appendix*, Table S2). Looking at correlations between DDM parameters and experience (*SI Appendix*, Fig. S32), we find that seller experience was negatively correlated with boundary separation (Spearman r(510) = −0.2, *P* = 10^−5^) and positively correlated with the intercept term in the drift-rate function (favoring acceptance) (Spearman r(510) = 0.28, *P* = 10^−10^). Some sellers had a starting point bias toward acceptance or rejection (372 had no starting point bias, 17 had an acceptance starting point bias and 123 had a rejection starting point bias), but this did not correlate with their experience (Spearman r(510) = −0.07, *P* = 0.136).

To incorporate the possibility that sellers might be learning about their items from the market, we fit an additional model in which the drift rate depends not only on the offer ratio and list price but also on the number of watchers, the number of views, and how many days the item had been for sale (Time of Day and Offer Ratio and Item Characteristics Gamma DDM - Model 5). We also fit a similar model where we excluded the offer ratio from the drift regression (Time of Day and Item Characteristics Gamma DDM excluding Offer Ratio from Drift Rate - Model 6) to see whether item characteristics had a larger effect than the offer ratio in determining sellers’ RT.

When comparing all models using WAIC (53), we find that 3% of the sellers were better fit by Model 2, 10% of sellers by Model 3, 3% by Model 4, 84% by Model 5, and none by Model 6. If we only compare Model 4, Model 5, and Model 6, we find that 90% of sellers were better fit by Model 5, while 9% of sellers were better fit by Model 4. No sellers were better fit by Model 6 which excludes the offer ratio from the drift rate.

Model 5 reveals that sellers’ decisions were indeed informed by the market. We found that the number of views and the number of watchers for an item pushed the sellers’ drift rates toward rejection, while the listing age pushed the sellers’ drift rates toward acceptance (95% credible interval for number of views: positive for 0%, negative for 42% of sellers; for number of watchers: positive for 0.6%, negative for 33% of sellers; for listing age in days: positive for 58%, negative for 0.9% of sellers; *SI Appendix*, Fig. S33). Even for the model including item characteristics in the drift rate our previous results still hold (*SI Appendix*, Fig. S34), namely a negative correlation between seller experience and boundary separation (Spearman r(514) = −0.19, *P* < 10^−4^) and a positive correlation between seller experience and drift bias (Spearman r(514) = 0.28, *P* < 10^−10^).

## Discussion

In summary, our results indicate that counter to implicit assumptions in economics and game theory, many people do not have deterministic threshold strategies, but instead make decisions on the spot, in a way that reflects their strength of preference. Buyers and sellers on eBay take hours longer to accept unattractive offers and to reject attractive offers. They may have plans, but those plans involve noisy and lengthy evaluations of the received offers. As a result, their RT reveal private information, namely their evaluation of received offers, which opposing agents can use to gain an advantage in the bargaining process. Sellers’ decisions are well explained by a computational model (DDM) where evidence in favor of accepting accumulates at a rate proportional to the size of the buyers’ offers. The DDM also reveals that sellers with more experience use narrower decision boundaries and evaluate offers more positively. Surprisingly, eBay buyers do not respond to fast rejections as expected—rather than being discouraged, they are more likely to come back with a new offer.

There are a couple of key takeaways from our study. First, RT is an important strategic variable, one that has so far been ignored in game theory and until recently in economics (9, 44, 54–62). Practitioners, experimenters, and theorists alike must consider the consequences of people inadvertently revealing private information through RT. In the labor market, job applicants can observe how quickly they receive a job offer after an interview. A fast job offer may signal that the employer has a strong preference for the candidate. This information can affect the applicant’s decision to accept the offer (63). In political campaigns, the speed of endorsements for a candidate can provide information about the strength of the endorser’s preference. Slow endorsements may indicate weak support, while early endorsements can be more convincing (64). In academia, RT of referees and journal editors are correlated with the quality of the paper (65). RT can also be used in financial markets. It has been shown that the timing of stock analyst recommendations can impact subsequent analysts’ recommendations (66). The speed of trades in asset market transactions can reveal information about the existence of insiders (67, 68). In both online and live auctions, the timing of bids can be informative. Bidding frenzies can be influenced by the frequency of bids and this tends to increase product valuations (69). In negotiations, when proposers’ first offers are immediately accepted, they are more likely to generate counterfactual thoughts about how they could have done better and are therefore less likely to be satisfied with the agreement than are proposers whose offers are not accepted immediately (70). On the flip side, offers accepted after a delay may lead to greater satisfaction for the parties involved (70).

Second, the scope of sequential sampling models like the DDM extends beyond simple, quick decisions. The DDM has been a workhorse in perception (15–17), but its use has been limited to decisions on the order of seconds. However, there is nothing in the DDM that restricts it to that timescale. Our results indicate that the DDM is as relevant to buying a car as it is to choosing what to eat for lunch. Rather than making decisions algorithmically or heuristically, we argue that people typically rely on a noisy evidence accumulation and comparison process that approximates an optimal, but constrained, decision rule (71).

Some might wonder whether bargaining delays reflect sellers waiting for better offers. If this was the case, we would expect a systematic effect where more deliberation time (without any new offers) leads to a higher probability of offer acceptance. In other words, time ticking by with no new offers can only be bad news, pushing the seller to accept the offer they have in hand. Instead, we observe the opposite for high offers—as more time passes, sellers are more likely to reject the offers (Fig. 2*C*). Also, if eBay sellers were waiting for better offers then we would expect many acceptances just before the response deadline. Instead, we find that only 0.13% of acceptances occur in the final hour. The experiment from Konovalov and Krajbich (12) also rules out this explanation in the lab because in that setting there is only one buyer and one seller.

So, what is it that sellers are thinking about during these long deliberation times? The consequences of accepting the offer are straightforward—receiving cash and parting with the item. The consequences of rejecting the offer are not. The seller must estimate the size and timing of future offers, as well as their own evaluation of those offers, and evaluate whether the potential improvement in price is enough to justify the expected delay. The seller must also account for the possibility that they will not get an acceptable offer on their item and will be stuck with it. Indeed, we found that sellers’ decisions are responsive to the market interest in their goods—their evaluation of an offer is negatively affected by the number of views and watchers for the item and positively affected by the number of days the item has gone unsold. It is these considerations (and potentially others) that sellers deliberate over, sometimes extensively.

One interesting finding from our paper is that large errors—accepting very low offers or rejecting very high low offers—are made very quickly. While perhaps surprising, this is actually predicted by the DDM. However, it is possible that there are other reasons for these very quick responses. It could be that these cases are legitimate responses to certain kinds of products, e.g., gift cards or scam products (72). Because we do not have details about the specific items from the preexisting eBay dataset, we can only speculate.

Using the DDM we also found that more experienced sellers exhibit less caution in their choices and are more likely to accept a given offer. More experienced sellers are perhaps less cautious because they are involved in more bargaining threads and so cannot afford to spend as much time on any one offer. We also suspect that they are more likely to accept a given offer because they are less attached to their goods. Indeed, professional sellers are less likely to exhibit the endowment effect (73).

More research is needed to understand the surprising effect that buyers are less likely to return to a seller after a slow rejection. Because a slow rejection signals a competitive offer, buyers should be encouraged and follow up with a slightly higher offer. We failed to find support for several possible explanations for this finding—buyers are impatient and move on to other sellers, buyers are impatient and purchase the item, and buyers cool off after making offers on impulse goods. However, our data are not ideal for testing these hypotheses because we do not know the exact identities of the items being bargained over. An additional possibility is that a buyer may respond more favorably to a quick rejection if they are very uncertain about the value of the item. In that case, a quick rejection could signal a high value to the buyer, which could outweigh the presumably higher price. These hypotheses should be explored in future work.

Another major issue that should be addressed in future work is how bargainers choose to counteroffer rather than accept or reject. Counteroffers are challenging to model in the DDM framework because they involve a continuous decision of how much to counter with. Even ignoring that detail, counteroffers turn the decision into a three-alternative choice problem in which countering is a compromise between the other two options. We are not aware of any sequential sampling models that are appropriate for modeling counteroffers. Instead, as a robustness check, we pooled counteroffers with either acceptances or rejections and verified that our main results still hold (*SI Appendix*, Figs. S25–S31, S35, and S36 and Table S12).

One implication of our results is that equilibrium concepts from game theory may not be well suited to describing behavior. Our results suggest that many agents do not have preplanned strategies and so may not be fully reasoning through their decisions (60, 74). This is in line with research in behavioral game theory (75) such as work on Level-K (76) and Cognitive Hierarchy models (77, 78) where people use limited steps of thinking, Experience Weighted Attraction where people learn strategies based on predispositions and payoff experience (79, 80), and Quantal Response Equilibrium where people are noisy and believe that others are noisy as well (81). Other more recent work has begun to examine strategic decision-making from process-tracing and sequential sampling model perspectives (82–86). Our research builds on that work, using RT to demonstrate that the amount of thinking depends on the difficulty of the decision in extensive-form (i.e., sequential) games.

In conclusion, we have demonstrated that people make strategic decisions, sometimes ones that take hours or days, in a way that is consistent with noisy evidence accumulation and comparison, i.e., sequential sampling or DDM. Rather than having prepared plans, they make decisions on the spot, revealing private information about their preferences. This means that RT is an important strategic variable to be incorporated into game theory and that the range of applications of sequential sampling models like the DDM is greater than previously thought. We hope that future work will continue to push the bounds of how this framework can help us to understand complex, social, and strategic decision-making.

## Materials and Methods

### eBay Observational Data.

The Backus et al. dataset consists of about a year’s worth of bargaining exchanges (between May 1, 2012, and June 1, 2013) on eBay (51). We created a subset of the data by randomly selecting 20% of the sellers and then collecting all of their bargaining exchanges. The dataset contains several useful measures, including unique identifiers for items, buyers, and sellers; seller experience; item characteristics such as list price, sale price, category, list date, number of views, number of watchers, whether the item was relisted, and whether there are acceptance or rejection thresholds; and offer details such as the buyer’s first offer, any subsequent offers, whether any messages were included, and most importantly for us, timestamps on all offers and replies.

Given the nonmonotonicity in the response time (RT) data, it would be inappropriate to model the entire range of offers with linear regressions. Instead, we employed two complementary modeling approaches. First, we modeled RT with generalized additive model (GAM) regressions, which incorporate smooth functions of the independent variables (87) (*SI Appendix*, Fig. S6). Second, we used the GAMs to identify the range of offers for which RT exhibited monotonic trends, based on when the first derivative of each GAM became significantly different from zero beyond their peaks (calculated using finite differences). We then used mixed-effects linear regressions on the resulting monotonic range (*SI Appendix*, Table S2).

### Item Category Features Ranking.

We prompted a Large-Language Model (ChatGPT-4o) to rank our item categories based on impulsiveness, uniqueness, and urgency to test whether delays in the sellers’ responses induce a cooling-off period after which buyers’ excitement wears off and they are less likely to return with second offers. We expected a positive correlation, with higher item category ranking (from 1—most impulsive, most unique, most urgent to 32—least impulsive, least unique, least urgent) and the coefficient for rejection RT predicting buyers’ second-offer probability (higher coefficient corresponding to higher probability that the buyer comes back with a second offer after a slower rejection).

We defined impulsiveness as the “likelihood for a spontaneous purchase and instant gratification potential”. We defined uniqueness as the “distinctiveness or rarity, novelty, custom or limited-edition nature, personal appeal or niche taste”. We defined urgency as the “time-sensitive demand, scarcity, limited-time offer and immediacy of need”.

The prompt we used is as follows:

“Please rank these item categories in terms of impulsiveness, uniqueness and urgency. Provide a ranking for each term. Consider the following for definitions for each term:

Impulsiveness.

Likelihood of a spontaneous purchase: How quickly a buyer might make an unplanned purchase based on emotional appeal or temptation. Emotional appeal means the degree to which an item attracts attention and triggers an immediate “must-have” feeling without much rational consideration. Instant gratification potential: How easily a purchase can provide immediate satisfaction or joy, leading to a quicker decision.

Uniqueness.

Distinctiveness or rarity: How rare or hard-to-find the item is, making it stand out from similar items. Novelty: How fresh or different the item is compared to typical market offerings, creating a sense of originality. Custom or limited-edition nature: The degree to which the item is custom-made, handmade, or part of a limited run, making it more special. Personal appeal: How much the item reflects individual taste or caters to niche interests, which might increase its perceived value.

Urgency.

Time-sensitive demand: How much pressure a buyer might feel to make a quick decision due to factors like limited availability or impending expiration of the offer.

Scarcity: How soon the item may run out of stock or become unavailable, creating a sense of “buy now or miss out.” Limited-time offer: How much the item is tied to a temporary promotion or exclusive deal that pushes the buyer to act quickly. Immediacy of need: The extent to which the item fulfills an immediate, practical need, prompting the buyer to prioritize purchasing it quickly.”

### eBay Field Experiments.

Both experiments were preregistered. The preregistration for eBay field experiment 1 is available at AsPredicted (#44248): https://aspredicted.org/ZXD_DG4. The preregistration for eBay field experiment 2 is available at OSF: https://osf.io/evp2k/?view_only=bc7890e87e7f4a508d1c7af2af22af5e.

### Experiment 1.

#### Accounts and Technical Details.

The study received approval from the Ohio State University Institutional Review Board (2020B0152), which also granted a waiver of consent process. We used three different eBay accounts with different names to make offers to sellers. The accounts had no previous history on eBay. We also created a developer account that used the eBay Application Programming Interface (API) to send requests and return data from the eBay database. We used the eBay APIs to find items, find available information for items and sellers, and retrieve data about the offers we made and their RT. Unfortunately, the eBay API does not directly record the time when the seller responds to an offer, only when the offer is made. Therefore, to measure RT we used the sale timestamp for acceptances, the next offer creation time for counteroffers, and we manually recorded the rejection timestamp from the eBay website.

#### Item Selection Criteria.

We selected items on eBay that had the following characteristics: single baseball cards that were in good, very good, like new, or brand new condition, best offer feature enabled, seller ships from the United States, price between $10 and 20, minimum seller feedback of 99%, free shipping, no expiration within 3 d, and listing not older than 30 d (*SI Appendix*, Table S1). We decided on baseball cards because they are easy to ship and store, and they are frequently sold on eBay.

#### Offer Criteria.

We used a within-subject design in which we targeted specific sellers and made different offers to them on several different items. To do so, we targeted sellers with many items for sale. We selected the first 10,000 items from the baseball card category and selected the first 50 sellers that had more than 15 and less than 100 items for sale. For each seller, we first made an offer at 0.60 of list price. We then replaced 13 sellers that had an automatic acceptance or automatic rejection for this offer.

We then proceeded to make 10 offers to each seller, each for a different posted item, and in a random order. For each seller, we made three offers each at 0.3 and 0.9, and two offers each at 0.45 and 0.75, in addition to the first offer at 0.6 (*SI Appendix*, Fig. S20*A*). If after those 11 offers we had only one rejection for a particular seller, we made one more offer for another item at 0.25 of the list price. If after 11 offers we had only one acceptance for a particular seller, we made one more offer for another item at 0.95 of the list price. If we still had only one acceptance or one rejection for a seller, we excluded that seller and added a new seller. We replaced 6 sellers in this way.

There was a 12-h expiration time on all offers. A seller received only one offer per day and we spaced the offers so that consecutive offers did not overlap. We made approximately 50 offers per day between 9AM and 12 PM (ET) on weekdays. We avoided making offers to sellers who indicated that they were away. We kept making offers to the selected sellers for approximately 2 mo between July and August 2020.

#### Data.

In one instance, we could not retrieve the RT for a rejection so we used the rejection message timestamp instead. In total, we had 549 offers, with 3 autoacceptances, 34 autorejections, 20 expirations, 242 acceptances, 79 rejections, and 171 countered offers (*SI Appendix*, Table S13).

In some cases, we could not retrieve the covariate information. This happened in 11 cases (2%) for seller’s feedback, registration year on eBay, and number of listings available at the start of data collection, and 3 cases for the number of previous offers for an item (0.5%).

### Experiment 2.

#### Accounts and Technical Details.

The study received approval from the Ohio State University Institutional Review Board (2020B0152). We used three different eBay accounts with different names and addresses to make offers to sellers. The accounts had no previous history on eBay. One account was only used to make first offers to the sellers. We used the remaining two accounts to make the rest of the offers. We used the eBay API to find items, find available information for items and sellers, and retrieve data about the offers made and their RT. To measure RT we used the sale timestamp for acceptances, the next offer creation time for counteroffers, and a customized script for rejections. The script accessed our accounts every ~5 min to check whether any offers had changed from pending to rejected. In rare cases, the script failed, in which case we instead used the message timestamps for rejections. These messages were sent by eBay but with some delay. We estimated this delay as being uniformly distributed between 0 and 1 h.

#### Item Selection Criteria.

We selected items on eBay that had the following characteristics: single trading cards, best offer feature enabled, seller ships from the United States, price between $10 and 17 dollars, minimum seller feedback of 99%, and no free shipping. We chose items from the following item categories: sports cards (baseball, soccer, football, basketball, hockey) in excellent, near mint, or very good condition and collectible card games (Magic: the Gathering, Pokemon, Yu-Gi-Oh!) in near mint or better, lightly played (excellent) or moderately played (very good) conditions. We switched to selecting only items that did not offer free shipping so that the seller’s response would be solely based on their value for the item.

#### Offer Criteria.

We again used a within-subject design in which we targeted specific sellers and made different offers to them on several different items. We selected the first 10,000 items from the sports category and the first 10,000 items from the collectible card games category and then selected 150 sellers with more than 21 and less than 500 items. For each seller, we first made an offer at 0.50 of list price. We then replaced 39 sellers that had an automatic acceptance or automatic rejection for this offer. We initially set the item price range to be between $7 and 17 but realized that we could not make our lowest offers for items below $10 since all offers on eBay have to be at least $0.99. Therefore, we later replaced 38 sellers who did not have 20 viable items in this new price range.

We then proceeded to make 20 offers to each of the selected sellers in. We made the following offers to each seller in a random order: two offers at 0.10, 0.15, 0.20, one offer at 0.25, 0.30, 0.35, 0.40, 0.45, 0.60, 0.65, two offers at 0.70, 0.75, 0.80 for a total of 21 offers per seller (including the first offer of 0.5) (*SI Appendix*, Fig. S20*B*). We chose to oversample at the extreme offer levels to make it more likely to see some acceptances for low offers and some rejections for high offers.

There was a 24-h expiration time on all offers. A seller received only one offer per day and we spaced the offers so that consecutive offers did not overlap. We made approximately 100 offers per day between 9AM and 1 PM (ET) on weekdays. We avoided making offers to sellers who indicated that they were away, which led to some delays and incomplete number of offers for some sellers. Some sellers also deleted their listings during the experiment which also led to incomplete number of offers. If a seller made a counteroffer, we always let it expire. We kept making offers to the selected 150 sellers for about 2 mo between February and April 2023.

#### Data.

In a few cases, we could not retrieve the RT. This happened in 18 cases for acceptances (1.3% of acceptances), in 7 cases for rejections (1.2% of rejections), and 6 cases for counteroffers (0.8% of counteroffers). We excluded these observations. In total, we had 3,037 offers, with 11 autoacceptances, 182 autorejections, 200 expired, 1,308 acceptances, 565 rejections, and 771 counteroffers (*SI Appendix*, Table S14).

In some cases, we could not retrieve covariate information. This happened in 21 cases (0.7%) for seller’s feedback, registration year on eBay, and number of listings available at the start of data collection and 54 cases for the number of previous offers for an item (1.8%).

#### Source of RTs for Rejections.

For rejections, 24% of RT come from message notifications, and 76% come from the eBay API. Based on 436 observations, the mean delay in messages timestamps compared to our script timestamp was 26.6 min (Mean = 26.6 min, SD = 17.6 min, Median = 26.7 min). We also ran the regression of log(RT) on offer ratio with a dummy variable for the source of the RT, but the results did not change significantly (*SI Appendix*, Table S21).

### eBay DDM.

#### Data.

We fitted the DDM at the seller level for the observational eBay data used in the behavioral analyses (*SI Appendix*). We imposed the same restrictions on the data as before, except that some restrictions (see restrictions 14 to 22) were imposed only on the first offer, not the entire exchange. Because the model requires many observations, we selected all the sellers from the dataset that had at least 50 rejections and 50 acceptances, leaving us with 534 sellers, 240,620 listings, and 263,215 bargaining threads. We did not consider counteroffers in the modeling.

### DDM Specifications.

The DDM assumes that people integrate evidence in favor of accepting or rejecting an offer over time, with a drift rate (v), specified by a drift intercept (b0) and drift slopes (b1 and b2), which represents the attractiveness of accepting the offer. Decisions begin at the starting point (z) and are made when the accumulated evidence reaches either the acceptance (a) or rejection boundaries (−a). The response time is composed of the amount of time needed for the accumulated evidence to reach a threshold and a nondecision time (t) that represents time that the decision-maker is not evaluating the offer.

We started by fitting a standard DDM (Model 1) to each seller’s choice and RT data. For each seller, we estimated the following parameters: starting point, boundary separation, nondecision time, drift rate intercept, and drift rate slope for ratio of first offer to list price and drift rate slope for list price. Although this model captured the choice data reasonably well (*SI Appendix*, Fig. S27*A*), it had trouble accounting for RT distributions (*SI Appendix*, Fig. S28*A*). Although the correlation between the data and the model was high and significant, the model overpredicted the fastest RTs (25th quantile), slightly underpredicted the median RTs (50th quantile), and greatly underpredicted the slowest RTs (75th quantile). We first sought to improve the model before examining how its parameters relate to bargaining exchange characteristics.

On eBay, sellers might not be regularly checking their accounts, or they might be otherwise occupied (e.g., asleep or at work). Therefore, we considered nondecision times that depend on the time of day when the offer was made. *SI Appendix*, Fig. S12*B* shows a plot of daily activity on eBay. In these data, sellers mostly responded to offers between 7am and 9 pm (PT). There was also a slight bimodality in the activity levels with higher activity during the morning and evenings.

The standard DDM treats nondecision time as a stationary distribution. We next considered a version that treats nondecision time as a gamma distribution (Model 2). The gamma distribution is a waiting time distribution that is characterized by shape and scale parameters (88). If events occur according to a Poisson process with rate λ, then the waiting time until the occurrence of the nth event follows a gamma distribution with parameters (n,1/λ).

[1] $$t∼G\left(\alpha ,\beta \right).$$

We also considered a gamma-distribution model that further allows the shape parameter to depend on the time of day when the offer was created (Model 3). We let the shape parameter be a function of 3 other parameters ($h_{1},h_{2},h_{3}$). We chose this sinusoidal function because it can produce longer nondecision times at night as well as two peaks of activity during the day.

[2] $$t∼G\left(\alpha_{H},\beta \right),$$

[3] $$\alpha_{H}=\text{exp}\left(h_{0}+h_{1}\cos\frac{2\pi H}{24}+h_{2}\;\cos^{2}\frac{2\pi H}{24}\right).$$

We also let nondecision time depend on the difficulty of the decision, namely a quadratic function of the offer ratio (Model 4). If difficult decisions take more time, they are more likely to be interrupted, resulting in additional nondecision time. So, we let the shape parameter of the gamma distribution additionally depend on linear and quadratic effects of offer ratio, and the size of these effects were captured by two additional parmaters


[4] $$\begin{array}{c} (d_{1},d_{2}).\\ t∼G\left(\alpha_{H},\beta \right), \end{array}$$

[5] $$\alpha_{H}=\text{exp}\left(h_{0}+h_{1}\;\cos\frac{2\pi H}{24}+h_{2}\;\cos^{2}\frac{2\pi H}{24}+d_{1}\left(\frac{p_{1}}{p_{2}}\right)+d_{2}{\left(\frac{p_{1}}{p_{2}}\right)}^{2}\right).$$

We use the following weakly informative prior for the parameters.

[6] $$\alpha ∼N\left(2,1\right),a\geq 0,$$

[7] $$z∼N\left(0.5,0.5\right),0\leq z\leq 1,$$

[8] $$\beta_{0}∼N\left(0,3\right),$$

[9] $$\beta_{1}∼N\left(0,3\right),$$

[10] $$\beta_{2}∼N\left(0,3\right),$$

[11] $$h_{0}∼N\left(0,2\right),$$

[12] $$h_{1}∼N\left(0,2\right),$$

[13] $$h_{2}∼N\left(0,2\right),$$

[14] $$\beta ∼N\left(0,2\right),\beta \geq 0,$$

[15] $$d_{1}∼N\left(0,3\right),$$

[16] $$d_{2}∼N\left(0,3\right).$$

For the standard DDM, we used the same priors as above with the following exception:

[17] $$\beta_{0}∼N\left(0,0.3\right),$$

[18] $$\beta_{1}∼N\left(0,0.3\right),$$

[19] $$\beta_{2}∼N\left(0,0.3\right).$$

We used the Ohio Supercomputer Center to fit the data. We ran 3 separate chains for each subject. Each chain consisted of 3,200 samples out of which 700 were warm-up samples. In cases where the models did not converge, we used 10,000 samples out of which 5,000 were warm-up samples. We computed $\hat{R}$ of all parameters to assess model convergence.

There were 22 unique sellers for which the model did not converge (7 for Standard DDM – Model 1, 5 for Gamma DDM – Model 2, 7 for Time of Day Gamma DDM – Model 3, 13 for Time of Day and Offer Ratio Gamma DDM – Model 4). For the following analyses, we excluded these sellers and were left with 512 total sellers. The maximum $\hat{R}$ was less than 1.05 for all other sellers indicating the models converged successfully (89).

For Models 5 and 6, there were 44 unique sellers for whom the models did not converge ($\hat{R}$ > 1.1; 18 sellers for Time of Day and Offer Ratio and Item Characteristics Gamma DDM - Model 5 and 33 sellers for Time of Day and Item Characteristics Gamma DDM excluding Offer Ratio from Drift Rate - Model 6). For the analyses including these models, we excluded these sellers and were left with 489 total sellers.

In order to test the robustness of the results to excluding counteroffers, we also fit Models 1 to 4 first treating counteroffers the same as rejections and second treating counteroffers the same as acceptances.

We used the same fitting procedure as before. For the model fitting when pooling counteroffers with rejections, there were 26 unique sellers for whom the models did not converge (5 for Standard DDM – Model 1, 9 for Gamma DDM – Model 2, 10 for Time of Day Gamma DDM – Model 3, 12 for Time of Day and Offer Ratio Gamma DDM – Model 4). For the analyses using these models, we excluded these sellers and were left with 507 total sellers.

For the model fitting when pooling counteroffers with rejections, there were 69 unique sellers for whom the models did not converge (17 for Standard DDM – Model 1, 31 for Gamma DDM – Model 2, 30 for Time of Day Gamma DDM – Model 3, 34 for Time of Day and Offer Ratio Gamma DDM – Model 4). For the analyses using these models, we excluded these sellers and were left with 463 total sellers.

### DDM Posterior Predictive Checks.

In order to check how well the models fit the data, we generated simulations of choices and RT using the mean parameter values for the best fitting model for each subject. We generated 10 simulations for each trial. Each trial’s drift rate and nondecision time was determined using the trial’s offer creation time, offer ratio, and list price. We used the *RWiener* package to generate RT and choices (90). We also generated simulations for each model separately to compare how well each model fit the data.

We checked the model fit using the following criteria: how well the model matched probability of acceptance across sellers, how well the model matched RT quartiles for acceptances and rejections across sellers, how well the model could predict the aggregate relationship between offer ratio and RT and the aggregate relationship between offer ratio and probability of acceptance, how well the model matched the mean RT for each hour in the day, and how well the model matched the regression coefficients for offer ratio on RT and on choices.

## Supplementary Material

Appendix 01 (PDF)

## Data Availability

Anonymized field experiment data have been deposited in OSF (https://osf.io/y5vaz/?view_only=babb02f1609e437aa4689ac43a5a4771) (91).
