# Supplementary material for: Deliberation during online bargaining reveals strategic information
Source: Proc Natl Acad Sci U S A. 2025 Feb 12;122(7):e2410956122. doi: 10.1073/pnas.2410956122 (PMC11848323; doi:10.1073/pnas.2410956122)
Supplement: Supplementary file 1 — Appendix 01 (PDF) [file pnas.2410956122.sapp.pdf]

PNAS

Supporting Information for
Deliberation During Online Bargaining Reveals Strategic Information

Miruna Cotet, Wenjia Zhao, Ian Krajbich

Ian Krajbich
Email: krajbich@ucla.edu

This PDF file includes:

Supporting text
Figures S1 to S38
Tables S1 to S22
SI References

Supplementary Text

1. eBay observational data

1.1. Data cleaning

We imposed some restrictions on the sample. All restrictions were imposed at the exchange level; even if only one offer did not meet the restrictions, the entire exchange was eliminated. For all the analyses, the following restrictions were applied (unless otherwise noted), along with the percentage of the data excluded with the restriction (ignoring the other restrictions):

Restrictions for errors:

(1) If the item is sold, the sale price is above the listing price. (0.2%)
(2) An offer is above the list price. (1.8%)
(3) Either the buyer or the seller make more than three offers. (0.2%)
(4) For an offer with a reply of "countered", a counteroffer does not exist in the exchange. (0.1%)
(5) After an accepted offer, there is a subsequent offer in the exchange. (0.4%)
(6) There is a change in the list price during the exchange. (0.5%)
(7) There is a change in the list price during the time the item was listed. (29.7%)
(8) Buyer's second offer is below their first offer and the third offer below their second. (2.3%)
(9) Seller's second offer is above their first offer and third above their second. (1.7%)
(10) Seller's offer is lower than buyer's previous offer and buyer's offer is higher than seller's previous offer. (0.2%)
(11) Response time is missing or above 48 hours (0%).

After applying restrictions just for errors, we were left with 2,754,383 listings, 3,872,080 threads, 159,782 sellers and 2,002,866 buyers which included all 3 buyer offers. We have 3,872,037 first buyer offers, 846,625 second buyer offers and 266,661 third buyer offers.

Additional restrictions:

(12) List price is higher than 1000 USD. (6.7%)
(13) The seller has an accept or reject price threshold set. (36%)
(14) There is an automatic rejection. (20.4%)
(15) There is an automatic acceptance. (3.3%)
(16) There is an expired offer. (22.1%)
(17) Response time is above 47 hours and 59 minutes. (18.8%)
(18) Response time is below 10 seconds. (23.4%)
(19) Offer is rejected because the seller received a better offer. (1%)

(20) Offer is rejected because the buyer received a better offer. (1%)

(21) There is a competing offers i.e. another offer arriving before the seller decided on the initial offer. (8.6%)

(22) Message is included with the offer. (9.8%)

(23) Buyer or seller is located outside of the US. (15.8%)

The final dataset of first buyer's offers after applying all the restrictions consisted of 1,128,999 bargaining exchanges spread across 1,043,320 listings with 74,443 sellers and 743,343 buyers. Without counteroffers, we had 63,989 sellers, 602,792 buyers, 820,035 listings and 869,395 bargaining exchanges.

1.2. Results: Effects of item characteristics on RT

Looking at other influences on RT, the more desirable the items, the more cautious sellers become when responding to offers. This is illustrated by the fact that higher number of watchers led to longer RT for both rejections ($\beta_{\text{Watchers}} = 0.01, SE = 0.005, 95\% CI = [0.00, 0.02], t(116, 767) = 2.65, p = .008$) and acceptances ($\beta_{\text{Watchers}} = 0.15, SE = 0.01, 95\% CI = [0.14, 0.17], t(255, 916) = 22.96, p < 10^{-16}$), while higher number of views led to longer RT for rejections ($\beta_{\text{Views}} = 0.01, SE = 0.005, 95\% CI = [0.00, 0.02], t(116, 767) = 2.83, p = .005$). Higher list price led to slower acceptances ($\beta_{\text{Price}} = 0.03, SE = 0.01, 95\% CI = [0.02, 0.04], t(255, 916) = 5.18, p < 10^{-16}$) and faster rejections ($\beta_{\text{Price}} = -0.06, SE = 0.01, 95\% CI = [-0.07, -0.05], t(116, 767) = 10.07, p < 10^{-16}$) (Table S2 (3), (6)).

1.3. Results: RT effects of second and third buyer offers

We found similar effects of offer ratio on RT of the seller for second and third buyers offers, namely a positive relationship for rejections and negative relationship for acceptances for most of the offer ranges (Fig. S10, Tables S6, S7). We only included cases where the previous buyer's offers were rejected, not countered.

1.4. Results: RT effects with thresholds

For the main analyses, we excluded exchanges for which an accept or reject threshold was present. However, if the buyer's first offer is between these thresholds, the seller's RT can still be informative. We therefore analyze these cases as well. For these analyses, we used cases where either an accept or reject threshold was present. We regressed logRT on buyers' initial offers as a percent of list price, conditional on the seller accepting or rejecting the offers. We only used offer ratios larger than 0.36 and smaller than 0.68 similar to the analyses of observations without thresholds. As in the main analyses, we found similar effects of buyers' first offer ratio on sellers' RT, namely a positive relationship for rejections and negative relationship for acceptances (Fig. S11).

1.5. Results: RT effects with expired offers

We find that the effect of offer ratio on rejection RT is still positive when including expired offers with maximum RT ($\beta_{\text{Offer/Price}} = 0.21, SE = 0.01, 95\%CI = [0.19, 0.24], t(183, 068) = 19.64, p < 10^{-15}$) (Table S3 (1)). The maximum RT was 48 hours.

1.6. Buyer's second offer and strategic use of RT

In order to investigate whether there was selection bias when analyzing buyers' second offers, we first looked at the probability of the buyer making a second offer as a function of the first offer (Fig. S16). Buyers were most likely to make a second offer if their first offer was around half of the list price. For other first offers there was a sharp decrease in the likelihood of making a second offer (Fig. S16, Table S9). To account for this selection bias, we used the Heckman correction (*1*) (Table S9-10). The Heckman correction is a two-step statistical approach that corrects for non-randomly selected samples. In the first stage, we modeled the probability of the buyer making a second offer conditional on the first offer being rejected (Table S9 (1), (2)). This model helps identify the factors that influence whether the buyer makes a second offer or the selection process and calculates the inverse Mills ratio. The inverse Mills ratio is a measure of the probability of an observation being included in the sample, given its characteristics and the estimated parameters from the selection equation. It provides a way to quantify the selection bias that arises when the sample is not randomly selected from the population. In the second stage, we correct for self-selection by incorporating the inverse Mills ratio as an additional explanatory variable (Table S10 (1), (2)). This adjusted regression accounts for the selection bias by incorporating the correction factor from the selection equation. We estimated this model using the R package sampleSelection (*2*).

Buyers were more, not less, likely to make a second offer after a faster rejection ($\beta_{\text{Rejection RT}} = -0.098, SE = 0.004, 95\%CI = [-0.11, -0.09], z(226, 649) = -26.24, p < 10^{-16}$) (Fig. 4B, Table S9). Then, using a linear regression, we regressed the size of the buyer's second offer on the RT of the seller's rejection. Conditional on making a second offer, buyers offered less, not more, after faster rejections (($\beta_{\text{Rejection RT}} = 0.0026, SE = 0.0005, 95\%CI = [0.002, 0.004], t(226, 645) = 5.01, p < 10^{-7}$)) (Fig. S17, Table S10). The latter result may be due to a selection bias – only the most interested buyers return after a slow rejection, and they are the ones likely to increase their offers the most. In any case, counter to the lab data and to our predictions, buyers on eBay appear to be encouraged by fast rejections.

2. eBay field experiments

2.1. Procedure: Experiment 1

We also collected additional data for a separate project that is not analyzed here. For the additional data, if the first offer was declined, we made a second offer that varied in terms of how much it increased compared to the first offer. We incremented the first offer in order to achieve one of three levels of the second offer (as a fraction of list price): $\{0.5, 0.7, 0.9\}$. These second offers were determined ahead of time. Each first offer had a uniform distribution over the higher second offers. For example, first offers of 0.6 had 50% second offers of 0.7 and 50% second offers of 0.9. First offers of 0.75 and 0.9 had no second offers.

2.2. Procedure: Experiment 2

In order to determine the appropriate sample size we performed a power analysis using the experiment 1 data. We performed the power analysis for acceptances and rejections separately. We selected sellers randomly and for each seller we selected offers randomly with replacement. We ran a mixed effects regression of log RT on offer ratio, with random intercepts at the seller level. We performed this 1000 times for each number of sellers and each number of offers per seller for acceptances and rejections and counted the number of times the coefficient on offer ratio was significant at 5% level in the expected direction. In order to reach a power level of 0.80 we needed at least 100 sellers with 10 acceptances and 5 rejections per seller. We therefore decided to collect 150 sellers and 21 offers for each seller given that we expected that some offers will be automatically accepted, countered or expire, and we might lose some sellers due to insufficient items or delays in making offers over the course of the experiment.

2.3. Results: Experiment 1

2.3.1. Summary statistics

The higher the offer was, the more likely it is to be accepted ($\beta_{\text{Offer/Price}} = 3.55, SE = 0.44, 95\% CI = [2.79, 4.54], z(319) = 8.07, p = 10^{-15}$) (Table S15 (1)). Similar to the eBay observational data, low offers were more likely to be declined while mid-range offers were more likely to be countered (Fig. S21A).

2.3.2. Hypothesis 1: main result

To test the hypothesis, a mixed-effects linear regression was performed with the log of seller's RT as the dependent variable. As independent variables the seller's response, dummy coded as 0 for acceptances and 1 for rejections, and the first offer as a fraction of list price were used, as well as their interaction with the type of response (accept or reject). Full seller random effects were included. In cases where the models did not converge or had singular boundary issues, a model comparison was performed between models with simpler random effects structure and the best model according to the AIC criterion was chosen. A significantly negative coefficient on first offer ratio

was expected, indicating that higher offers were accepted more quickly. A significantly positive coefficient on the interaction between first offer ratio and reject was expected, indicating that for rejections, higher offers were responded to relatively more slowly than acceptances.

We did not find a significant effect of offer ratio on the seller's RT for acceptances ($\beta_{\text{Offer/Price}} = -0.20, SE = 0.21, 95\%CI = [-0.61, 0.22], t(315) = -0.94, p = .348$). However, we found a significantly positive coefficient on the interaction between offer ratio and rejection ($\beta_{\text{Offer/Price:Rejected}} = 1.11, SE = 0.50, 95\%CI = [0.12, 2.10], t(315) = 2.20, p = .029$), indicating that for rejections, higher offers were responded to relatively more slowly compared to acceptances (Table S16 (1)). This was not the case for counteroffers ($\beta_{\text{Offer/Price}} = -0.06, SE = 0.19, 95\%CI = [-0.42, 0.31], t(167) = -0.30, p = .766$) (Table S20 (1)). We also found a significant main effect for rejections on RTs, with rejection decisions being overall slower than acceptance decisions ($\beta_{\text{Rejected}} = 1.34, SE = 0.60, 95\%CI = [0.16, 2.52], t(315) = 2.23, p = .027$) (Table S16 (1)).

In addition to the interaction model, we also wanted to test whether rejections of higher offers are made more slowly; the previous model only evaluates rejection speed relative to acceptance speed. To do so we ran the following two mixed-effects linear regressions, analyzing accepted and rejected offers separately. Although the coefficient on offer ratio on acceptance RT is negative, it is not significant ($\beta_{\text{Offer/Price}} = -0.19, SE = 0.21, 95\%CI = [-0.61, 0.24], t(238) = -0.87, p = .384$). However, we found a marginally significant positive coefficient on ratio offer for the rejections ($\beta_{\text{Offer/Price}} = 0.84, SE = 0.45, 95\%CI = [-0.06, 1.74], t(75) = 1.87, p = .066$) (Table S17 (1), (4)).

2.3.3. Hypotheses 2 and 3: seller experience and item characteristics

We added to the models for acceptance and rejections logRT the following covariates for item characteristics: number of best offers the item received, whether the item was relisted. For this experiment, after 30 days the item is automatically relisted by eBay. The seller can also chose to relist the item manually. We didn't find any significant effects of item characteristics on RTs (Table S17 (2), (3), (5), (6)).

We also added to the models for acceptance and rejections logRT the following covariates for seller characteristics: number of feedbacks received, number of viable items considered for the experiment, years since registration. However, these covariates didn't have an effect on acceptance or rejection RTs (Table S17 (2), (3), (5), (6)).

2.4. Results: Experiment 2

2.4.1. Summary statistics

The higher the offer was, the more likely it is to be accepted ($\beta_{\text{Offer/Price}} = 2.63, SE = 0.12, 95\%CI = [2.41, 2.87], z(1,871) = 22.70, p < 10^{-16}$) (Table S15 (4)). As in the eBay observational data, low offers

were more likely to be declined while mid-range offers were more likely to be countered (Fig. S21B). There was also a tendency to let very low offers expire (Fig. S21B). Even though we try to exclude sellers that had automatic thresholds set, we still encountered cases where our offers were automatically declined. As expected, this was more likely to happen for very low offers (Fig. S21B).

2.4.2. Hypothesis 1: main result

Similar to what we observed in the eBay observational data, we expected that sellers' acceptance times would decrease with increasing offer size and sellers' rejection time would increase with increasing offer size. We did not have a clear hypothesis about counteroffers but would naively expected them to look similar to rejections.

To test the hypothesis, a mixed-effects linear regression was performed with the log of seller's RT as the dependent variable. As independent variables the seller's response, dummy coded as 0 for acceptances and 1 for rejections, and the first offer as a fraction of list price were used, as well as their interaction with the type of response (accept or reject). Full seller random effects were included. In cases where the models did not converge or had singular boundary issues, we performed a model comparison between models with simpler random effects structure and chose the best model according to the AIC criterion. As expected, we found a significantly negative coefficient on offer ratio for acceptance ($\beta_{\text{Offer/Price}} = -0.25, SE = 0.07, 95\%CI = [-0.39, -0.12], t(1, 862) = -3.77, p = .0003$), indicating that higher offers are accepted more quickly. We also found an expected and significantly positive coefficient on the interaction between offer ratio and rejection ($\beta_{\text{Offer/Price:Rejected}} = 0.28, SE = 0.14, 95\%CI = [0.02, 0.55], t(1, 862) = 2.09, p = .039$), indicating that for rejections, higher offers are responded to relatively more slowly compared to acceptances (Table S16 (2)). There was also a positive main effect of rejections on RT ($\beta_{\text{Rejected}} = 0.76, SE = 0.17, 95\%CI = [0.43, 1.09], t(1, 862) = 4.50, p = 10^{-4}$), indicating that rejections were slower than acceptances.

In addition to the interaction model, we also wanted to test whether rejections of higher offers are made more slowly; the previous model only evaluates rejection speed relative to acceptance speed. To do so we ran two mixed-effects linear regressions, analyzing accepted and rejected offers separately. We found the expected negative coefficient on offer ratio for the acceptances ($\beta_{\text{Offer/Price}} = -0.26, SE = 0.07, 95\%CI = [-0.40, -0.12], t(1, 302) = -3.59, p = .0006$), and a positive, but not significant coefficient on offer ratio for the rejections ($\beta_{\text{Offer/Price}} = 0.12, SE = 0.11, 95\%CI = [-0.09, 0.33], t(559) = 1.11, p = .278$) (Table S18 (1), (4)). We did not find a significant coefficient for countered offers on offer ratio ($\beta_{\text{Offer/Price}} = 0.07, SE = 0.09, 95\%CI = [-0.11, 0.24], t(767) = 0.76, p = .448$) (Table S20 (4)).

2.4.3. Hypotheses 2 and 3: seller experience and item characteristics

We expected that sellers with less desirable items such as items with older listing dates would be faster to accept

offers and slower to reject offers. To test the effect of item characteristics on RT, we added measures of item characteristics to the previous model, including number of previous offers for the item, whether the item was relisted. We did not find any significant effects of item characteristics on RTs (Table S18 (2), (3), (5), (6)).

We also expected seller experience to decrease both acceptance and rejection RT. In addition, we expected that higher seller experience would make their rejection times less responsive to offer ratio. To test the effect of seller experience on RT, we added measures of seller experience to the previous model, such as seller's number of feedback rating received, seller's registration year on eBay and number of listings available at the start of data collection.

Only one of the measures of seller experience was significant and only on acceptance RT: years since registration. Contrary to expectations, the main effect of years of experience increased acceptance RT ($\beta_{\text{Years}} = 0.31, SE = 0.14, 95\%CI = [0.05, 0.58], t(1, 272) = 2.30, p = .023$) and the interaction effect was significantly negative ($\beta_{\text{Years:Offer/Price}} = -0.18, SE = 0.08, 95\%CI = [-0.34, -0.03], t(1, 272) = -2.31, p = .023$) (Table S18 (3)).

3. eBay DDM

3.1. Parameter Recovery

We performed parameter recovery to test whether our model parameters could be successfully recovered. We generated simulated datasets and then fit the models to the simulated datasets. The range of parameters values used for each model was based on the parameter values for the fitted models on the data. For each parameter, we used a parameter range that was within 5th and 95th quantile of the fitted parameter values. We used Latin square cube sampling to generate parameter combinations for each model. We performed sampling once, generating 200 parameter combinations for each model. For each parameter combination, we selected a random seller's data to simulate a dataset using the parameter combination. The seller was selected randomly from all sellers whose calculated drift rates were within the 99th and 1st quantiles of all the seller's drift rates distribution. We did this in order to avoid cases with too low or too high drift rate that could cause issues for the RWiener function (*90*). We then fit the models to the simulated datasets.

We used the Ohio Supercomputer Center to fit the data. We ran 3 separate chains for each subject. Each chain consisted of 10,000 samples out of which 5,000 were warm-up samples. We computed $\hat{R}$ of all parameters to assess model convergence.

We had 17 parameter combination for which a model did not converge (none for Standard DDM, 6 for Gamma DDM, 3 for Time of Day Gamma DDM, 8 for Time of Day and Offer Ratio Gamma DDM). The maximum $\hat{R}$ was less than 1.05 for all other subjects indicating the models converged successfully (*89*). We plot the correlation between generated and recovered parameter values for each model. The results are summarized in Figs. S37-38, which show that parameters can be successfully recovered.

3.2. Results: DDM with item characteristics

When looking at the mean posterior values for the item-characteristic parameters of the drift rate of Model 5 we find that higher number of views and higher number of watchers make the seller more likely to reject the offer while higher number of days since the listing was posted make the seller more likely to accept the offer (Fig. S33).

Number of views: The 95% credible interval is above 0 for 0 sellers, below 0 for 215 sellers (42%), and overlaps 0 for 301 sellers (58%). Number of watchers: The 95% credible interval is above 0 for 3 sellers (0.6%), below 0 for 173 sellers (33%), and overlaps 0 for 340 sellers (66%). Listing age (days): The 95% credible interval is above 0 for 300 sellers (58%), below 0 for 5 sellers (0.9%), and overlaps 0 for 211 sellers (41%).

Even for the model including item characteristics in the drift rate our previous results still hold, namely a negative correlation between seller experience and boundary separation (Spearman $r(514) = -.19, p < 10^{-4}$) and a positive

correlation between seller experience and drift bias (Spearman $r(514) = .28, p < 10^{-10}$) (Fig. S34).

3.3. Results: Pooling DDM

3.3.1. Pooling Counteroffers with Rejections

We pooled counteroffers with rejections and refitted all the models in order to show that our conclusions do not change.

7% of sellers were best fit by Model 2, 42% of sellers were best fit by Model 3 and 50% of sellers were best fit by Model 4.

Using the best-fitting non-decision-time model for each seller, the DDM still captured both choice and RT data from the eBay sellers, namely the fact that sellers responded to higher offers with a higher probability of acceptance, faster acceptances, and slower rejections (Fig. S25 E, Fig. S26 E). The model overestimates the RT for acceptances compared to the data and slightly overestimates the RT for rejections compared to the data.

We find that more experienced eBay sellers exerted less response caution and evaluated offers more positively. Looking at correlations between DDM parameters and experience (Fig. S35), we find that seller experience as measured by the number of previous best offer exchanges the seller had participated in was negatively correlated with boundary separation (Spearman $r(505) = -0.18, p = 10^{-4}$). Although seller experience as measured by number of previous best offer exchanges seller had participated in was not significantly positively correlated with the intercept term in the drift-rate function (favoring acceptance) (Spearman $r(505) = 0.28, p = .639$), other measures of seller experience were positively correlated with the intercept in the drift-rate function (Number of listings created dating back to 2008: Spearman $r(505) = 0.11p = 0.014$; Number of previous feedbacks received: Spearman $r(505) = 0.15, p = 10^{-3}$).

Using the best fitting model for each subject, we could capture the aggregate acceptance probability depending on offer ratio or choice difficulty (Fig. S25 E) and the aggregate X- shaped RT pattern (Fig. S26 E). The model could also account reasonably well for acceptance probability (Fig. S27 E) and RT quartiles (Fig. S28 E) across subjects. Moreover, because the models allow non-decision time to vary with the time of offer creation, they could also capture the mean RT depending on the time the offer was created (Fig. S29 E). The model and data regression coefficients for offer ratio on log RT (Fig. S31 E) and for offer ratio on choices (Fig. S30 E) were also highly correlated.

3.3.2. Pooling Counteroffers with Acceptances

We also pooled counteroffers with acceptances and refitted all the models in order to show that our conclusions do not change.

8% of sellers were best fit by Model 2, 46% of sellers were best fit by Model 3 and 46% of sellers were best fit by Model 4.

Using the best-fitting non-decision-time model for each seller, the DDM still captured both choice and RT data from the eBay sellers, namely the fact that sellers responded to higher offers with a higher probability of acceptance, faster acceptances, and slower rejections (Fig. S25 F, Fig. S26 F). The model slightly overestimates the RT for both acceptances and rejections compared to the data.

Similar to our original results, we find that more experienced eBay sellers exerted less response caution and evaluated offers more positively. Looking at correlations between DDM parameters and experience (Fig. S36), we find that seller experience as measured by the number of previous best offer exchanges the seller had participated in was negatively correlated with boundary separation (Spearman $r(461) = -0.18, p = 10^{-4}$) and was positively correlated with the intercept term in the drift rate function (favoring acceptances) (Spearman $r(461) = 0.29, p = 10^{-9}$).

Using the best fitting model for each subject, we could capture the aggregate acceptance probability depending on offer ratio or choice difficulty (Fig. S25 F) and the aggregate X- shaped RT pattern (Fig. S26 F). The model could also account reasonably well for acceptance probability (Fig. 27 F) and RT quartiles (Fig. S28 F) across subjects. Moreover, because the models allow non-decision time to vary with the time of offer creation, they could also capture the mean RT depending on the time the offer was created (Fig. S29 F). The model and data regression coefficients for offer ratio on log RT (Fig. S31 F) and for offer ratio on choices (Fig. S30 F) were also highly correlated.

Supplementary Figures

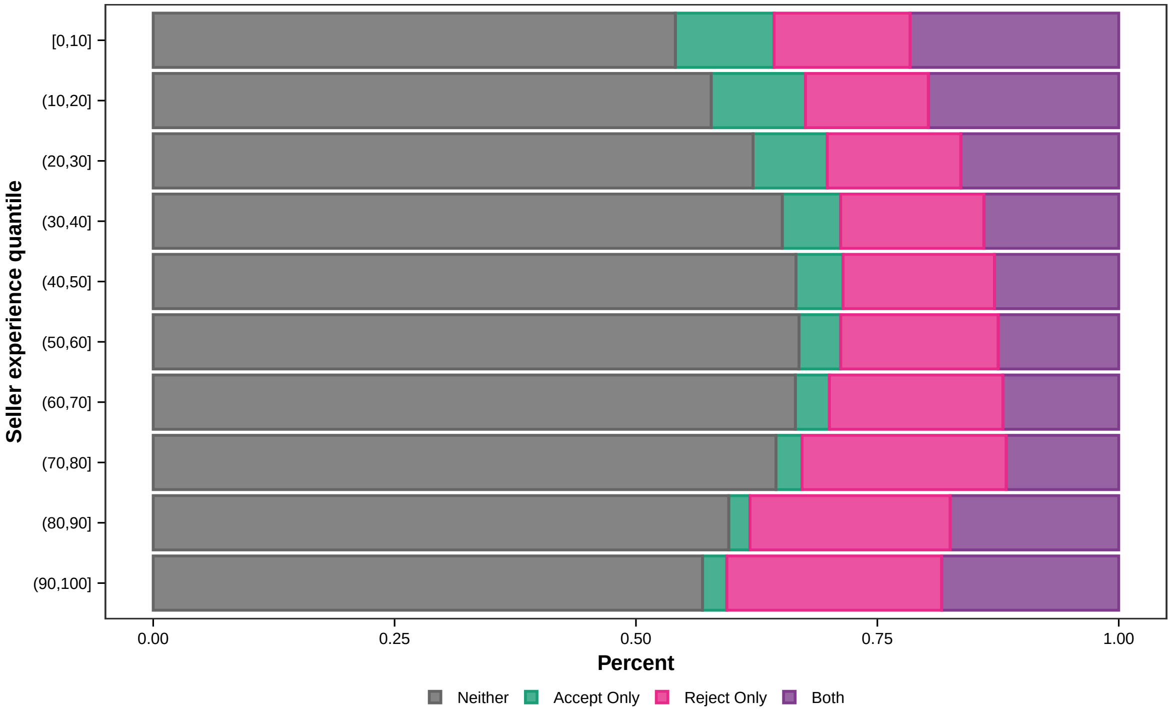

Figure S1. Acceptance and rejection threshold usage by seller experience for eBay observational data. Probability of using an accept or reject threshold, both thresholds or neither threshold, as a function of seller experience quantile. Seller experience represents the number of previous bargaining exchanges the seller has participated in. All bargaining exchanges were sorted by seller experience into ten equal sized bins. In other words, each observation was a single exchange. We did it this way, rather than sorting at the seller level, because a single seller might have some exchanges with thresholds and others without.

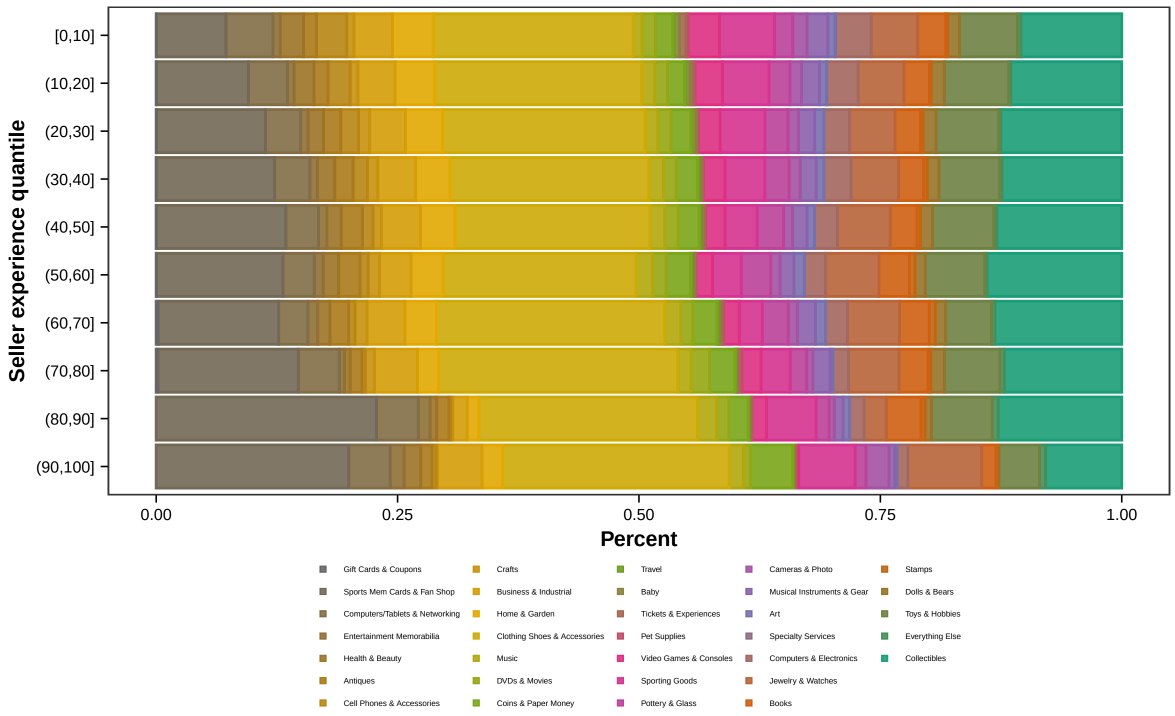

Figure S2. Item category usage by seller experience for eBay observational data. Proportion of each item category sold as a function of seller experience quantile. Seller experience represents the number of previous bargaining exchanges the seller has participated in. All bargaining exchanges were sorted by seller experience into ten equal sized bins; each observation was a single exchange.

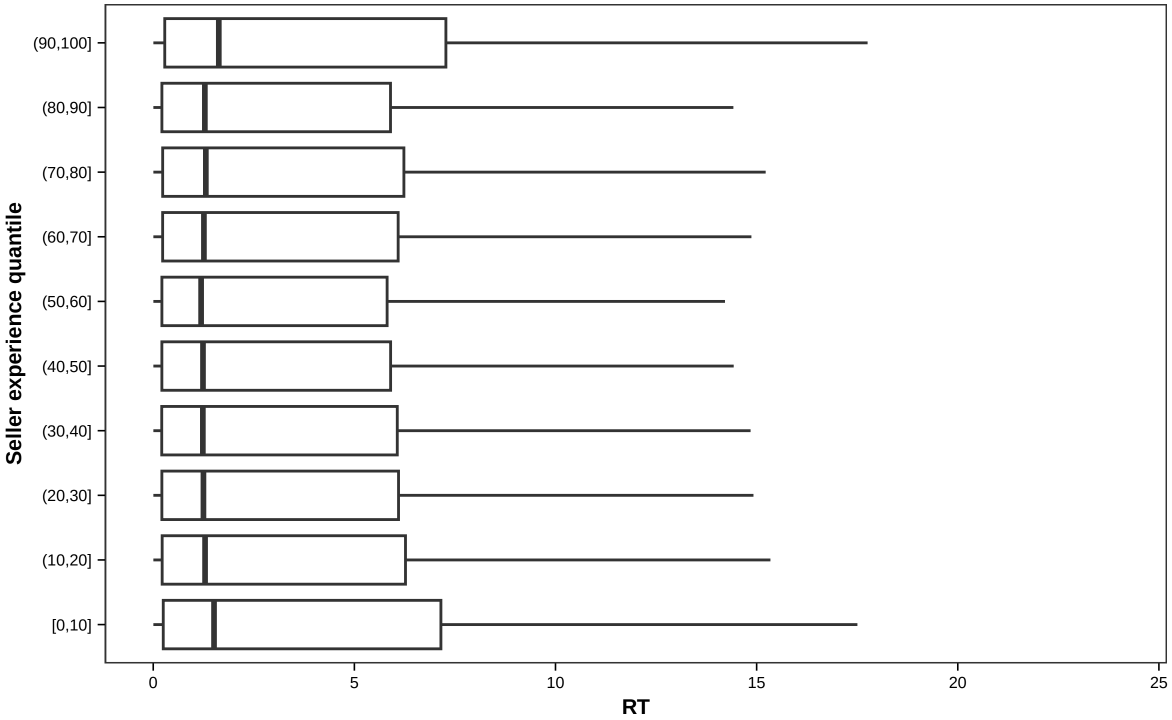

Figure S3. RT by seller experience for eBay observational data. RT boxplot as a function of seller experience quantile. Seller experience represents the number of previous bargaining exchanges the seller has participated in. All bargaining exchanges were sorted by seller experience into ten equal sized bins; each observation was a single exchange.

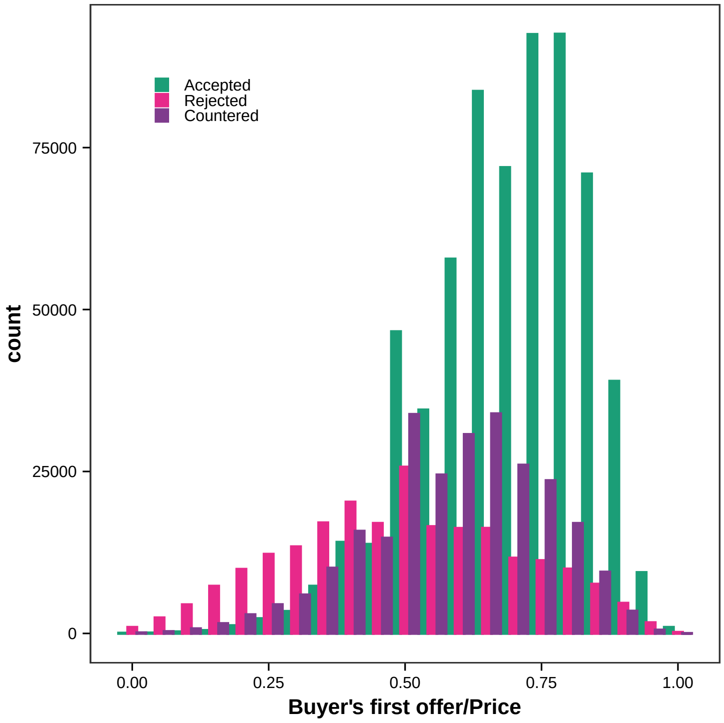

Figure S4. Distribution of buyers' first offer conditional on sellers' response for eBay observational data. Histograms of buyers' initial offers as a fraction of the sellers' list prices, conditional on the seller accepting, rejecting, or countering the offers.

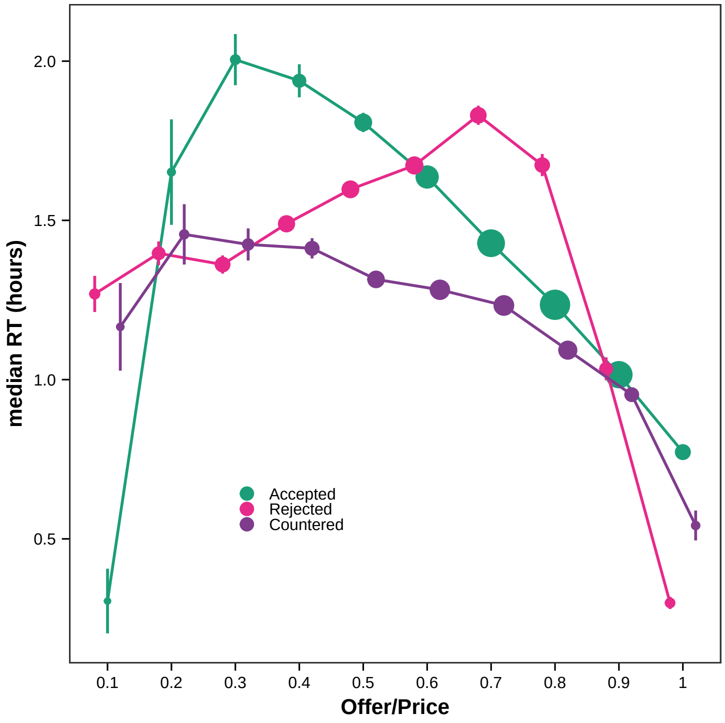

Figure S5. Sellers' median RT for buyers' initial offers by sellers' response for eBay observational data. Sellers' median RT (in hours) as a function of buyers' initial offers as a fraction of the sellers' list prices, conditional on the seller accepting or rejecting or countering the offers. The size of the dots indicates the relative amount of data in that bin, across both curves, and the bars represent bootstrapped standard errors.

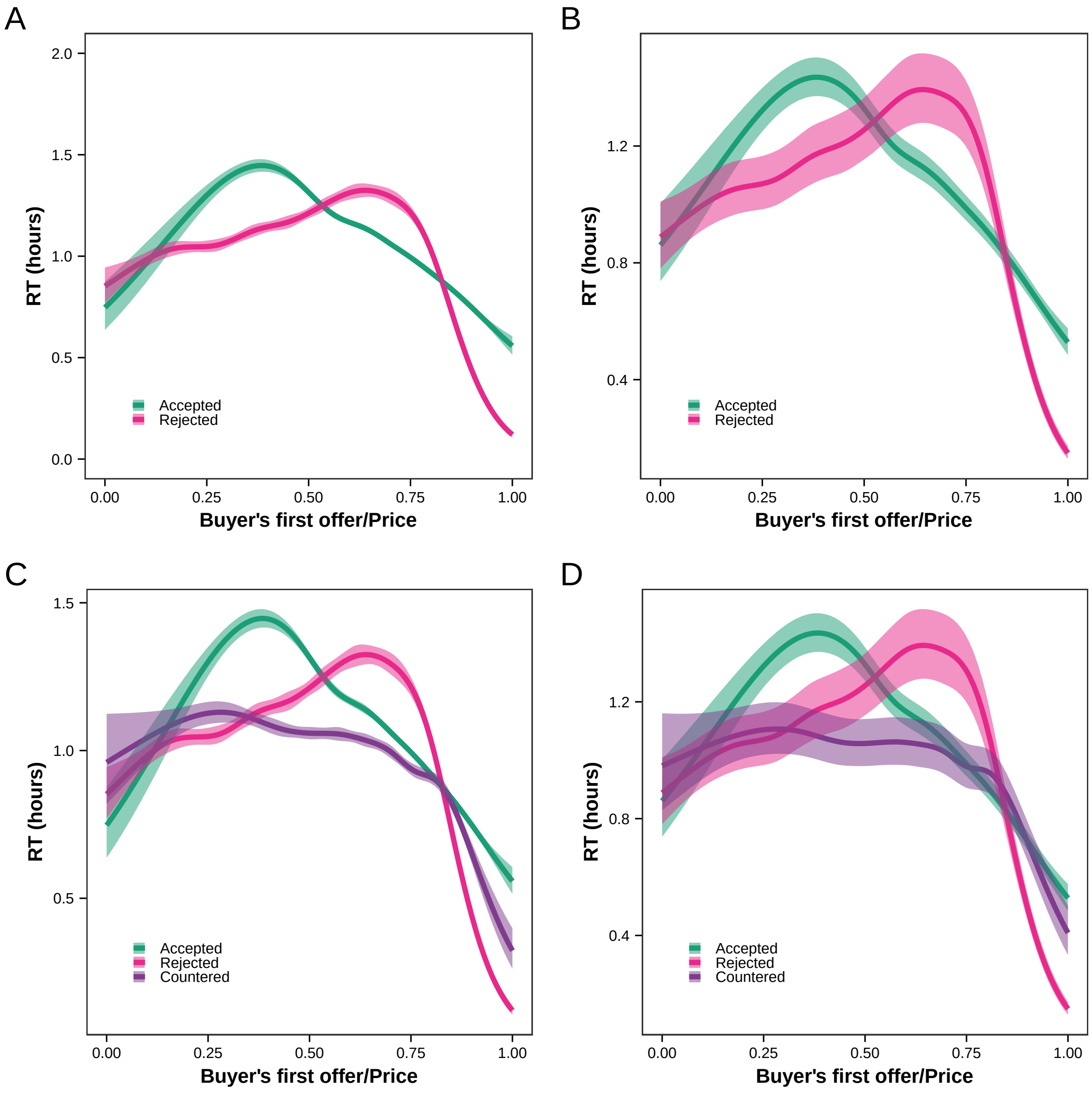

Figure S6. GAM regression predictions for sellers' RT by buyers' initial offer by type of seller response for eBay observational data. (A) GAM regression model fits without covariates. (B) GAM regression model fits with covariates. (C) GAM regression model fits with counteroffers without covariates. (D) GAM regression model fits with counteroffers with covariates. Shaded regions represent 95% confidence intervals. Covariates include item characteristics (list price, number of watchers, number of views, photo count, the list age in days, and whether the item was relisted) or not and buyer and seller characteristics (number of previous best offer exchanges buyer has participated in, number of previous best offer exchanges seller has participated in, seller's number of previous feedbacks received at the time of the offer, number of listings created by the seller dating back to 2008, number of

Best Offer-listings created by the seller dating back to 2008).

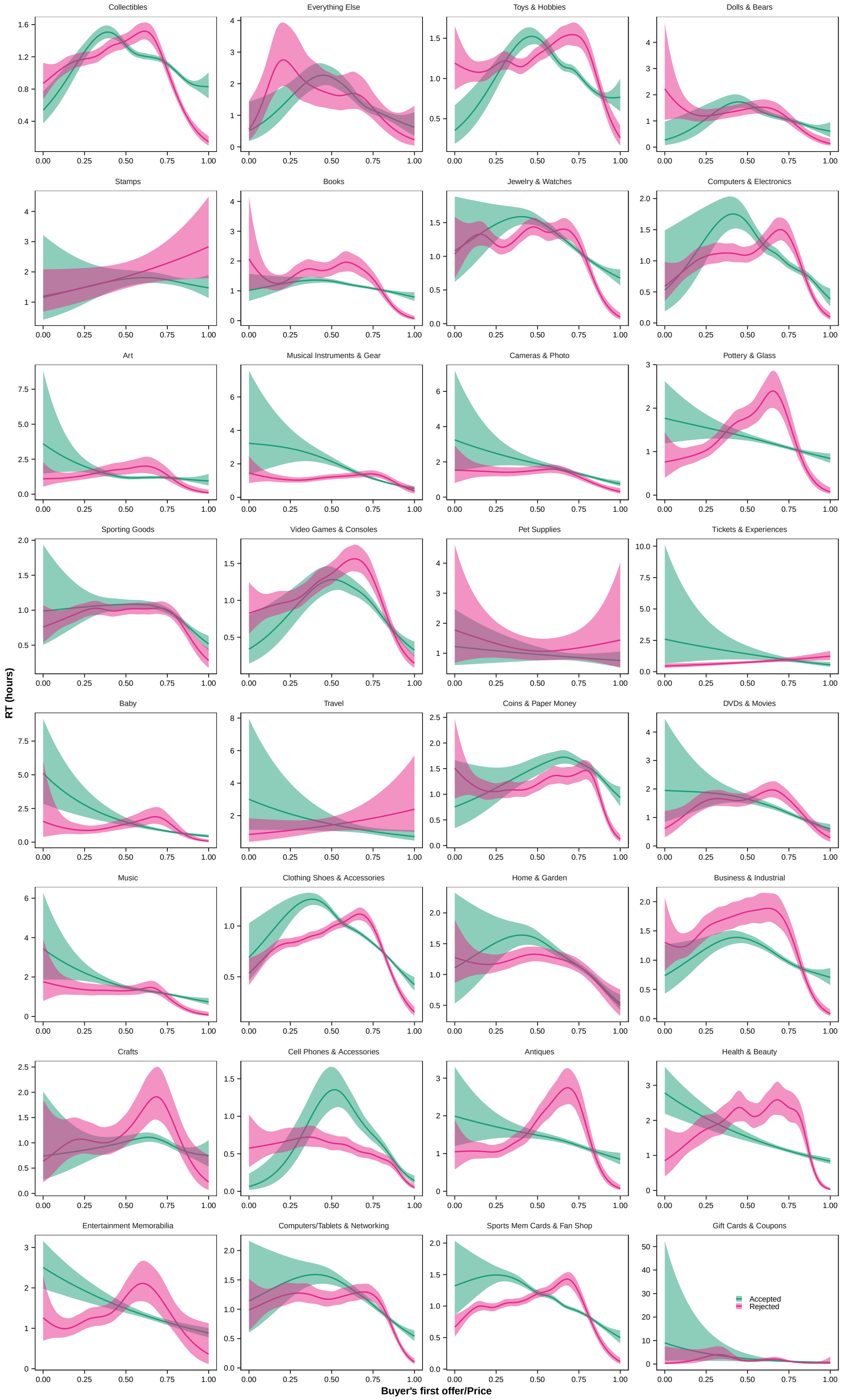
Collectibles
Everything Else
Toys & Hobbies
Dolls & Bears
Stamps
Books
Jewelry & Watches
Computers & Electronics
Art
Musical Instruments & Gear
Cameras & Photo
Pottery & Glass
Sporting Goods
Video Games & Consoles
Pet Supplies
Tickets & Experiences
Baby
Travel
Coins & Paper Money
DVDs & Movies
Music
Clothing Shoes & Accessories
Home & Garden
Business & Industrial
Crafts
Cell Phones & Accessories
Antiques
Health & Beauty
Entertainment Memorabilia
Computers/Tablets & Networking
Sports Mem Cards & Fan Shop
Gift Cards & Coupons
Accepted
Rejected
RT (hours)
Buyer's first offer/Price

Figure S7. GAM regression predictions for each item category for eBay observational data. GAM regression model predictions to sellers' RT (in hours) for buyers' initial offers as a fraction of the sellers' list prices, conditional on the seller accepting or rejecting the offers, broken down at the item category level. Shaded regions represent 95% confidence intervals.

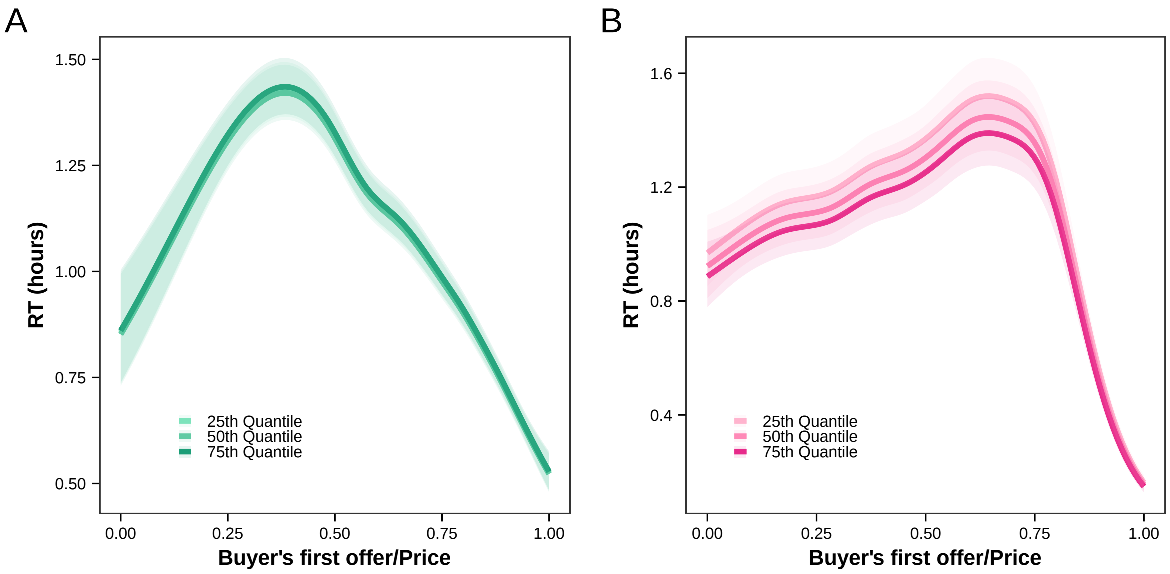

Figure S8. GAM regression predictions for different levels of list price for eBay observational data. (A) Acceptance RT. (B) Rejection RT. The shaded regions represent 95% confidence intervals. The levels of list price used for the predictions are: the 25th quantile = 17 dollars, 50th quantile = 39.95 dollars, 75th quantile = 99.99 dollars.

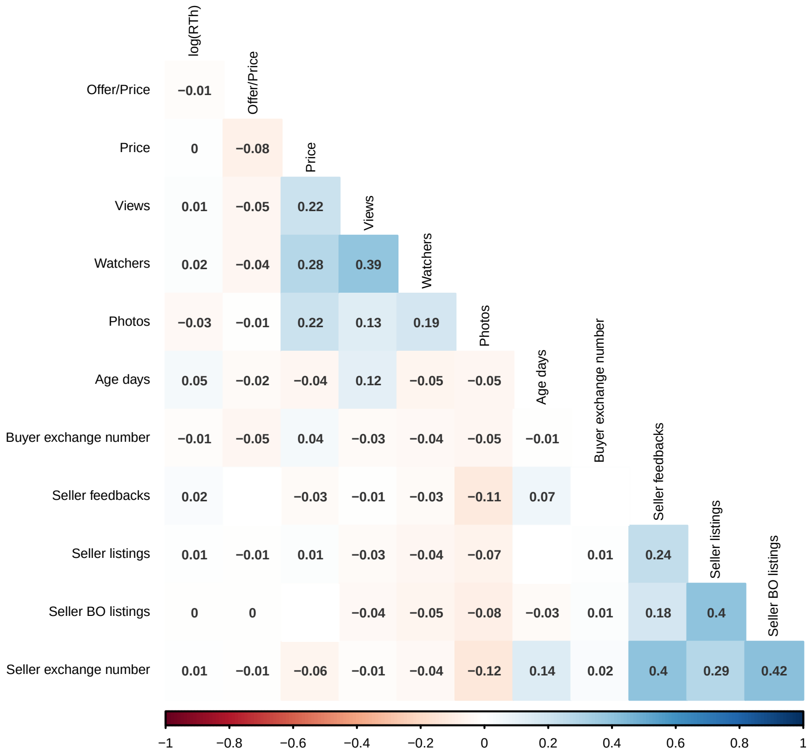

Figure S9. Correlations between variables used in mixed effects regression models for eBay observational data. Offer characteristics variables: Offer/Price - buyer's first offer as a percent of list price; log(RTh) - RT of the seller to the offer in hours. Item characteristics variables: Price - list price; Views - number of views; Watchers - number of watchers; Photos - number of photos; Age days - the listing age in days; Seller and buyer characteristics variables: Buyer exchange number - number of previous best offer exchanges buyer has participated in; Seller feedbacks - seller's number of previous feedbacks received at the time of the offer; Seller listings - number of listings created by the seller dating back to 2008; Seller BO listings - number of Best-Offer-listings created by the seller dating back to 2008; Seller exchange number - number of previous best offer exchanges seller has participated in. Only significant correlations ($p < 0.01$) are displayed. $r = 0$ indicates correlation is smaller than 0.005.

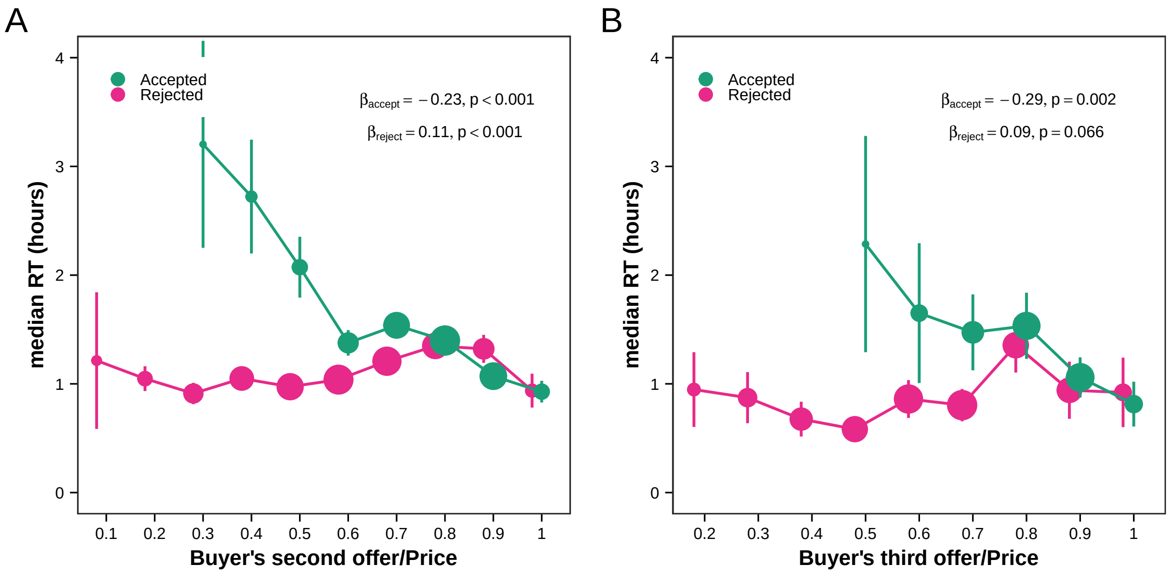

Figure S10. Sellers' median RT as a function of buyers' additional offers for eBay observational data. (A) Sellers' median RT (in hours) for buyers' second offers as a fraction of the sellers' list prices, conditional on the seller accepting or rejecting the offers. (B) Sellers' median RT (in hours) for buyers' third offers as a fraction of the sellers' list prices, conditional on the seller accepting or rejecting the offers. The size of the dots indicates the relative amount of data in that bin, across both curves, and the bars represent bootstrapped standard errors. Bins with less than 25 observations were excluded.

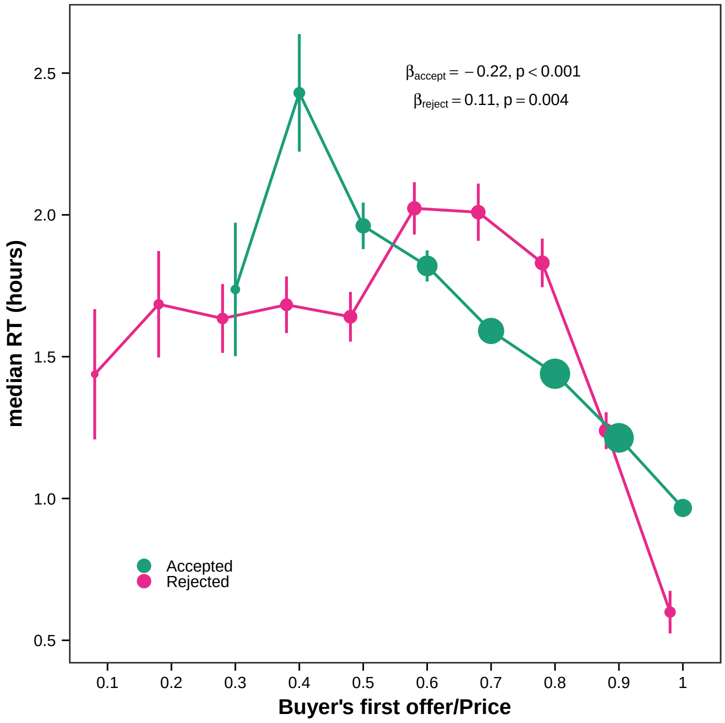

Figure S11. Sellers' median RT for buyers' initial offers by sellers' response for exchanges with thresholds for eBay observational data. Sellers' median RT (in hours) as a function of buyers' initial offers as a percent of list price (p_1/p_0), conditional on the seller accepting or rejecting the offers for bargaining exchanges with either rejection or acceptance threshold and offers that were between these thresholds. The size of the dots indicates the relative amount of data in that bin, across both curves, and the bars represent bootstrapped standard errors. Bins with less than 100 observations were excluded. The coefficients are from a linear regression of log(RT) on first buyer offer ratio (p_1/p_0) (z-score) conditional on the seller accepting, or rejecting the offers using a restricted offer range ($p_1/p_0 = [0.36, 0.68]$) similar to the analyses of observations without thresholds. The regressions also includes random effects (clustered by seller) on the intercept and first buyer offer ratio.

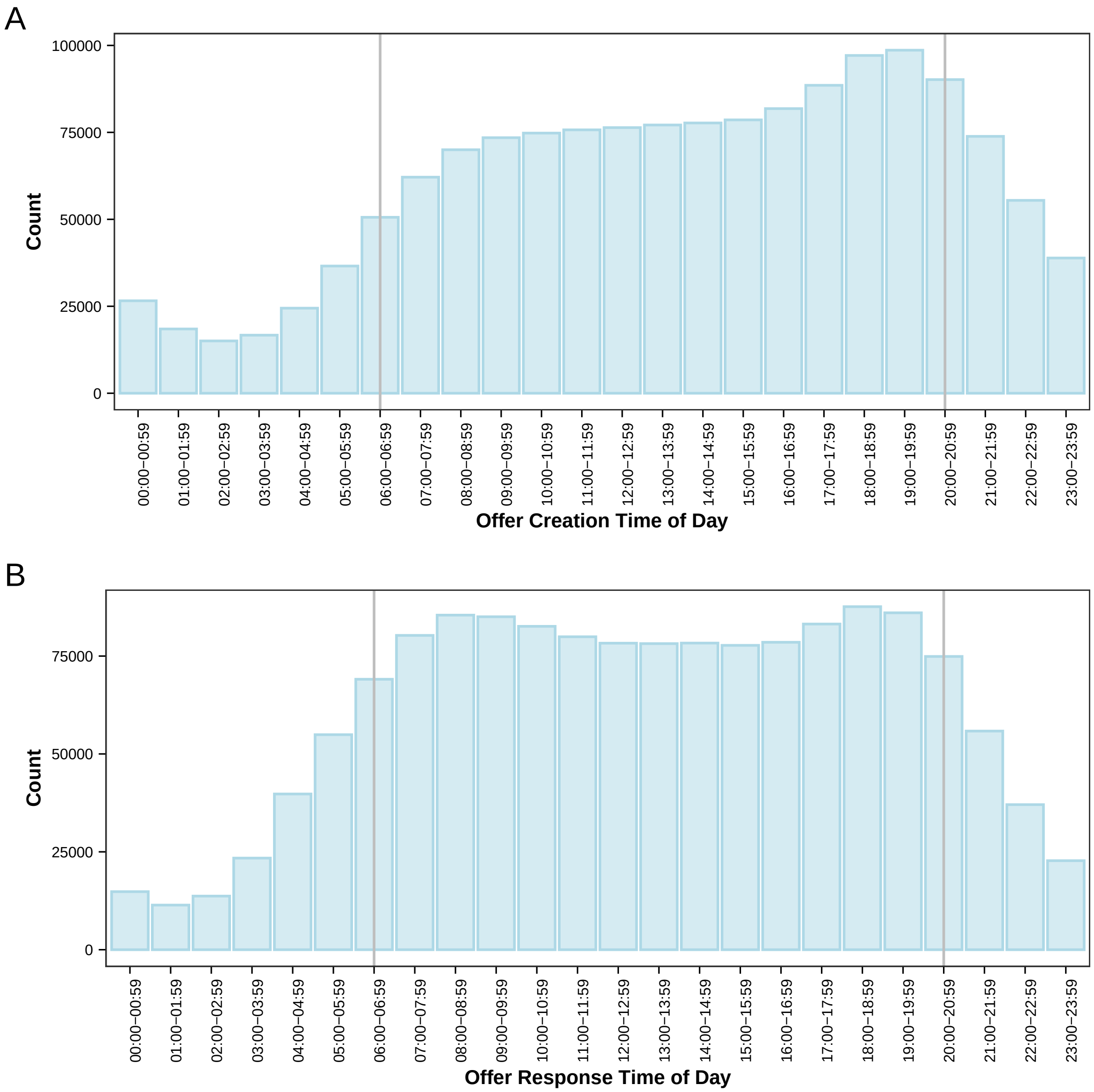

Figure S12. Activity levels by time of day for eBay observational data. (A) Histogram with number of buyer's first offer creation as a function of hour in the day (PT). (B) Histogram with number of seller's first offer responses as a function of hour in the day (PT).

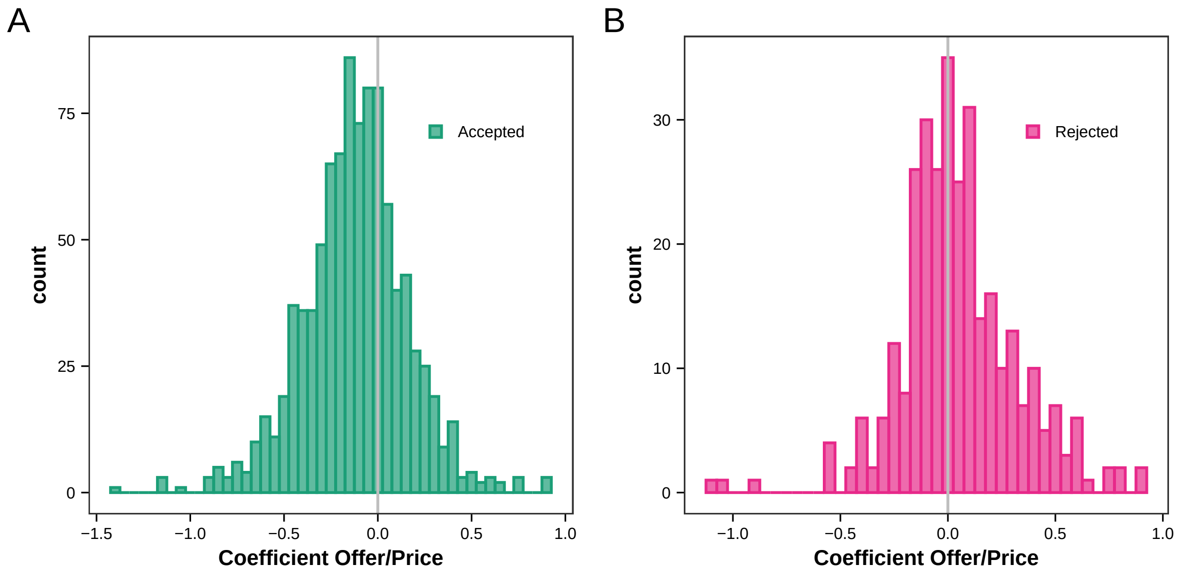

Figure S13. Seller level RT effects for eBay observational data. (A) Histogram of coefficients of buyer's first offer ratio as a fraction of the sellers' list prices at the seller level for acceptance log RT in hours. (B) Histogram of coefficients of buyer's first offer ratio as a fraction of the sellers' list prices at the seller level for rejection log RT in hours. Only sellers with more than 50 acceptances or more than 50 rejections were included.

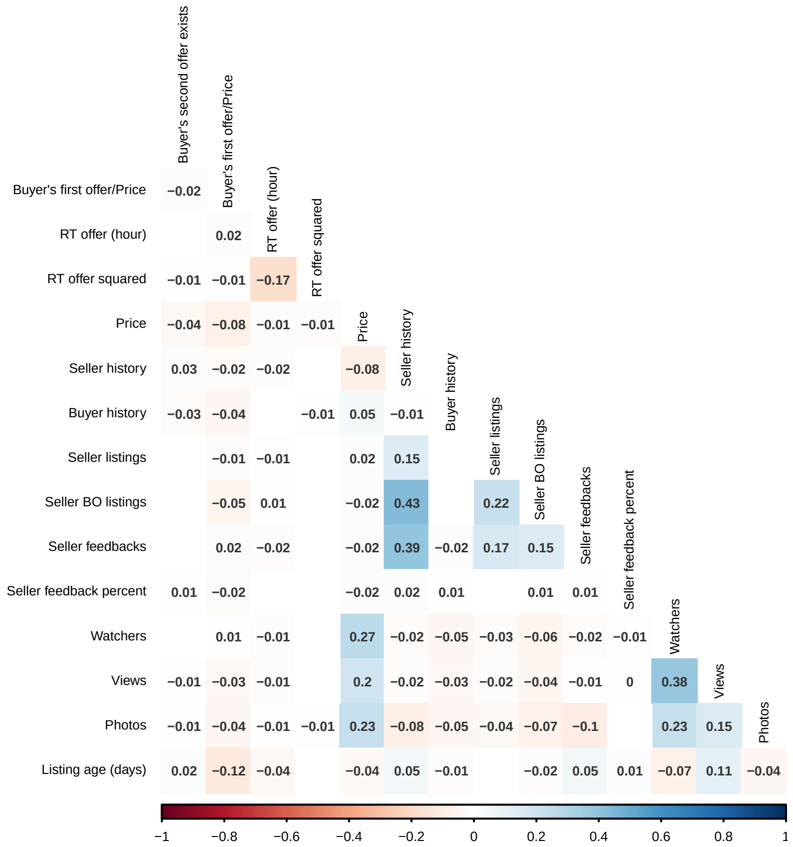

Figure S14. Correlations between existence of second buyer offer and other variables for eBay observational data. Offer characteristics variables: Buyer's second offer exists - Whether buyer's second offer exists; Buyer's first offer/Price - buyer's first offer as a percent of list price; RT offer (hours) - RT of the seller to the offer in hours; RT offer squared - squared RT of the seller to the offer in hours. Item characteristics variables: Price - list price; Views - number of views; Watchers - number of watchers; Photos - number of photos; Listing age (days) - the listing age in days; Seller and buyer characteristics variables: Buyer history - number of previous best offer exchanges buyer has participated in; Seller feedbacks - seller's number of previous feedbacks received at the time of the offer; Seller listings - number of listings created by the seller dating back to 2008; Seller BO listings - number of Best-Offer-listings created by the seller dating back to 2008; Seller history - number of previous best offer exchanges seller has participated in. Only significant correlations ($p < 0.01$) are displayed.

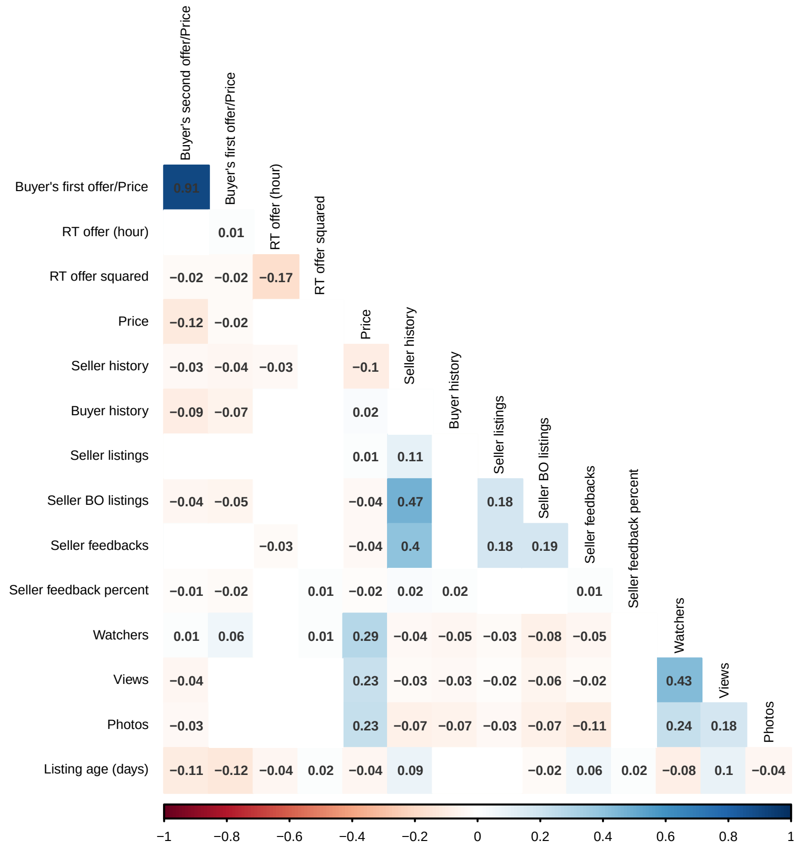

Figure S15. Correlations between second buyer offer amount and other variables for eBay observational data. Offer characteristics variables: Buyer's second offer/Price - buyer's second offer as a percent of list price; Buyer's first offer/Price - buyer's first offer as a percent of list price; RT offer (hours) - RT of the seller to the offer in hours; RT offer squared - squared RT of the seller to the offer in hours. Item characteristics variables: Price - list price; Views - number of views; Watchers - number of watchers; Photos - number of photos; Listing age (days) - the listing age in days; Seller and buyer characteristics variables: Buyer history - number of previous best offer exchanges buyer has participated in; Seller feedbacks - seller's number of previous feedbacks received at the time of the offer; Seller listings - number of listings created by the seller dating back to 2008; Seller BO listings - number of Best-Offer-listings created by the seller dating back to 2008; Seller history - number of previous best offer exchanges seller has participated in. Only significant correlations ($p < 0.01$) are displayed.

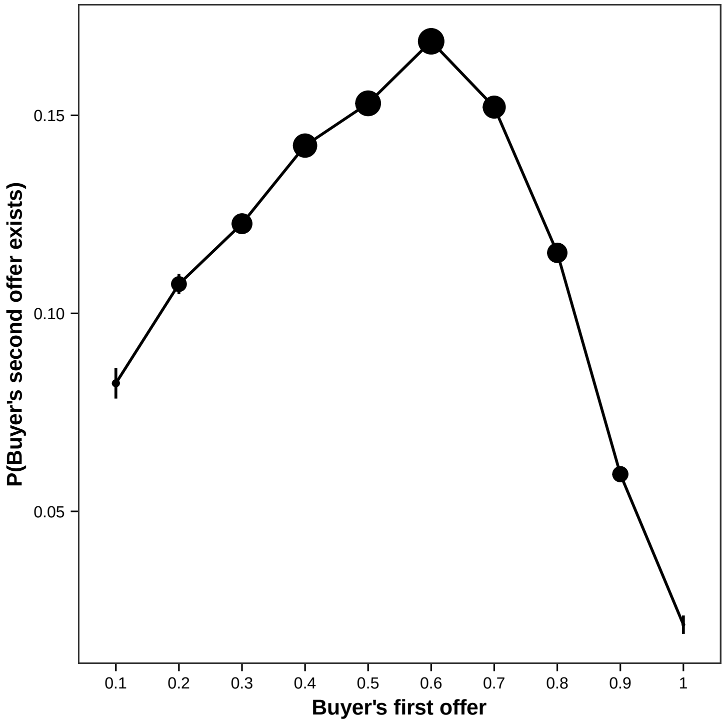

Figure S16. Second buyer offer existence probability for eBay observational data. Probability of second buyer offer existence as a function of first buyer's offer as a fraction of seller's list price.

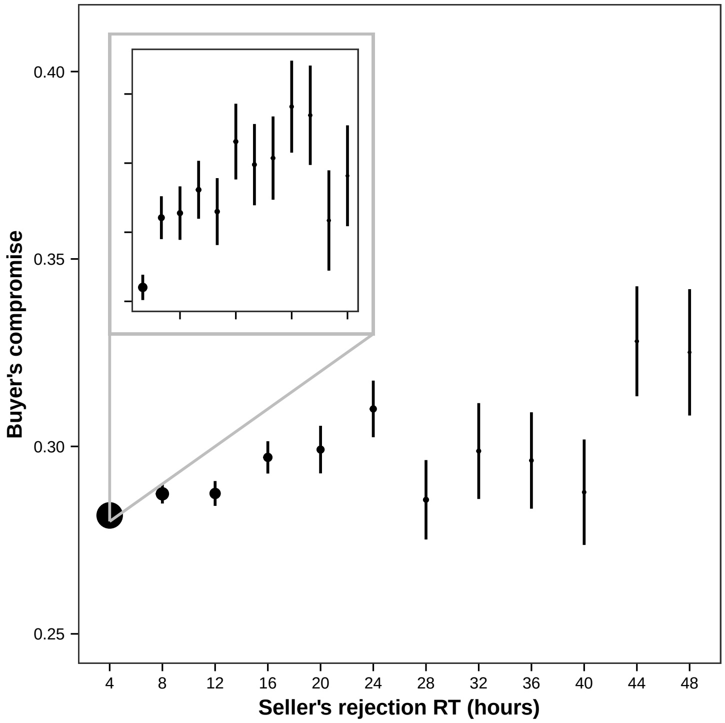

Figure S17. Buyers' compromise depending on sellers' RT. Size of buyers' compromise as a function of sellers' rejection RT to the first offers. The buyer's compromise is the amount that they raised their second offer, divided by the gap between the list price and buyer's first offer. A 100% compromise would be a second offer that is the list price; a 0% compromise would be a second offer that is the same as the first offer. In the inset zoom, the buyer's compromise ranges from $[0.272, 0.298]$ The size of the dots indicates the relative amount of data in that bin and the bars represent bootstrapped standard errors across buyers.

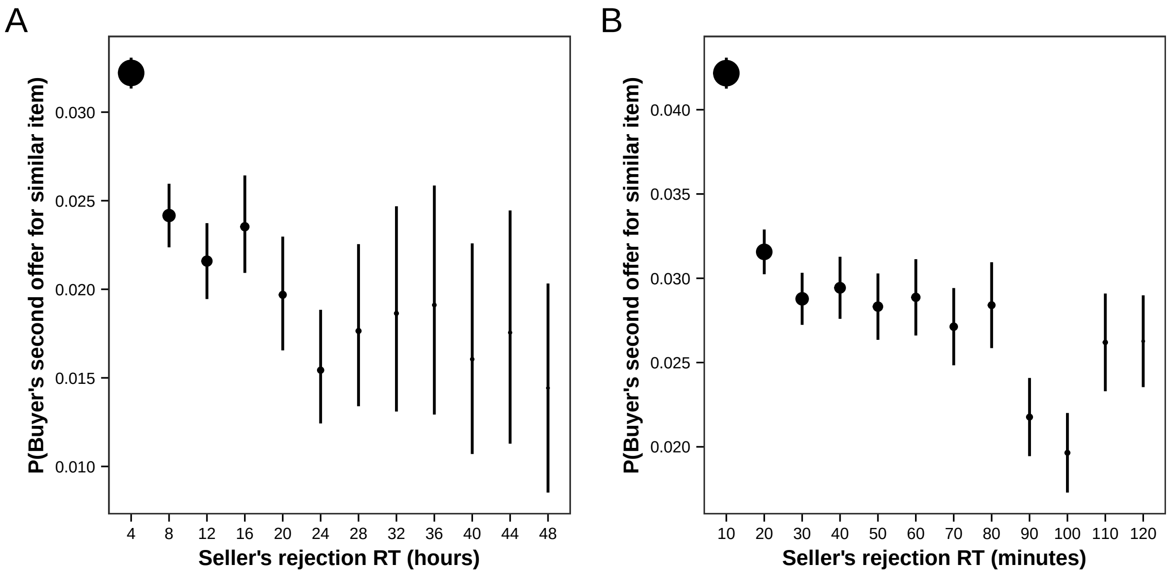

Figure S18. The probability of a buyer making an offer to a different seller on a similar item (in the same category) within 24 hours of their first offer being rejected, as a function of the seller's rejection RT. The faster the seller rejects the buyer's first offer, the more likely they are to turn to another seller. (A) Entire range of seller rejections RT (hours) (B) Zooming in on seller rejection RT (minutes) between 0-2 hours.

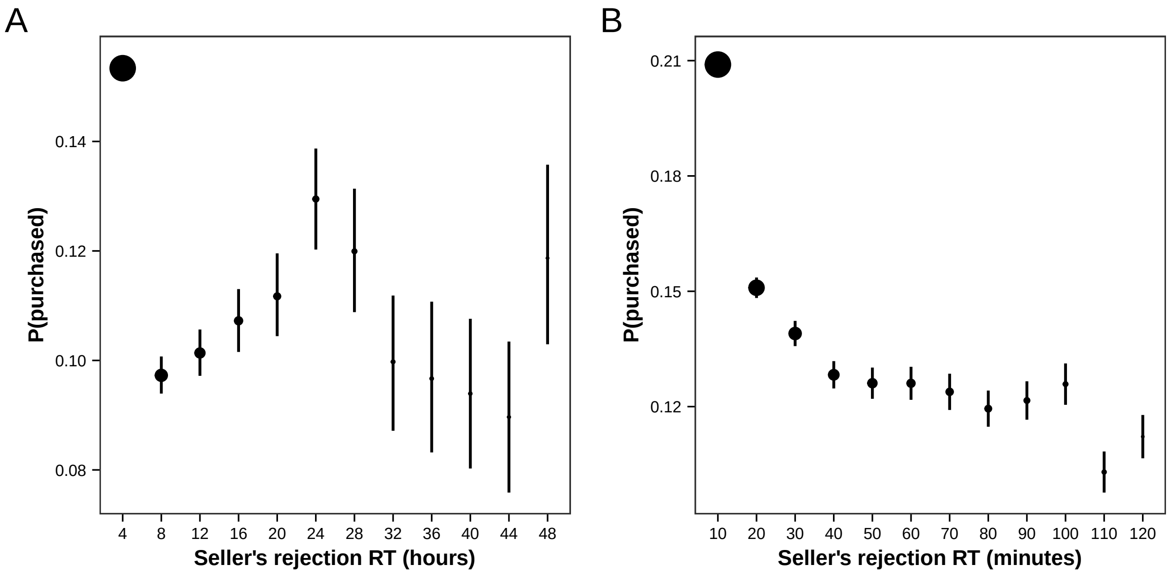

Figure S19. The probability of a buyer purchasing an item at the list price as a function of the seller's rejection RT to their first offer. (A) Entire range of seller rejections RT (hours) (B) Zooming in on the seller rejection RT (minutes) between 0-2 hours.

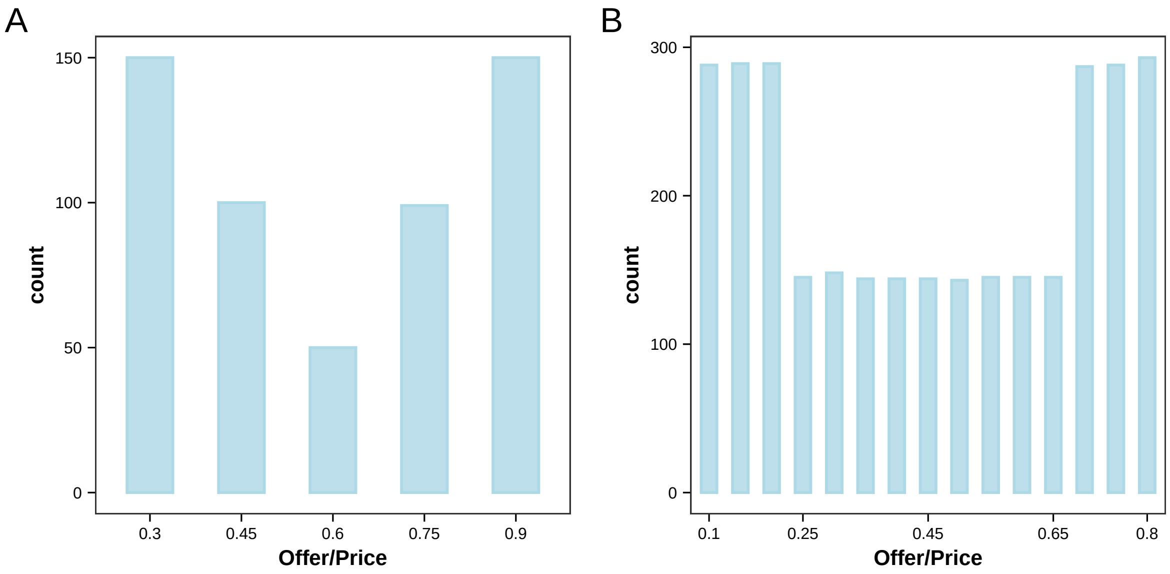

Figure S20. Histograms for offer ratios made in the eBay field experiments. First offer as a percent of seller's list price. (A) Experiment 1. (B) Experiment 2.

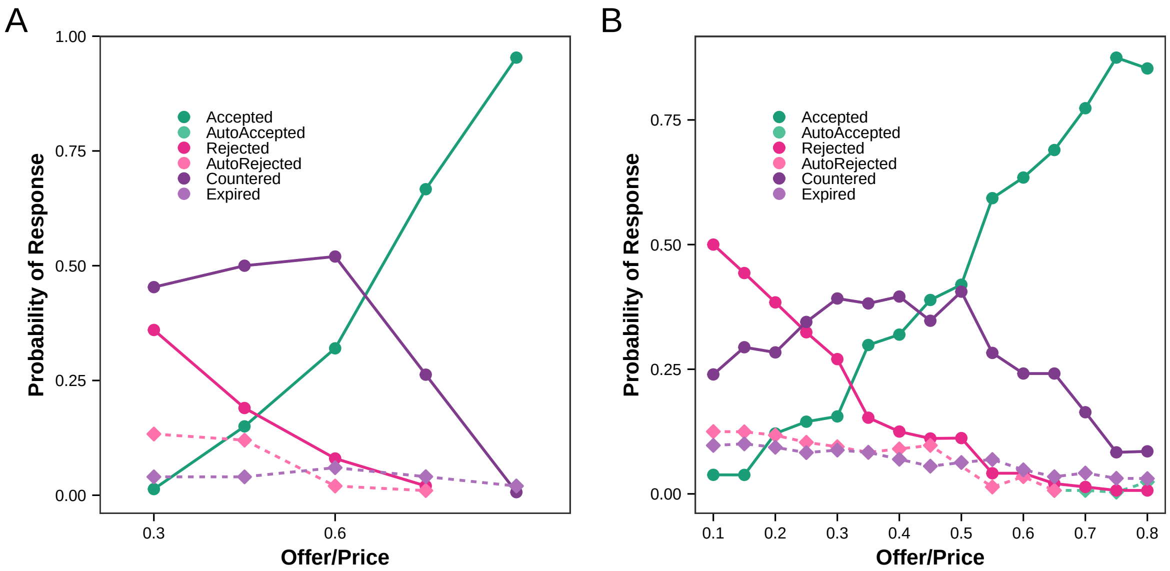

Figure S21. Probability of each type of seller response for the eBay field experiments. (A) Experiment 1. (B) Experiment 2.

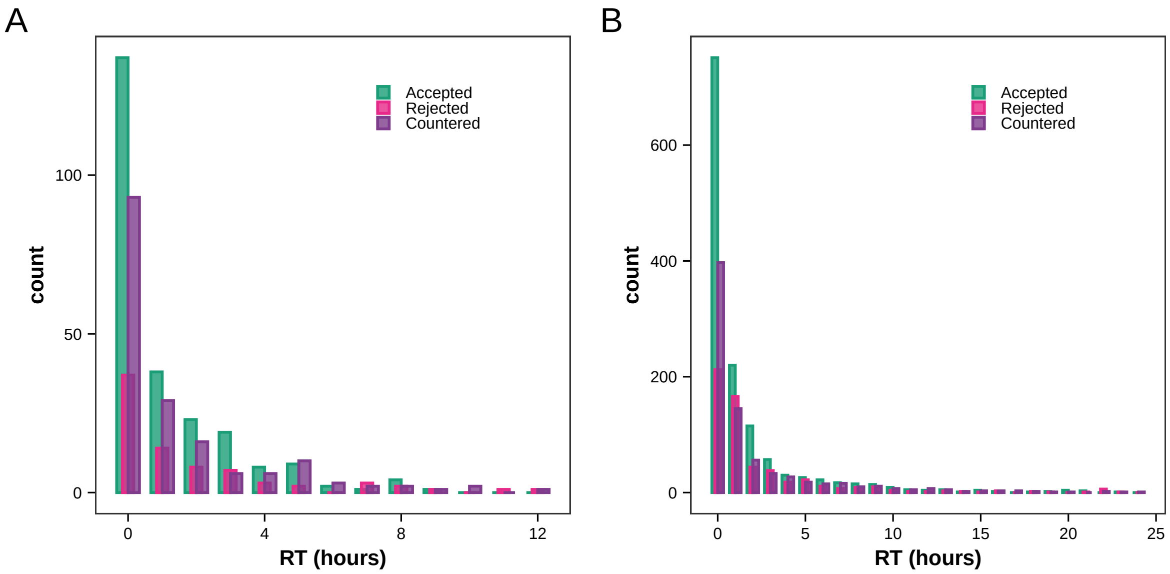

Figure S22. Response time distributions for seller's response conditional on the type of response for the eBay field experiments. (A) Experiment 1. (B) Experiment 2.

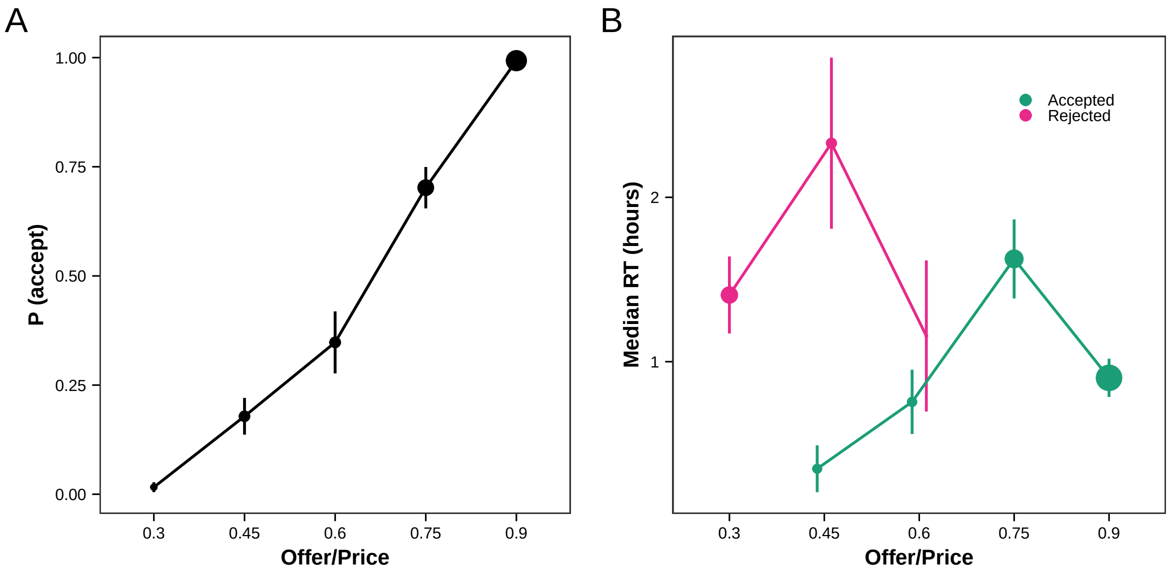

Figure S23. Choice and median RT as a function of buyer's first offer for the eBay field experiment 1. (A) Probability of seller's response by offer ratio conditional on the type of response. (B) Sellers' median RT (in hours) as a function of of buyers' initial offers, as a fraction of the sellers' list prices, conditional on the seller accepting or rejecting the offers. Only bin sizes with more than 2 data points were included. The size of the dots indicates the relative amount of data in that bin, across both curves, and the bars represent bootstrapped standard errors across sellers.

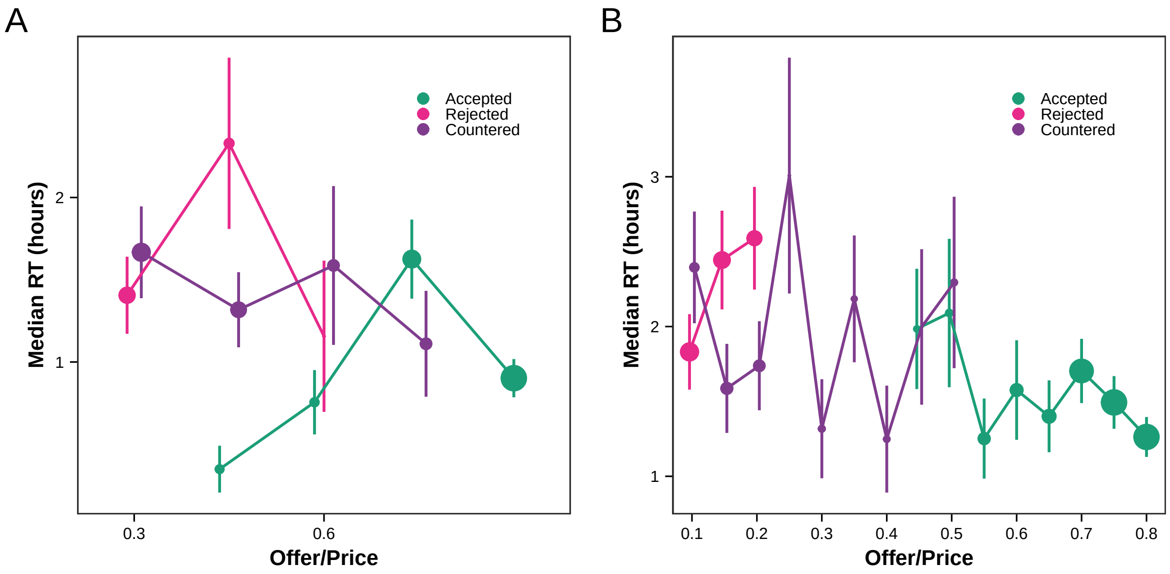

Figure S24. RT (hours) as a function of offer ratio. (A) Experiment 1. (B) Experiment 2.

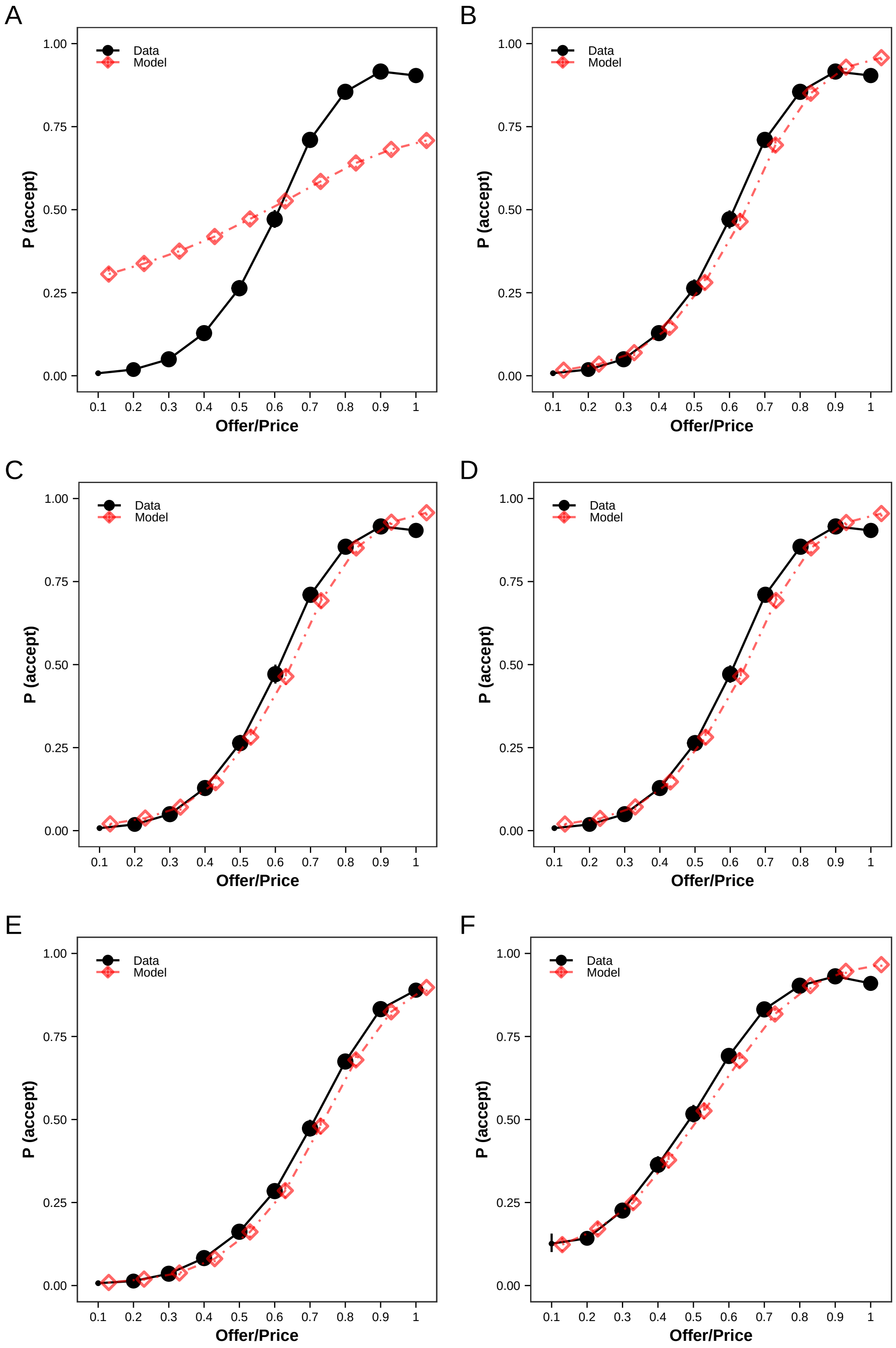
A
B
C
D
E
F
Data
Model
P (accept)
Offer/Price
1.00
0.75
0.50
0.25
0.00
0.1 0.2 0.3 0.4 0.5 0.6 0.7 0.8 0.9 1

Figure S25. Probability of acceptance for data versus model predictions for eBay observational data. Sellers' probability of accepting the first offer as a function of the buyer's first offer as a fraction of seller's list price. (A) Standard DDM. (B) Gamma DDM. (C) Time of Day Gamma DDM. (D) Time of Day and Offer Ratio Gamma DDM. (E) Best fitting model for each seller pooling counteroffers with rejections. (F) Best fitting model for each seller pooling counteroffers with acceptances.

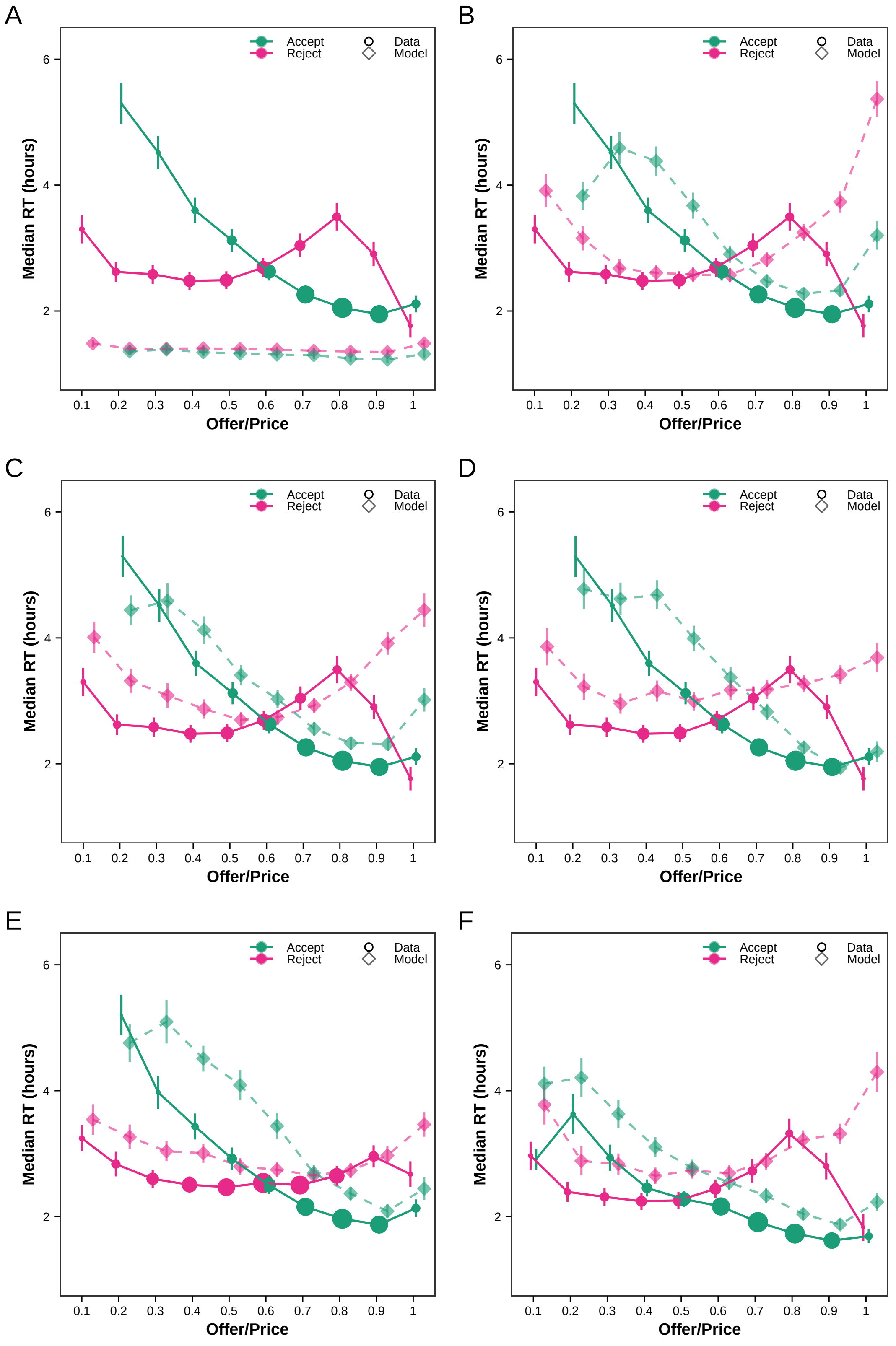

A
B
C
D
E
F
Accept
Reject
Data
Model
Median RT (hours)
Offer/Price
0.1
0.2
0.3
0.4
0.5
0.6
0.7
0.8
0.9
1
2
4
6

Figure S26. Median RT for data versus model predictions for eBay observational data. Seller's median RT (in hours) as a function of the buyer's first offer as a fraction of seller's list price, in the data and DDM fits. (A) Standard DDM. (B) Gamma DDM. (C) Time of Day Gamma DDM. (D) Time of Day and Offer Ratio Gamma DDM. Bins with less than 12 observations were excluded. (E) Best fitting model for each seller pooling counteroffers with rejections. (F) Best fitting model for each seller pooling counteroffers with acceptances. Bins with less than 100 observations were excluded.

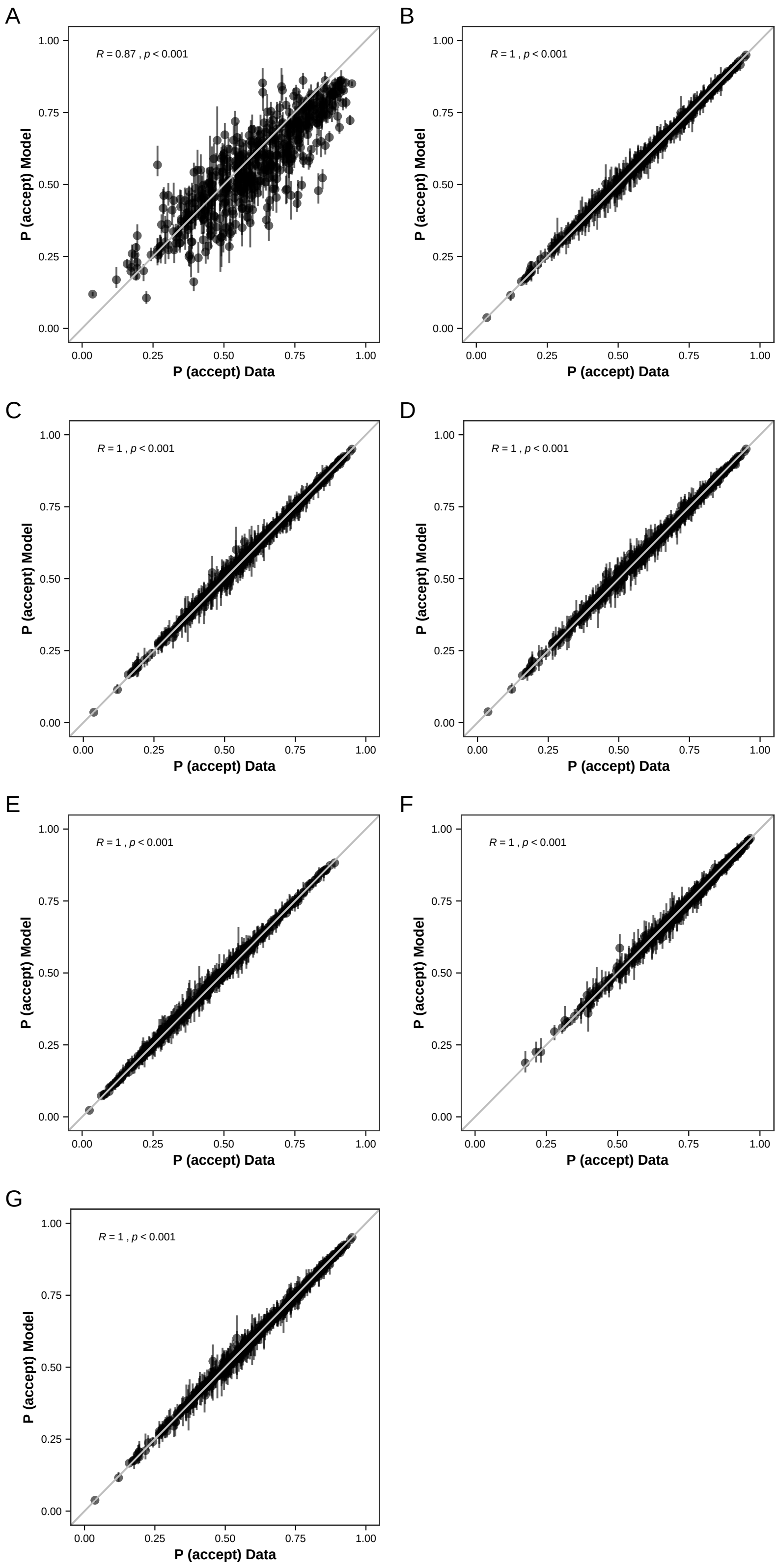
A
R = 0.87 , p < 0.001
B
R = 1 , p < 0.001
C
R = 1 , p < 0.001
D
R = 1 , p < 0.001
E
R = 1 , p < 0.001
F
R = 1 , p < 0.001
G
R = 1 , p < 0.001
P (accept) Model
P (accept) Data
0.00
0.25
0.50
0.75
1.00

Figure S27. Probability of acceptance at the subject level for data versus model predictions for eBay observational data. The bars represent 95% HDIs for 10 simulations per trial using the mean posterior of the best fitting subject level parameters. (A) Standard DDM. (B) Gamma DDM. (C) Time of Day Gamma DDM. (D) Time of Day and Offer Ratio Gamma DDM. (E) Best fitting model for each seller pooling counteroffers with rejections. (F) Best fitting model for each seller pooling counteroffers with acceptances. (G) Best fitting model for each seller.

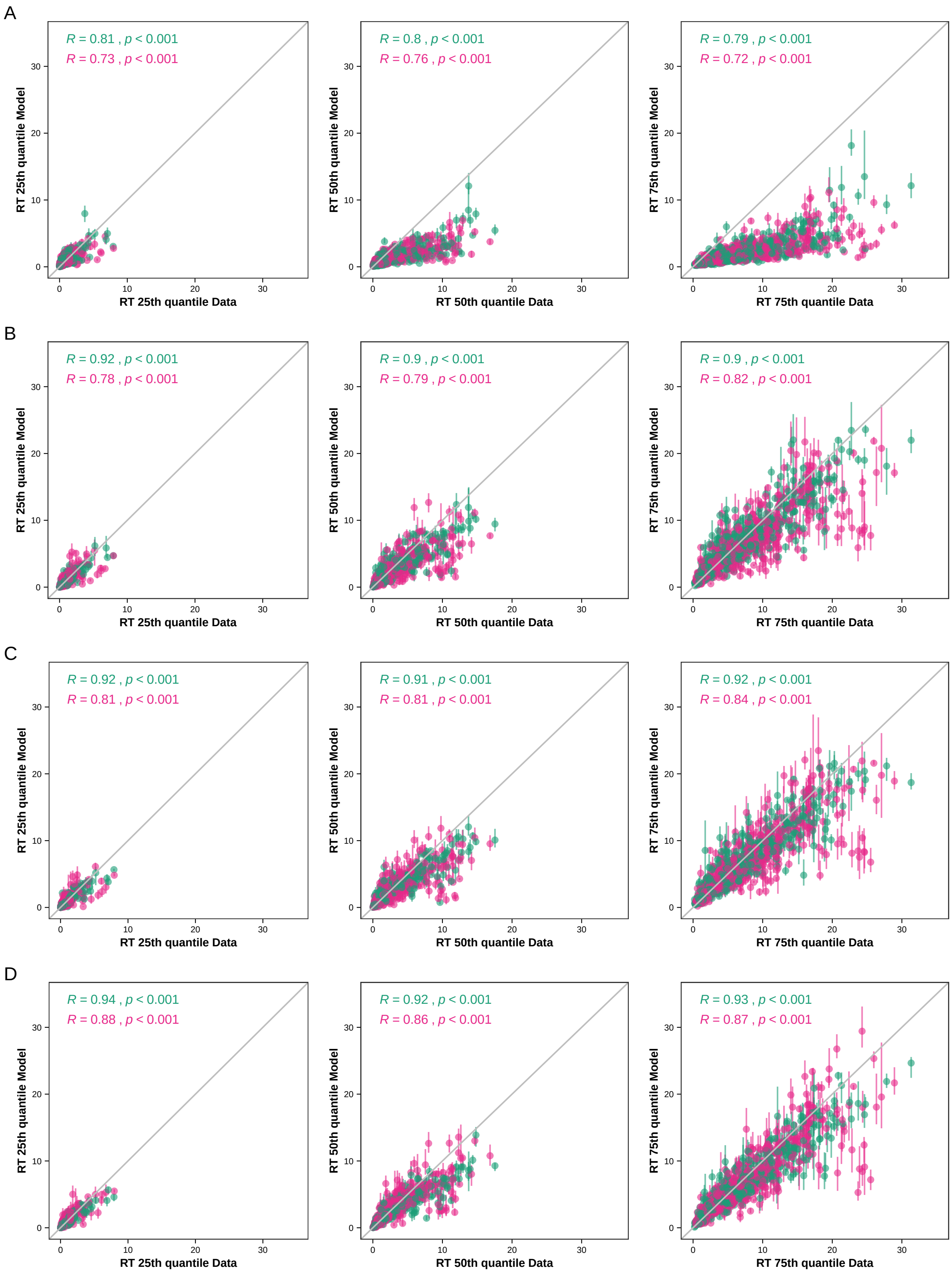
A
R = 0.81 , p < 0.001
R = 0.73 , p < 0.001
RT 25th quantile Model
RT 25th quantile Data
R = 0.8 , p < 0.001
R = 0.76 , p < 0.001
RT 50th quantile Model
RT 50th quantile Data
R = 0.79 , p < 0.001
R = 0.72 , p < 0.001
RT 75th quantile Model
RT 75th quantile Data
B
R = 0.92 , p < 0.001
R = 0.78 , p < 0.001
R = 0.9 , p < 0.001
R = 0.79 , p < 0.001
R = 0.9 , p < 0.001
R = 0.82 , p < 0.001
C
R = 0.92 , p < 0.001
R = 0.81 , p < 0.001
R = 0.91 , p < 0.001
R = 0.81 , p < 0.001
R = 0.92 , p < 0.001
R = 0.84 , p < 0.001
D
R = 0.94 , p < 0.001
R = 0.88 , p < 0.001
R = 0.92 , p < 0.001
R = 0.86 , p < 0.001
R = 0.93 , p < 0.001
R = 0.87 , p < 0.001

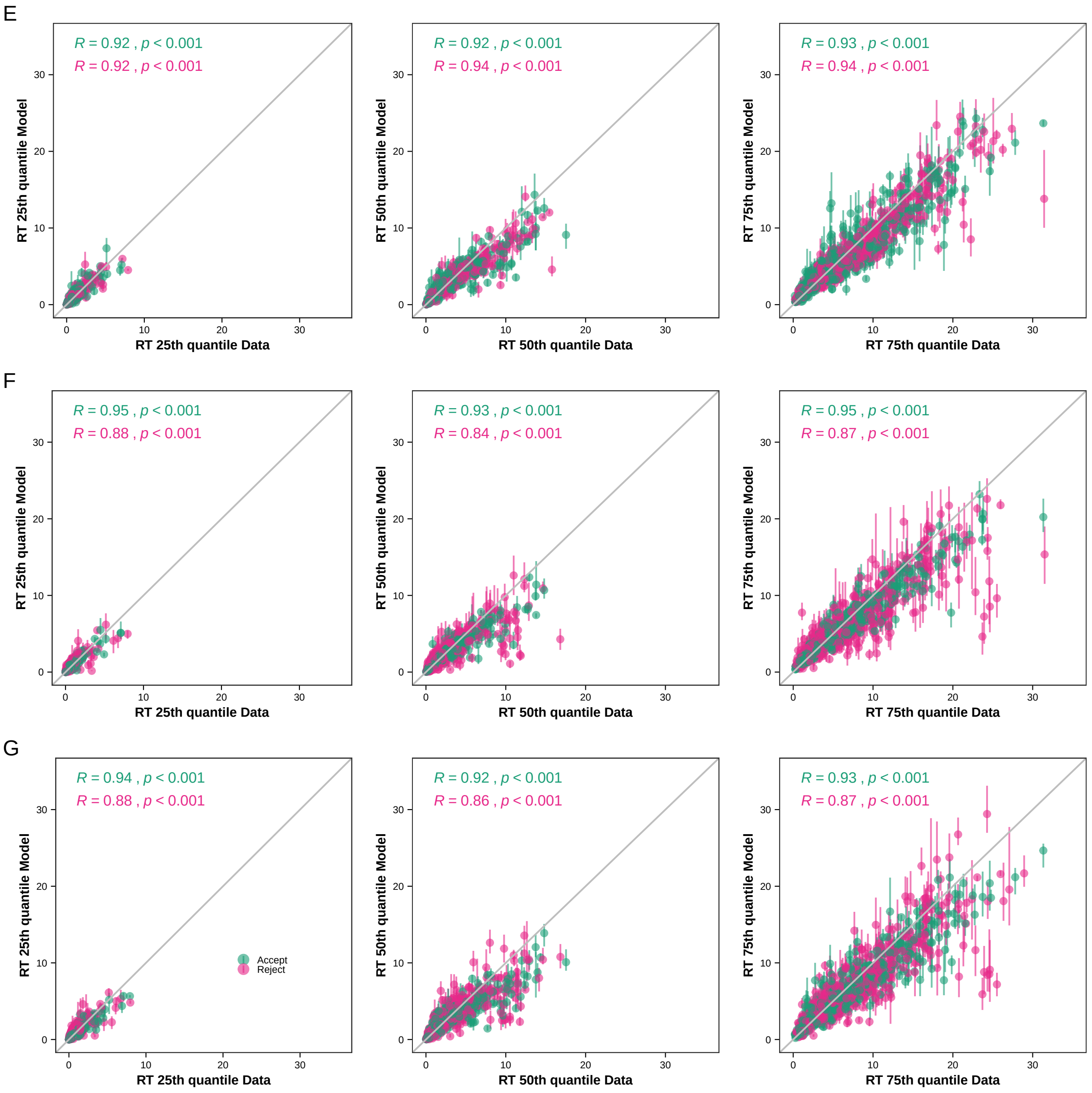

Figure S28. Response time quartiles at the subject level for data versus model predictions for acceptances and rejections for eBay observational data. The bars represent 95% HDIs for 10 simulations per trial using the mean posterior of the best fitting subject level parameters. (A) Standard DDM. (B) Gamma DDM. (C) Time of Day Gamma DDM. (D) Time of Day and Offer Ratio Gamma DDM. (E) Best fitting model for each seller pooling counteroffers with rejections. (F) Best fitting model for each seller pooling counteroffers with acceptances. (G) Best fitting model for each seller.

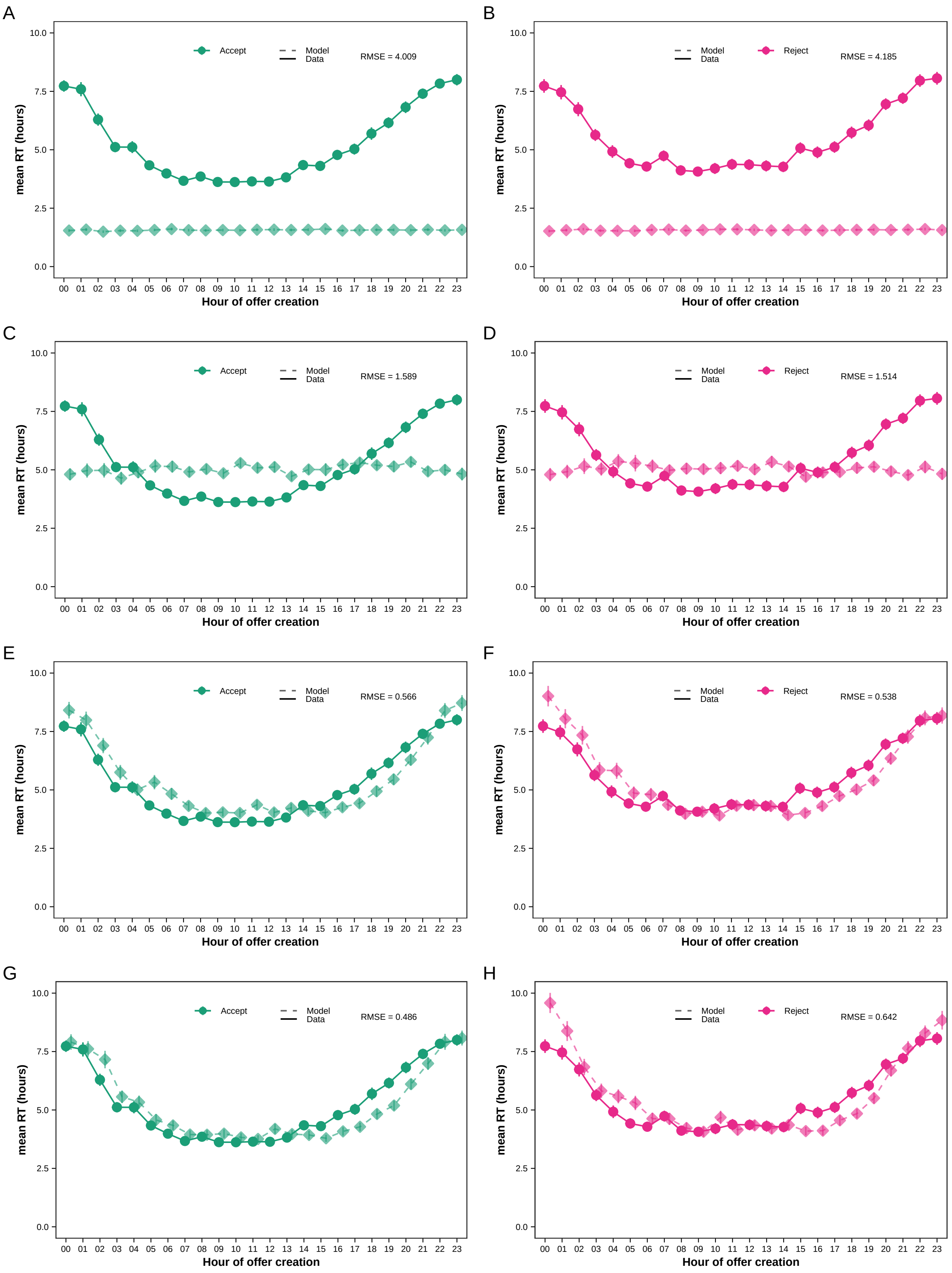
A
Accept
Model
Data
RMSE = 4.009
mean RT (hours)
Hour of offer creation
B
Reject
Model
Data
RMSE = 4.185
mean RT (hours)
Hour of offer creation
C
Accept
Model
Data
RMSE = 1.589
mean RT (hours)
Hour of offer creation
D
Reject
Model
Data
RMSE = 1.514
mean RT (hours)
Hour of offer creation
E
Accept
Model
Data
RMSE = 0.566
mean RT (hours)
Hour of offer creation
F
Reject
Model
Data
RMSE = 0.538
mean RT (hours)
Hour of offer creation
G
Accept
Model
Data
RMSE = 0.486
mean RT (hours)
Hour of offer creation
H
Reject
Model
Data
RMSE = 0.642
mean RT (hours)
Hour of offer creation

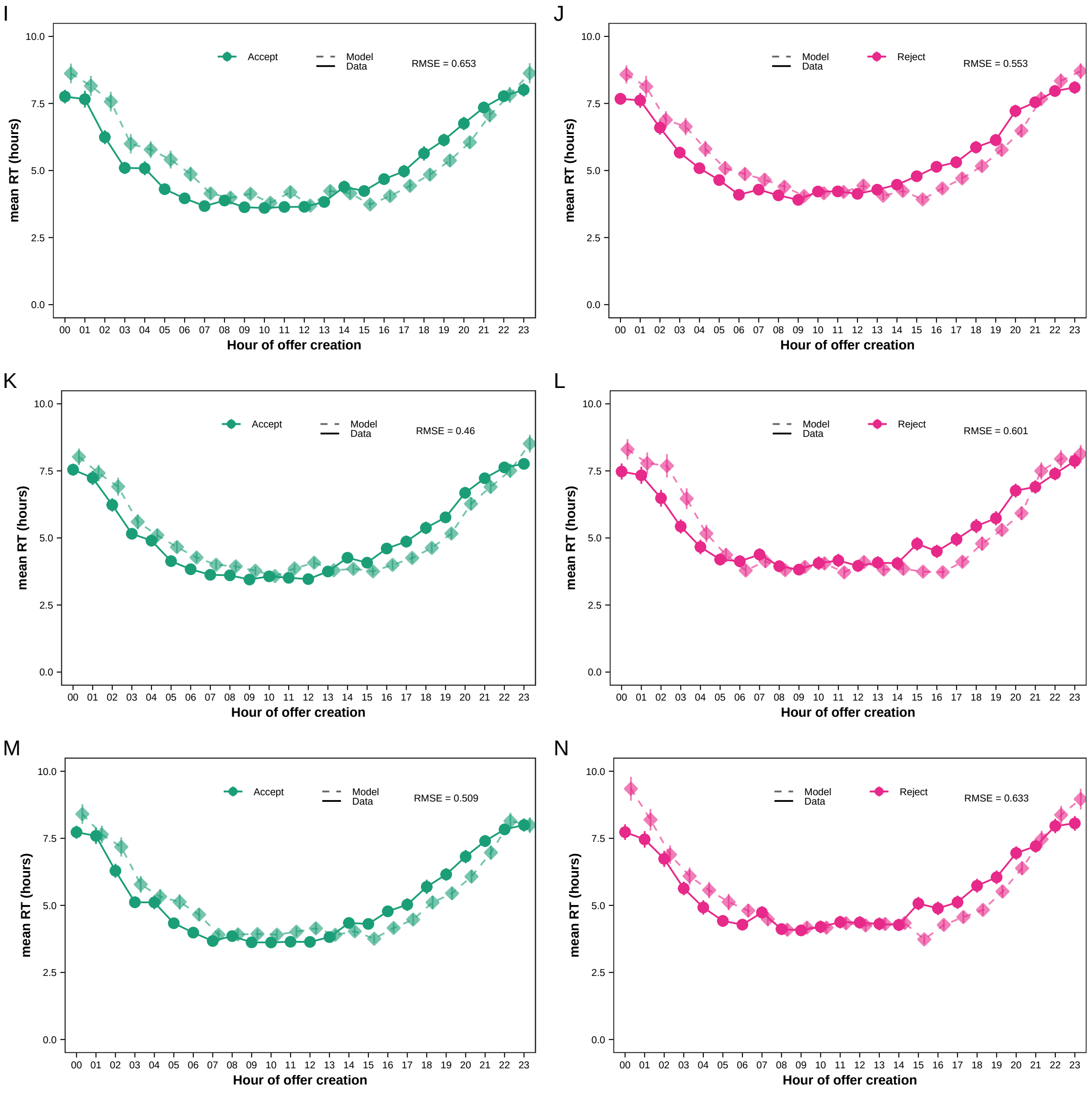

Figure S29. Mean Response time in hours as a function of hour in the day when the offer was created starting at midnight (PT) conditional on acceptance or rejection for the data and the model predictions for eBay observational data. The bars represent 95% HDIs for 10 simulations per trial using the mean posterior of the best fitting subject level parameters. (A), (B) Standard DDM. (C), (D) Gamma DDM. (E), (F) Time of Day Gamma DDM. (G), (H) Time of Day and Offer Ratio Gamma DDM. (I), (J) Best fitting model for each seller pooling counteroffers with rejections. (K), (L) Best fitting model for each seller pooling counteroffers with acceptances. (M), (N) Best fitting model for each seller.

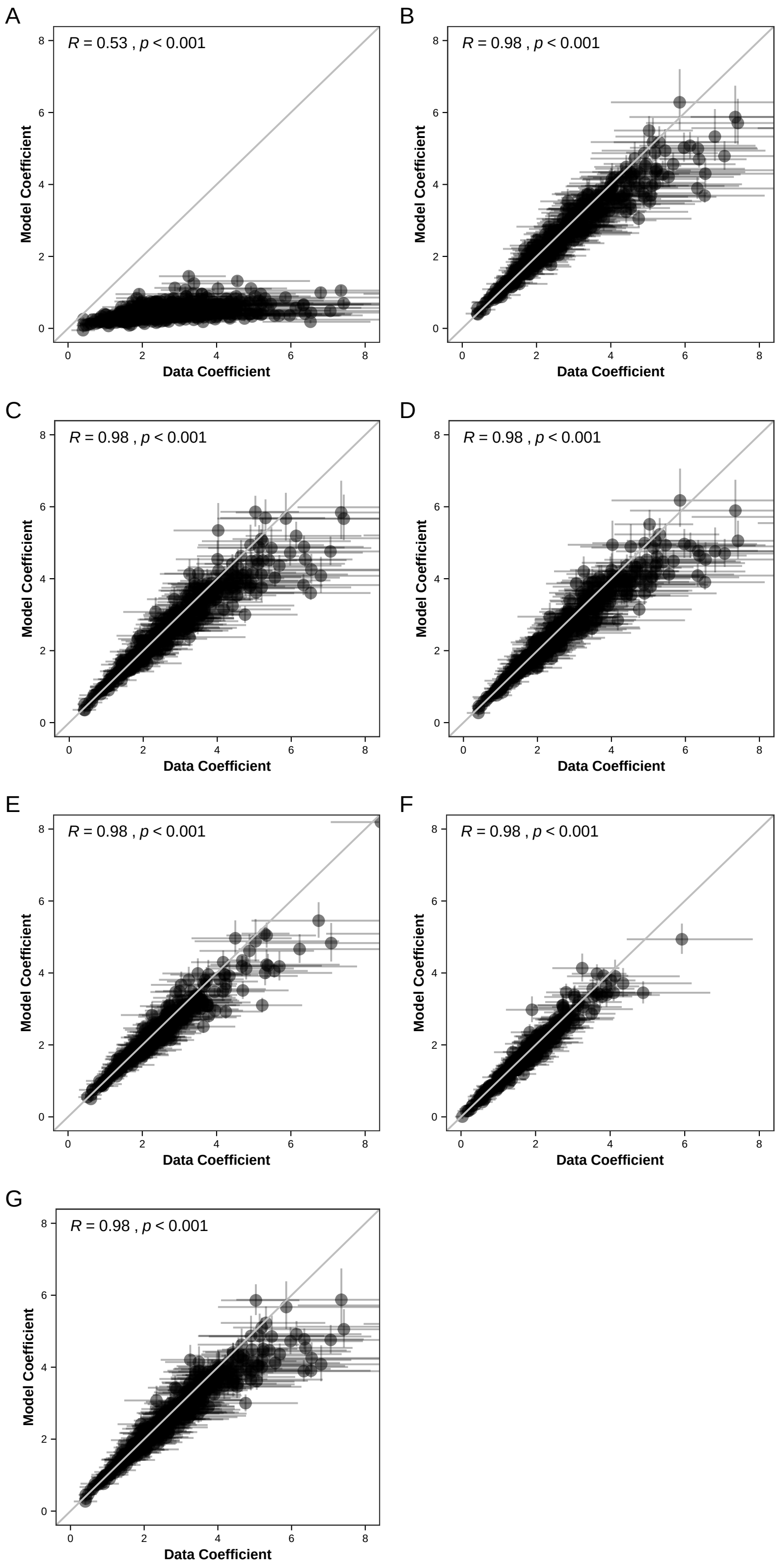
A
R = 0.53 , p < 0.001
B
R = 0.98 , p < 0.001
C
R = 0.98 , p < 0.001
D
R = 0.98 , p < 0.001
E
R = 0.98 , p < 0.001
F
R = 0.98 , p < 0.001
G
R = 0.98 , p < 0.001
Model Coefficient
Data Coefficient
0
2
4
6
8

Figure S30. Model versus data coefficients of acceptance on first offer ratio at the seller level for eBay observational data. The coefficients are from logistic regressions at the seller level using acceptance dummy as dependent variables and z-scored first offer ratio and list price as independent variables. The bars represent 95% confidence intervals. (A) Standard DDM. (B) Gamma DDM. (C) Time of Day Gamma DDM. (D) Time of Day and Offer Ratio Gamma DDM. (E) Best fitting model for each seller pooling counteroffers with rejections. (F) Best fitting model for each seller pooling counteroffers with acceptances. (G) Best fitting model for each seller.

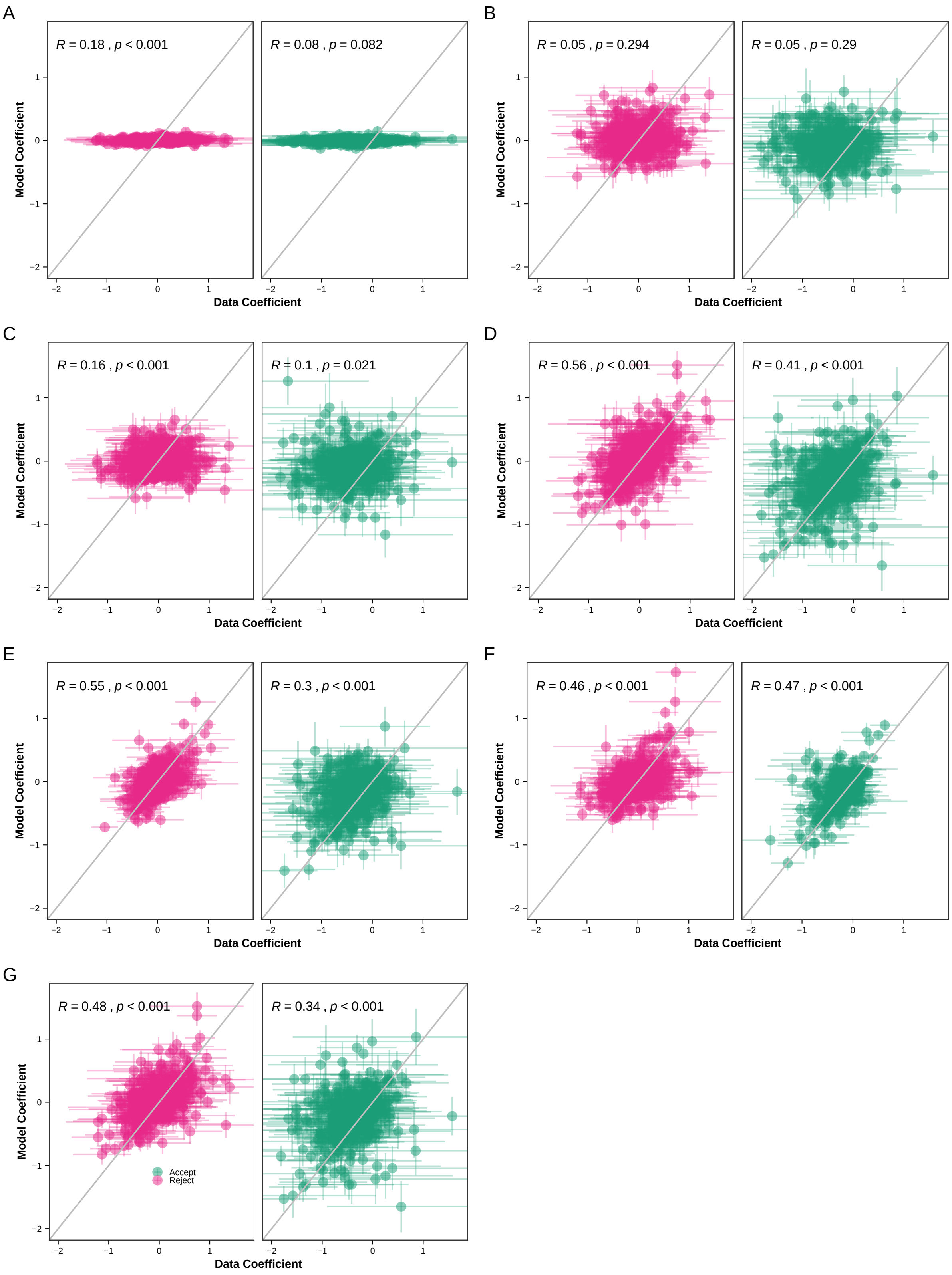
A
R = 0.18 , p < 0.001
R = 0.08 , p = 0.082
B
R = 0.05 , p = 0.294
R = 0.05 , p = 0.29
C
R = 0.16 , p < 0.001
R = 0.1 , p = 0.021
D
R = 0.56 , p < 0.001
R = 0.41 , p < 0.001
E
R = 0.55 , p < 0.001
R = 0.3 , p < 0.001
F
R = 0.46 , p < 0.001
R = 0.47 , p < 0.001
G
R = 0.48 , p < 0.001
R = 0.34 , p < 0.001
Model Coefficient
Data Coefficient
Accept
Reject

Figure S31. Model versus data coefficients of logRT on first offer ratio at the seller level for eBay observational data. The coefficients are from linear regressions at the seller level using log RT in hours as dependent variables and z-scored first offer ratio and list price as independent variables for acceptances and rejections respectively. The bars represent 95% confidence intervals. (A) Standard DDM. (B) Gamma DDM. (C) Time of Day Gamma DDM. (D) Time of Day and Offer Ratio Gamma DDM. (E) Best fitting model for each seller pooling counteroffers with rejections. (F) Best fitting model for each seller pooling counteroffers with acceptances. (G) Best fitting model for each seller.

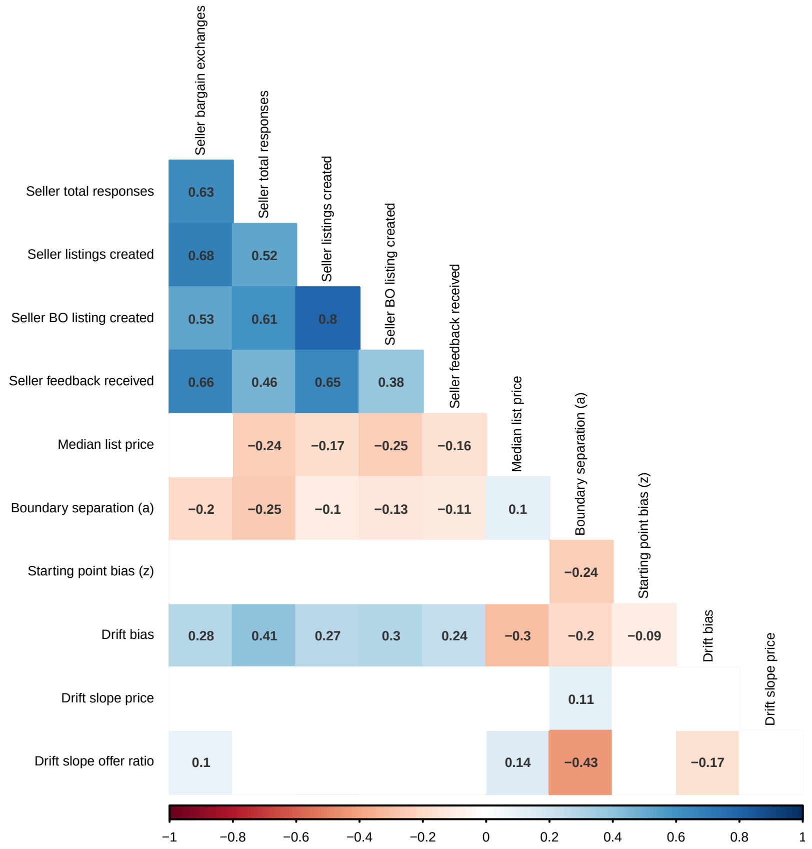

Figure S32. Correlations between mean posteriors of seller model parameters and seller experience using the best fitting model for each seller in the eBay observational data. The seller characteristics are: number of previous best offer exchanges seller has participated in, number of total seller responses (acceptances and rejections), seller's number of previous feedbacks received at the time of the offer, number of listings created by the seller dating back to 2008, number of Best-Offer-listings created by the seller dating back to 2008. Only significant correlations ($p < 0.01$) are displayed.

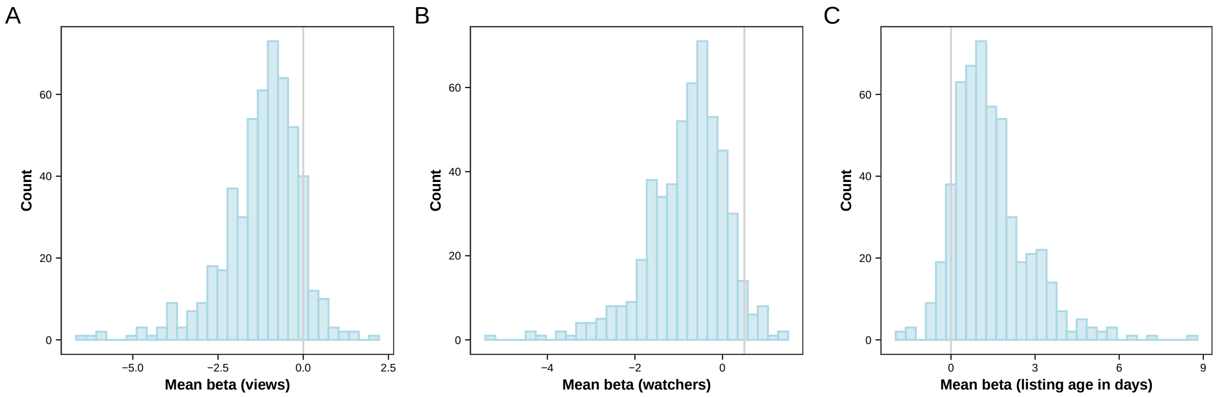

Figure S33. The effect of (A) the number of views, (B) the number of watchers, and (C) the listing age (in days), on the drift rate in Model 5. Positive effects increase the probability of accepting offers. Displayed are the distributions of mean posterior parameter estimates.

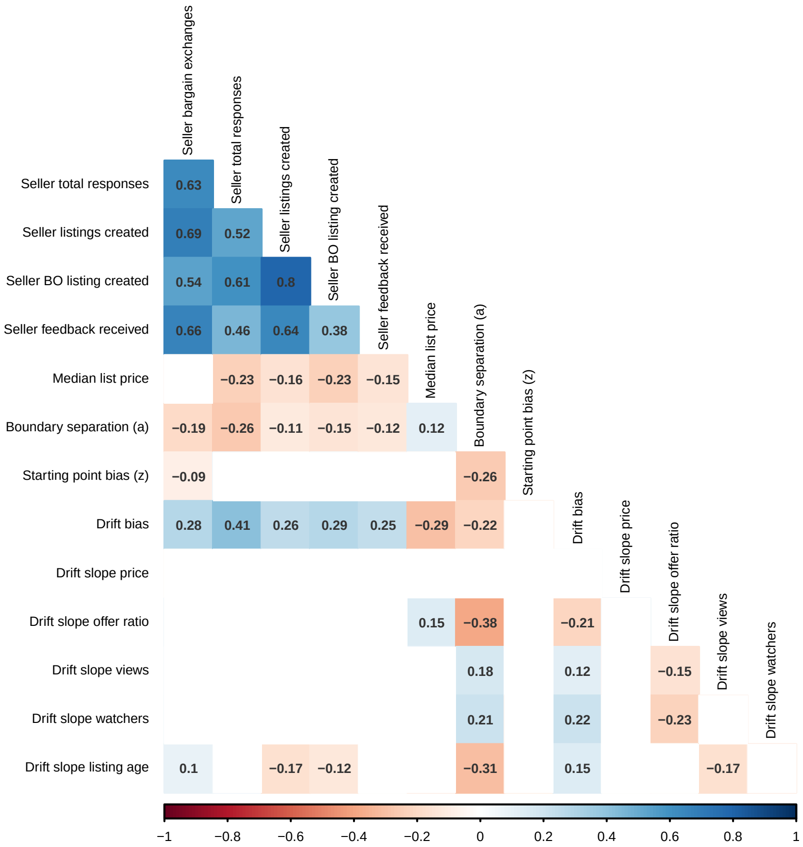

Figure S34. Correlations between mean parameter estimates from Model 5 and seller characteristics. The seller characteristics are: Seller bargain exchanges - number of previous best offer exchanges the seller has participated in; Seller total responses - seller's total number of Best-Offer responses (acceptances and rejections); Seller listings created - number of listings created by the seller dating back to 2008; Seller BO listing created - number of Best-Offer-listings created by the seller dating back to 2008; Seller feedback received - seller's amount of previous feedback received at the time of the offer. Only significant correlations ($p < 0.01$) are displayed.

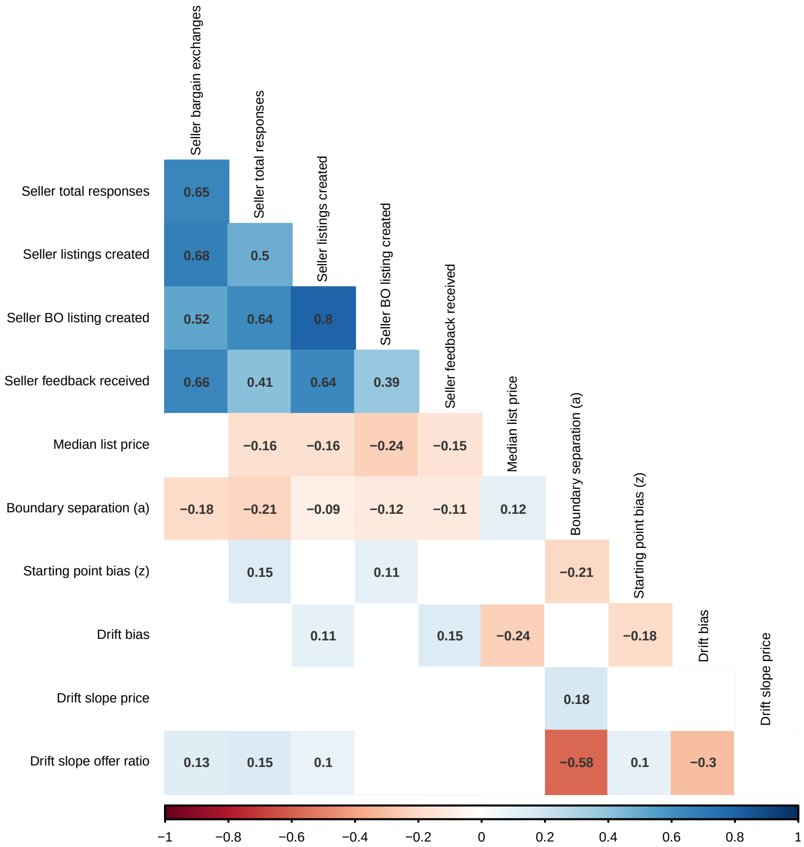

Figure S35. Correlations between mean posteriors of seller model parameters and seller experience using the best fitting model for each seller in the eBay observational data, but with counteroffers pooled with rejections. The seller characteristics are: Seller bargain exchanges - number of previous best offer exchanges the seller has participated in; Seller total responses - seller's total number of Best-Offer responses (acceptances and rejections); Seller listings created - number of listings created by the seller dating back to 2008; Seller BO listing created - number of Best-Offer-listings created by the seller dating back to 2008; Seller feedback received - seller's amount of previous feedback received at the time of the offer. Only significant correlations ($p < 0.01$) are displayed.

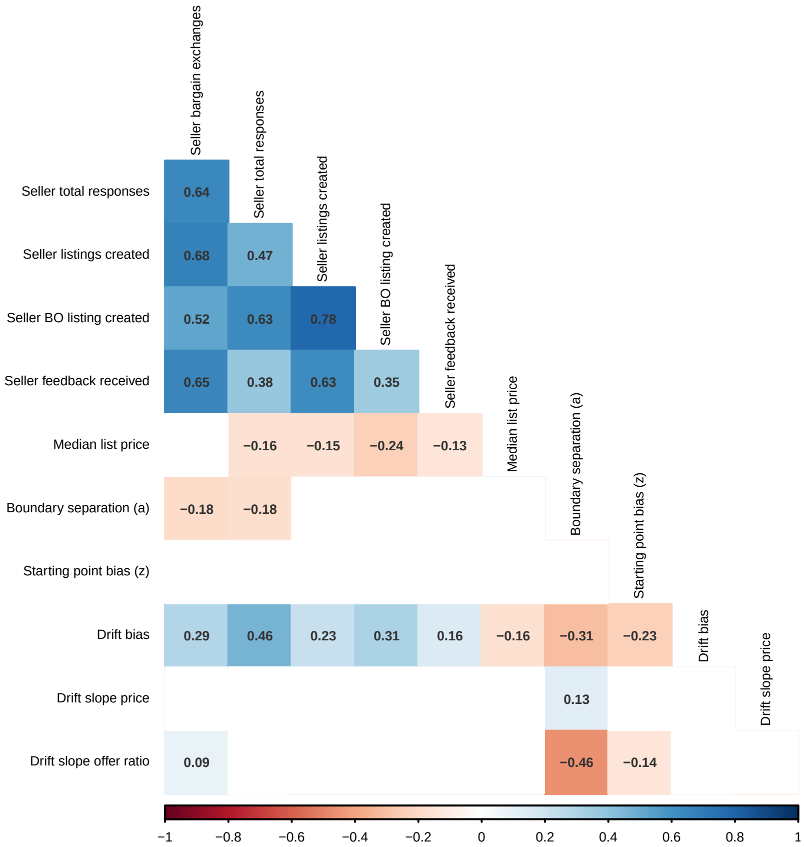

Figure S36. Correlations between mean posteriors of seller model parameters and seller experience using the best fitting model for each seller in the eBay observational data, but with counteroffers pooled with acceptances. The seller characteristics are: Seller bargain exchanges - number of previous best offer exchanges the seller has participated in; Seller total responses - seller's total number of Best-Offer responses (acceptances and rejections); Seller listings created - number of listings created by the seller dating back to 2008; Seller BO listing created - number of Best-Offer-listings created by the seller dating back to 2008; Seller feedback received - seller's amount of previous feedback received at the time of the offer. Only significant correlations ($p < 0.01$) are displayed.

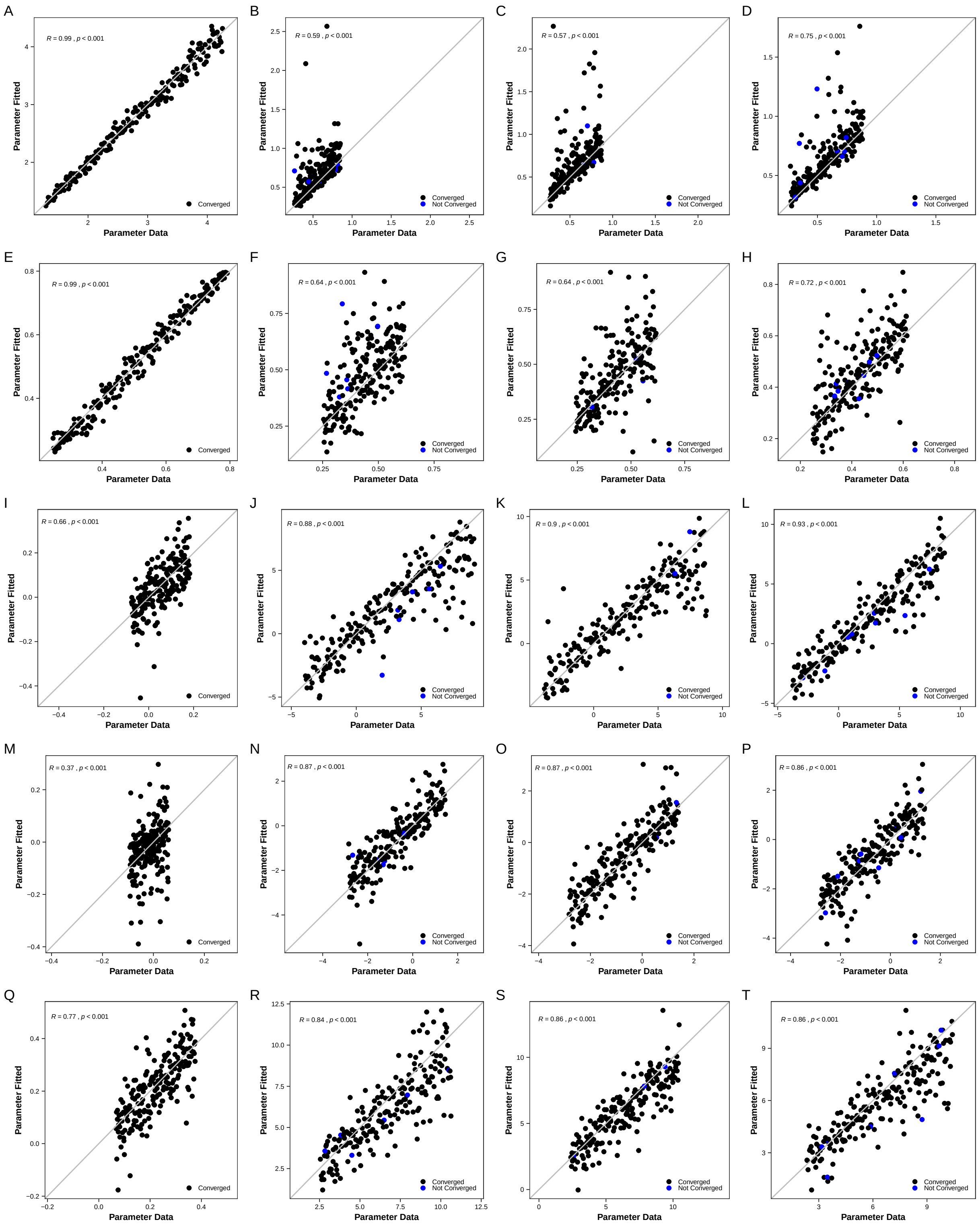
A
R = 0.99 , p < 0.001
Converged
Parameter Fitted
Parameter Data
B
R = 0.59 , p < 0.001
C
R = 0.57 , p < 0.001
D
R = 0.75 , p < 0.001
E
R = 0.99 , p < 0.001
F
R = 0.64 , p < 0.001
G
R = 0.64 , p < 0.001
H
R = 0.72 , p < 0.001
I
R = 0.66 , p < 0.001
J
R = 0.88 , p < 0.001
K
R = 0.9 , p < 0.001
L
R = 0.93 , p < 0.001
M
R = 0.37 , p < 0.001
N
R = 0.87 , p < 0.001
O
R = 0.87 , p < 0.001
P
R = 0.86 , p < 0.001
Q
R = 0.77 , p < 0.001
R
R = 0.84 , p < 0.001
S
R = 0.86 , p < 0.001
T
R = 0.86 , p < 0.001
Converged
Not Converged

Figure S37. Parameter Recovery. Correlations between simulated and fitted parameter values. (A, B, C, D) Boundary separation (a). (A) Standard DDM. (B) Gamma DDM. (C) Time of Day Gamma DDM. (D) Time of Day and Offer Ratio Gamma DDM. (E, F, G, H) Starting point bias (z). (E) Standard DDM. (F) Gamma DDM. (G) Time of Day Gamma DDM. (H) Time of Day and Offer Ratio Gamma DDM. (I, J, K, L) Drift intercept (β_0). (I) Standard DDM. (J) Gamma DDM. (K) Time of Day Gamma DDM. (L) Time of Day and Offer Ratio Gamma DDM. (M, N, O, P) Drift slope for list price (β_1). (M) Standard DDM. (N) Gamma DDM. (O) Time of Day Gamma DDM. (P) Time of Day and Offer Ratio Gamma DDM. (Q, R, S, T) Drift slope for offer ratio (β_2). (Q) Standard DDM. (R) Gamma DDM. (S) Time of Day Gamma DDM. (T) Time of Day and Offer Ratio Gamma DDM.

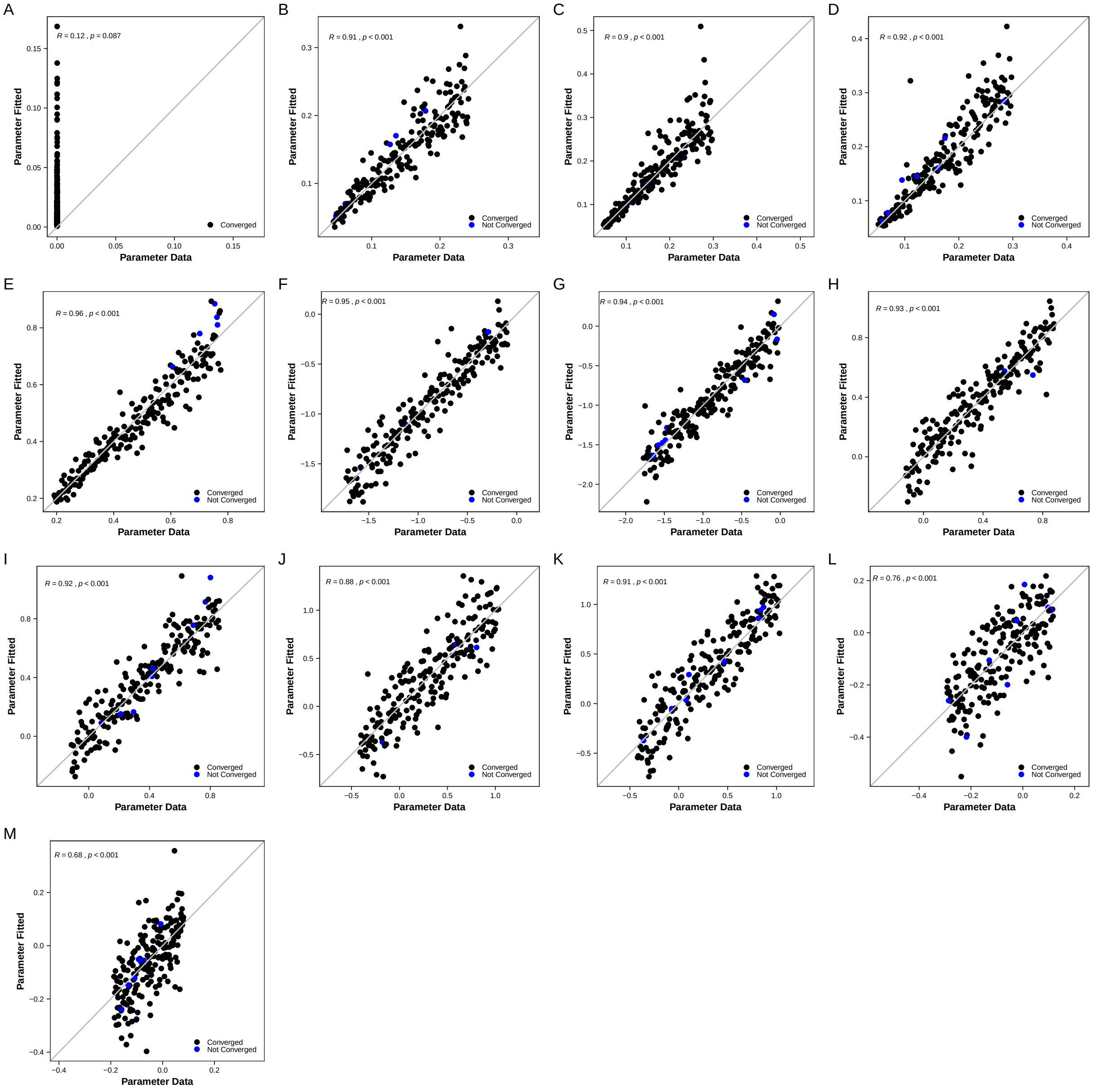

Figure S38. Parameter Recovery. Correlations between simulated and fitted parameter values for non-decision time parameters. (A) Standard DDM (t). (B, C, D) Rate parameter of Gamma distribution (β). (B) Gamma DDM. (C) Time of Day Gamma DDM. (D) Time of Day and Offer Ratio Gamma DDM. (E) Shape parameter of Gamma distribution (α) for Gamma DDM. (F, G, H, I, J, K, L, M) Parameters in sinusoidal function of the shape parameter. (F) Intercept Time of Day Gamma DDM (h_0). (G) Intercept of Time of Day and Offer Ratio Gamma DDM (h_0). (H) Linear time of day parameter of Time of Day Gamma DDM (h_1). (I) Linear time of day parameter of Time of

Day and Offer Ratio Gamma DDM (h_1). (J) Quadratic time of day parameter of Time of Day Gamma DDM (h_2). (K) Quadratic time of day parameter of Time of Day and Offer Ratio Gamma DDM (h_2). (L) Linear offer ratio parameter of Time of Day and Offer Ratio Gamma DDM (d_1). (M) Quadratic offer ratio parameter of Time of Day and Offer Ratio Gamma DDM (d_2).

Supplementary Tables

	Mean	St.dev.	Median
Listing-level data			
List price	491	40751	50
Sold Price	142	629	32
Decline price	281	5902	35
Accept price	191	21135	10
Item relisted	0.393	0.489	0
Number of views	40	111	17
Number of watchers	2	4	1
Number of photos	3	3	2
Sold through Best Offer	0.865	0.342	1
Sold price/List price	0.77	0.16	1
Bargained price/List price	0.734	0.142	1
Accept price/List price	0.826	0.117	1
Decline price/List price	0.693	0.18	1
Accept threshold present	0.203	0.402	0
Reject threshold present	0.284	0.451	0
Offer-level data			
Buyer’s first offer/List price	0.609	0.195	1
Seller’s response time (hours)	7	12	1
Seller bargain exchange	2926	6872	443
Buyer bargain exchange	132	592	26
Seller feedbacks	6242	23027	1471
Seller rating (percent)	99.646	2	99.87
Seller listings	11993	111900	1367
Seller BO listings	2465	14514	222
Messages included in offer	0.073	0.261	0
Item listing age (days)	25	53	6
Offer expired	0.069	0.254	0
Offer accepted	0.289	0.453	0
Offer rejected	0.135	0.342	0
Offer countered	0.262	0.44	0
Offer autodeclined	0.205	0.403	0
Offer autoaccepted	0.03	0.171	0
Offer rejected – another offer accepted	0.01	0.098	0

Table S1. Summary statistics for eBay observational data variables.

	logRT (hours)					
	(accept)	(accept)	(accept)	(reject)	(reject)	(reject)
	(1)	(2)	(3)	(4)	(5)	(6)
Buyer First Offer/Price (p1/p0)	-0.24^{***}	-0.23^{***}	-0.23^{***}	0.10^{***}	0.12^{***}	0.11^{***}
	(0.01)	(0.01)	(0.01)	(0.01)	(0.01)	(0.02)
Offer Create Hour		0.20^{***}	0.20^{***}		0.15^{***}	0.15^{***}
		(0.004)	(0.004)		(0.01)	(0.01)
Offer Create Hour Squared		0.46^{***}	0.46^{***}		0.41^{***}	0.41^{***}
		(0.003)	(0.003)		(0.01)	(0.01)
Price		0.03^{***}	0.03^{***}		-0.06^{***}	-0.06^{***}
		(0.01)	(0.01)		(0.01)	(0.01)
Views		0.01	0.01		0.01^{**}	0.01^{**}
		(0.01)	(0.01)		(0.005)	(0.005)
Watchers		0.16^{***}	0.15^{***}		0.01^{**}	0.01^{**}
		(0.01)	(0.01)		(0.005)	(0.005)
Item Relisted		-0.01	-0.01		0.08^{***}	0.08^{***}
		(0.01)	(0.01)		(0.01)	(0.01)
Listing Age in Days		0.02^{***}	0.02^{***}		0.12^{***}	0.11^{***}
		(0.004)	(0.004)		(0.01)	(0.01)
Photos		-0.02^{**}	-0.02^{**}		-0.03^{***}	-0.03^{***}
		(0.01)	(0.01)		(0.01)	(0.01)
Buyer Exchange Number			-0.003			-0.03^{***}
			(0.004)			(0.01)
Seller Exchange Number			-0.14^{***}			-0.01
			(0.02)			(0.03)
Seller Listings			0.002			0.01
			(0.05)			(0.01)
Seller BO Listings			0.002			-0.05^{**}
			(0.01)			(0.02)
Seller Feedbacks			0.05^{*}			0.07^{***}
			(0.02)			(0.02)
p1/p0:Seller Exchange Number			-0.003			-0.04
			(0.02)			(0.02)
Constant	0.30^{***}	-0.12^{***}	-0.16^{***}	0.42^{***}	0.02	0.02
	(0.01)	(0.01)	(0.01)	(0.01)	(0.02)	(0.02)
Observations	255,936	255,936	255,936	116,787	116,787	116,787
Log Likelihood	-531,562.50	-522,356.50	-522,346.10	-248,936.60	-245,697.90	-245,692.10
Akaike Inf. Crit.	1,063,137.00	1,044,741.00	1,044,732.00	497,885.20	491,423.80	491,424.10
Bayesian Inf. Crit.	1,063,200.00	1,044,887.00	1,044,941.00	497,943.20	491,559.20	491,617.50

Note: *p<0.05; **p<0.01; ***p<0.001

Table S2. Linear regressions for eBay observational data of log(RT) on first buyer offer ratio (p_1/p_0) (z-score) conditional on the seller accepting, or rejecting the offers. These regressions are restricted to the range $p_1/p_0 = [0.36, 0.68]$. These regressions include random effects (clustered by seller) on the intercept and offer ratio. All variables are z-scored.

	logRT (hours)		
	(reject)	(reject)	(reject)
	(1)	(2)	(3)
Buyer First Offer/Price (p1/p0)	0.21***	0.23***	0.23***
	(0.01)	(0.01)	(0.01)
Offer Create Hour		0.10***	0.10***
		(0.005)	(0.005)
Offer Create Hour Squared		0.28***	0.28***
		(0.004)	(0.004)
Price		−0.10***	−0.10***
		(0.01)	(0.01)
Views		0.02***	0.02***
		(0.004)	(0.004)
Watchers		−0.02***	−0.02***
		(0.004)	(0.004)
Item Relisted		0.26***	0.26***
		(0.01)	(0.01)
Listing Age in Days		0.23***	0.23***
		(0.01)	(0.01)
Photos		−0.07***	−0.07***
		(0.01)	(0.01)
Buyer Exchange Number			−0.04***
			(0.01)
Seller Exchange Number			−0.08***
			(0.02)
Seller Listings			0.01
			(0.01)
Seller BO Listings			−0.01
			(0.01)
Seller Feedbacks			0.01
			(0.02)
p1/p0:Seller Exchange Number			−0.04
			(0.02)
Constant	1.74***	1.45***	1.43***
	(0.01)	(0.01)	(0.02)
Observations	183,072	183,072	183,072
Log Likelihood	-399,243.00	-396,114.20	-395,834.60
Akaike Inf. Crit.	798,494.00	792,252.50	791,709.20
Bayesian Inf. Crit.	798,534.50	792,373.90	791,911.50
Note:	*p<0.05; **p<0.01; ***p<0.001		

Table S3. Linear regressions for eBay observational data of log(RT) on first buyer offer ratio (p_1/p_0) (z-score) conditional on the seller rejecting or letting the offer expire. These regressions are restricted to the range $p_1/p_0 = [0.36, 0.68]$. These regressions include random effects (clustered by seller) on the intercept for (1) and (2) and intercept and offer ratio for (3). Expired offers are considered rejection with a 48 hour response time. All

variables are z-scored.

	logRT (hours)		
	(counter)	(counter)	(counter)
	(1)	(2)	(3)
Buyer First Offer/Price (p1/p0)	-0.06^{***}	-0.03^{***}	-0.03^{***}
	(0.01)	(0.01)	(0.01)
Seller Counteroffer/Price	-0.10^{***}	-0.11^{***}	-0.11^{***}
	(0.01)	(0.01)	(0.01)
Offer Create Hour		0.15^{***}	0.15^{***}
		(0.005)	(0.005)
Offer Create Hour Squared		0.44^{***}	0.44^{***}
		(0.004)	(0.004)
Price		-0.03^{***}	-0.03^{***}
		(0.01)	(0.01)
Views		0.003	0.003
		(0.005)	(0.005)
Watchers		0.09^{***}	0.09^{***}
		(0.01)	(0.01)
Item Relisted		0.07^{***}	0.07^{***}
		(0.01)	(0.01)
Listing Age in Days		0.07^{***}	0.07^{***}
		(0.01)	(0.01)
Photos		-0.02^{**}	-0.02^{**}
		(0.01)	(0.01)
Buyer Exchange Number			-0.02^{***}
			(0.004)
Seller Exchange Number			-0.11^{***}
			(0.02)
Seller Listings			0.12^{**}
			(0.04)
Seller BO Listings			-0.02
			(0.01)
Seller Feedbacks			0.17^{***}
			(0.02)
p1/p0:Seller Exchange Number			-0.03^{***}
			(0.01)
Constant	0.06^{***}	-0.37^{***}	-0.36^{***}
	(0.01)	(0.01)	(0.01)
Observations	187,740	187,740	187,740
Log Likelihood	-395,095.60	-389,353.70	-389,308.40
Akaike Inf. Crit.	790,201.30	778,733.50	778,654.80
Bayesian Inf. Crit.	790,252.00	778,865.30	778,847.60
Note:			*p<0.05; **p<0.01; ***p<0.001

Table S4. Linear regressions for eBay observational data of log(RT) on first buyer offer ratio (p_1/p_0) **(z-score) conditional on the seller countering the offer.** These regressions are restricted to the range $p_1/p_0 =$

$[0.36, 0.68]$. These regressions include random effects (clustered by seller) on the intercept and offer ratio. All variables are z-scored.

	P (accept)	P (accept)	P (accept)
	(1)	(2)	(3)
Buyer First Offer/Price (p1/p0)	2.13***	2.04***	1.97***
	(0.01)	(0.01)	(0.01)
Price		−0.28***	−0.28***
		(0.01)	(0.01)
Views		−0.26***	−0.26***
		(0.01)	(0.01)
Watchers		−0.40***	−0.40***
		(0.01)	(0.01)
Item Relisted		0.29***	0.29***
		(0.01)	(0.01)
Listing Age in Days		0.39***	0.37***
		(0.01)	(0.01)
Photos		0.08***	0.08***
		(0.01)	(0.01)
Buyer Exchange Number			−0.05***
			(0.004)
Seller Exchange Number			0.52***
			(0.02)
Seller Listings			−0.01
			(0.01)
Seller BO Listings			−0.07***
			(0.01)
Seller Feedbacks			−0.01
			(0.03)
p1/p0:Seller Exchange Number			−0.16***
			(0.02)
Constant	1.12***	1.20***	1.42***
	(0.01)	(0.01)	(0.02)
Observations	869,395	869,395	869,395
Log Likelihood	-272,164.40	-260,517.90	-260,010.40
Akaike Inf. Crit.	544,338.80	521,057.90	520,054.90
Bayesian Inf. Crit.	544,397.20	521,186.30	520,253.40
Note:			*p<0.05; **p<0.01; ***p<0.001

Table S5. Logistic regressions for eBay observational data of acceptance versus rejection of first buyer offer ratio (p_1/p_0) **(z-score)**. These regressions include random effects (clustered by seller) on the intercept and offer ratio. All variables are z-scored.

	logRT (hours)					
	(accept)	(accept)	(accept)	(reject)	(reject)	(reject)
	(1)	(2)	(3)	(4)	(5)	(6)
Buyer Second Offer/Price (p2/p0)	-0.23^{***}	-0.22^{***}	-0.22^{***}	0.11^{***}	0.12^{***}	0.12^{***}
	(0.03)	(0.03)	(0.03)	(0.02)	(0.02)	(0.02)
Offer Create Hour		0.12^{***}	0.12^{***}		0.01	0.01
		(0.02)	(0.02)		(0.02)	(0.02)
Offer Create Hour Squared		0.35^{***}	0.35^{***}		0.28^{***}	0.28^{***}
		(0.02)	(0.02)		(0.01)	(0.01)
Price		0.001	-0.002		-0.06^{**}	-0.05^{**}
		(0.03)	(0.03)		(0.02)	(0.02)
Views		0.05	0.05		0.01	0.01
		(0.05)	(0.05)		(0.01)	(0.01)
Watchers		0.16^{***}	0.16^{***}		-0.01	-0.01
		(0.04)	(0.04)		(0.02)	(0.02)
Item Relisted		0.11^{*}	0.11^{*}		-0.04	-0.03
		(0.04)	(0.04)		(0.04)	(0.04)
Listing Age in Days		0.07^{***}	0.07^{***}		0.11^{***}	0.11^{***}
		(0.02)	(0.02)		(0.02)	(0.02)
Photos		-0.07^{**}	-0.07^{**}		-0.06^{**}	-0.06^{**}
		(0.03)	(0.03)		(0.02)	(0.02)
Buyer Exchange Number			0.02			-0.02
			(0.06)			(0.01)
Seller Exchange Number			-0.03			-0.001
			(0.05)			(0.01)
Seller Listings			0.27			0.0004
			(0.16)			(0.02)
Seller BO Listings			-0.05			-0.08^{**}
			(0.03)			(0.03)
Seller Feedbacks			0.04			0.13^{***}
			(0.04)			(0.04)
p2/p0:Seller Exchange Number			0.04			-0.002
			(0.04)			(0.02)
Constant	0.31^{***}	-0.03	-0.03	-0.03	-0.27^{***}	-0.27^{***}
	(0.03)	(0.04)	(0.04)	(0.03)	(0.03)	(0.04)
Observations	11,667	11,667	11,667	17,346	17,346	17,346
Log Likelihood	-24,936.59	-24,732.11	-24,742.15	-38,292.71	-38,099.42	-38,107.38
Akaike Inf. Crit.	49,885.18	49,492.23	49,524.29	76,597.42	76,226.84	76,254.77
Bayesian Inf. Crit.	49,929.37	49,595.33	49,671.58	76,643.99	76,335.50	76,409.99
Note:						$^{*}p<0.05$; $^{**}p<0.01$; $^{***}p<0.001$

Table S6. Linear regressions for eBay observational data of log(RT) on second buyer offer ratio (p_2/p_0) (z-score) conditional on the seller accepting, or rejecting the offers. These regressions include random effects (clustered by seller) on the intercept and offer ratio. All variables are z-scored.

	logRT (hours)					
	(accept)	(accept)	(accept)	(reject)	(reject)	(reject)
	(1)	(2)	(3)	(4)	(5)	(6)
Buyer Third Offer/Price (p3/p0)	-0.29^{**}	-0.27^{**}	-0.26^{**}	0.09	0.09	0.09
	(0.09)	(0.10)	(0.10)	(0.05)	(0.05)	(0.05)
Offer Create Hour		0.08	0.08		-0.01	-0.01
		(0.07)	(0.07)		(0.05)	(0.05)
Offer Create Hour Squared		0.36^{***}	0.36^{***}		0.25^{***}	0.25^{***}
		(0.06)	(0.06)		(0.04)	(0.04)
Price		0.01	0.01		0.003	-0.003
		(0.09)	(0.09)		(0.05)	(0.05)
Views		-0.01	-0.03		-0.001	0.001
		(0.15)	(0.15)		(0.04)	(0.04)
Watchers		0.03	0.04		0.03	0.03
		(0.13)	(0.13)		(0.05)	(0.05)
Item Relisted		-0.05	-0.06		-0.16	-0.16
		(0.14)	(0.14)		(0.11)	(0.11)
Listing Age in Days		0.005	0.003		0.22^{***}	0.21^{***}
		(0.07)	(0.07)		(0.05)	(0.05)
Photos		-0.12	-0.11		-0.13^{*}	-0.12^{*}
		(0.08)	(0.08)		(0.06)	(0.06)
Buyer Exchange Number			0.05			
			(0.03)			
Seller Feedbacks			0.02			0.20^{**}
			(0.14)			(0.07)
Seller Listings			0.97			-0.01
			(0.73)			(0.04)
Seller BO Listings			-0.05			0.08
			(0.08)			(0.06)
Constant	0.13	-0.22	-0.19	-0.33^{***}	-0.51^{***}	-0.47^{***}
	(0.09)	(0.12)	(0.13)	(0.06)	(0.09)	(0.09)
Observations	1,204	1,204	1,204	2,211	2,211	2,211
Log Likelihood	-2,635.86	-2,626.74	-2,629.29	-4,991.67	-4,976.35	-4,976.07
Akaike Inf. Crit.	5,279.72	5,277.48	5,290.58	9,991.34	9,976.70	9,982.14
Bayesian Inf. Crit.	5,300.10	5,338.60	5,372.08	10,014.14	10,045.12	10,067.66
Note:						*p<0.05; **p<0.01; ***p<0.001

Table S7. Linear regressions for eBay observational data of log(RT) on third buyer offer ratio (p_3/p_0) (z-score) conditional on the seller accepting, or rejecting the offers. These regressions include random effects (clustered by seller) on the intercept and offer ratio. All variables are z-scored.

	logRT (hours)					
	(accept)	(accept)	(accept)	(reject)	(reject)	(reject)
	(1)	(2)	(3)	(4)	(5)	(6)
Seller compromise (p0-p1s)/(p0-p1b)	-0.13^{***}	-0.12^{***}	-0.12^{***}	0.18^{***}	0.18^{***}	0.18^{***}
	(0.01)	(0.01)	(0.01)	(0.01)	(0.01)	(0.01)
Offer Create Hour		-0.04^{***}	-0.04^{***}		-0.06^{***}	-0.06^{***}
		(0.01)	(0.01)		(0.01)	(0.01)
Offer Create Hour Squared		0.31^{***}	0.31^{***}		0.25^{***}	0.25^{***}
		(0.01)	(0.01)		(0.01)	(0.01)
Price		-0.04^{***}	-0.04^{***}		-0.03^{***}	-0.03^{***}
		(0.01)	(0.01)		(0.01)	(0.01)
Views		0.04^{*}	0.04^{*}		-0.02^{*}	-0.02^{*}
		(0.02)	(0.02)		(0.01)	(0.01)
Watchers		0.25^{***}	0.25^{***}		0.08^{***}	0.08^{***}
		(0.01)	(0.01)		(0.01)	(0.01)
Item Relisted		0.15^{***}	0.15^{***}		-0.10^{***}	-0.10^{***}
		(0.02)	(0.02)		(0.02)	(0.02)
Listing Age in Days		0.10^{***}	0.10^{***}		-0.03^{***}	-0.03^{***}
		(0.01)	(0.01)		(0.01)	(0.01)
Photos		-0.02^{*}	-0.02^{*}		0.01	0.01
		(0.01)	(0.01)		(0.01)	(0.01)
Buyer Exchange Number			-0.02			-0.01
			(0.03)			(0.01)
(p0-p1s)/(p0-p1b):Exchange			0.02			-0.002
			(0.04)			(0.02)
Constant	0.57^{***}	0.25^{***}	0.25^{***}	1.06^{***}	0.83^{***}	0.83^{***}
	(0.01)	(0.01)	(0.01)	(0.01)	(0.01)	(0.01)
Observations	81,793	81,793	81,793	76,591	76,591	76,591
Log Likelihood	-178,137.00	-176,720.40	-176,725.50	-167,897.40	-167,204.40	-167,210.70
Akaike Inf. Crit.	356,281.90	353,464.70	353,478.90	335,802.70	334,432.80	334,449.50
Bayesian Inf. Crit.	356,319.20	353,576.50	353,609.30	335,839.70	334,543.70	334,578.90
Note:	$^{*}p<0.05$; $^{**}p<0.01$; $^{***}p<0.001$					

Table S8. Linear regressions for eBay observational data of buyers' log(RT) on seller's compromise conditional on the buyer accepting, or rejecting the offers. The seller's compromise is the amount that they lowered their counteroffer, divided by the gap between the list price and the buyer's offer. A 100% compromise would be a counteroffer that matches the buyer's offer; a 0% compromise would be a counteroffer that is the list price. These regressions include random effects (clustered by buyer) on the intercept. All variables are z-scored.

	P (buyer second offer exists)		
	(1)	(2)	(3)
Buyer first offer/Price	-0.04^{***}	-0.04^{***}	-0.04^{***}
	(0.004)	(0.004)	(0.004)
Buyer first offer/Price Squared	-0.17^{***}	-0.16^{***}	-0.17^{***}
	(0.003)	(0.003)	(0.003)
Rejection RT (hours)			-0.10^{***}
			(0.004)
Time Offer Response Hour		0.001	
		(0.003)	
Time Offer Hour Response Squared		-0.01^{**}	
		(0.003)	
Price		-0.05^{***}	
		(0.004)	
Buyer Exchange Number		-0.08^{***}	
		(0.01)	
Seller Exchange Number		0.03^{***}	
		(0.003)	
Seller Feedbacks		-0.01^{***}	
		(0.004)	
Listing Age in Days		0.02^{***}	
		(0.003)	
Constant	-0.96^{***}	-0.96^{***}	-0.97^{***}
	(0.004)	(0.01)	(0.004)
Observations	226,653	226,653	226,653
Log Likelihood	-53,084.50	-51,874.45	-51,838.82
ρ	-0.54*** (0.01)	-0.49*** (0.02)	-0.49*** (0.02)
Note:			$^{*}p<0.05$; $^{**}p<0.01$; $^{***}p<0.001$

Table S9. Probit regression for eBay observational data for existence of buyers' second offer. All variables are z-scored. (1), (2) are the first stage of the Heckman correction.

	Buyer second offer/Price	
	(1)	(2)
Rejection RT (hours)	0.003***	0.003***
	(0.001)	(0.001)
Buyer first offer/Price	0.19***	0.19***
	(0.001)	(0.001)
Price		−0.02***
		(0.001)
Views		−0.003***
		(0.0005)
Watchers		−0.002***
		(0.001)
Photos		0.0004
		(0.0005)
Listing Age in Days		−0.001
		(0.0004)
Buyer Exchange Number		−0.01***
		(0.001)
Constant	0.70***	0.69***
	(0.003)	(0.003)
Observations	226,653	226,653
Log Likelihood	-53,084.50	-51,874.45
ρ	-0.54*** (0.01)	-0.49*** (0.02)
Note:	*p<0.05; **p<0.01; ***p<0.001	

Table S10. Heckman correction regression second stage for eBay observational data for buyers' second offer amount as a fraction of list price (p_2/p_0). All variables are z-scored.

	P(buyer second offer accepted)	
	(1)	(2)
Rejection RT (hours)	0.13***	0.12***
	(0.02)	(0.02)
Buyer Second Offer/Price (p2/p0)	1.00***	1.08***
	(0.02)	(0.02)
Price		−0.49***
		(0.02)
Buyer Exchange Number		0.01
		(0.02)
Photos		0.17***
		(0.02)
Views		−0.60***
		(0.04)
Listing Age in Days		0.20***
		(0.02)
Watchers		−0.55***
		(0.03)
Constant	−0.51***	−0.74***
	(0.02)	(0.02)
Observations	23,435	23,435
Log Likelihood	-13,543.06	-12,279.99
Akaike Inf. Crit.	27,092.13	24,577.98
Note:	*p<0.05; **p<0.01; ***p<0.001	

Table S11. Logistic regression for eBay observational data for probability of seller accepting buyer's second offer. All variables are z-scored.

	logRT (hours)					
	(accept)	(accept)	(accept)	(reject)	(reject)	(reject)
	(1)	(2)	(3)	(4)	(5)	(6)
Buyer First Offer/Price (p1/p0)	-0.12^{***}	-0.11^{***}	-0.11^{***}	-0.0001	0.03^{**}	0.02^{*}
	(0.01)	(0.01)	(0.01)	(0.01)	(0.01)	(0.01)
Offer Create Hour		0.19^{***}	0.18^{***}		0.15^{***}	0.15^{***}
		(0.003)	(0.003)		(0.004)	(0.004)
Offer Create Hour Squared		0.45^{***}	0.45^{***}		0.43^{***}	0.43^{***}
		(0.003)	(0.003)		(0.003)	(0.003)
Price		-0.0004	0.0000		-0.04^{***}	-0.04^{***}
		(0.004)	(0.004)		(0.004)	(0.004)
Views		0.002	0.002		0.01^{**}	0.01^{**}
		(0.004)	(0.004)		(0.004)	(0.004)
Watchers		0.09^{***}	0.09^{***}		0.03^{***}	0.03^{***}
		(0.004)	(0.004)		(0.004)	(0.004)
Item Relisted		0.03^{***}	0.03^{***}		0.08^{***}	0.08^{***}
		(0.01)	(0.01)		(0.01)	(0.01)
Listing Age in Days		0.04^{***}	0.04^{***}		0.09^{***}	0.09^{***}
		(0.003)	(0.003)		(0.005)	(0.005)
Photos		-0.01^{*}	-0.01		-0.02^{***}	-0.02^{***}
		(0.004)	(0.004)		(0.01)	(0.01)
Buyer Exchange Number			-0.01^{***}			-0.03^{***}
			(0.003)			(0.004)
Seller Exchange Number			-0.13^{***}			-0.06^{***}
			(0.02)			(0.02)
Seller Listings			0.06			0.01
			(0.04)			(0.01)
Seller BO Listings			-0.01			-0.004
			(0.01)			(0.01)
Seller Feedbacks			0.08^{***}			0.09^{***}
			(0.02)			(0.02)
p1/p0:Seller Exchange Number			-0.02			-0.03^{*}
			(0.01)			(0.01)
Constant	0.25^{***}	-0.20^{***}	-0.23^{***}	0.25^{***}	-0.17^{***}	-0.17^{***}
	(0.01)	(0.01)	(0.01)	(0.01)	(0.01)	(0.01)
Observations	413,609	413,609	413,609	274,460	274,460	274,460
Log Likelihood	-859,399.10	-845,408.80	-845,378.00	-577,747.80	-569,602.30	-569,583.20
Akaike Inf. Crit.	1,718,810.00	1,690,846.00	1,690,796.00	1,155,508.00	1,139,233.00	1,139,206.00
Bayesian Inf. Crit.	1,718,876.00	1,690,999.00	1,691,015.00	1,155,571.00	1,139,380.00	1,139,417.00
Note:						*p<0.05; **p<0.01; ***p<0.001

Table S12. Linear regressions for eBay observational data of log(RT) on first buyer offer ratio (p_1/p_0) (z-score) conditional on the seller response with counteroffers pooled with acceptances or rejections. Linear regressions for eBay observational data of log(RT) on first buyer offer ratio (p_1/p_0) (z- score) conditional on the seller accepting or countering, or rejecting or countering the offers. These regressions are restricted to the range

$p_1/p_0 = [0.36, 0.68]$. These regressions include random effects (clustered by seller) on the intercept and offer ratio. All variables are z-scored.

	Mean	St.dev.	Median
Offer Ratio	0.6	0.24	1
Price (dollars)	15	4	15
Sale Price (Offer Accepted or Auto-accepted)	0.955	1	1
Bargained Price (Offer Accepted)	0.806	0.138	1
Accept Price Ratio	0.9	0	1
Decline Price Ratio	0.375	0.106	0
Response Time (hour)	1	2	0
Year since Registration	18	6	21
Viable Items	36	21	28
Seller Feedbacks	9644	25588	2746
Number of Best Offers	0.46	0.754	0
Item Relisted	0.667	0.472	1
Offer Expired	0.036	0.188	0
Offer Accepted	0.441	0.497	0
Offer Declined	0.144	0.351	0
Offer Countered	0.311	0.464	0
Offer Auto-declined	0.062	0.241	0
Offer Auto-accepted	0.005	0.074	0

Table S13. Summary statistics of variables of interest for eBay field experiment 1.

	Mean	St.dev.	Median
Offer Ratio	0.45	0.244	0
Price (dollars)	13	2	13
Sale Price (Offer Accepted or Auto-accepted)	0.734	1	1
Bargained Price (Offer Accepted)	0.629	0.172	1
Accept Price Ratio	0.764	0.055	1
Decline Price Ratio	0.243	0.133	0
Response Time (hour)	3	7	1
Year since Registration	14	8	14
Viable Items	56	67	36
Seller Feedbacks	8876	20191	3028
Number of Best Offers	0.33	0.94	0
Item Relisted	0.433	0.496	0
Offer Expired	0.066	0.248	0
Offer Accepted	0.431	0.495	0
Offer Declined	0.186	0.389	0
Offer Countered	0.254	0.435	0
Offer Auto-declined	0.06	0.237	0
Offer Auto-accepted	0.004	0.06	0

Table S14. Summary statistics of variables of interest for eBay field experiment 2.

	P(accept)	P(accept)	P(accept)	P(accept)	P(accept)	P(accept)	P(accept)	P(accept)	P(accept)
	(1)	(2)	(3)	(4)	(5)	(6)	(7)	(8)	(9)
Offer/Price (p1/p0)	3.55***	4.90***	6.02***	2.63***	2.76***	2.80***	2.64***	2.83***	2.88***
	(0.44)	(1.05)	(1.58)	(0.12)	(0.13)	(0.13)	(0.11)	(0.12)	(0.13)
Number of Best Offers		−4.45***	−5.81***		−0.86***	−0.88***		−1.11***	−1.10***
		(0.94)	(1.65)		(0.12)	(0.12)		(0.12)	(0.12)
Item Relisted		1.24	2.63		−0.35*	−0.22		−0.41*	−0.21
		(1.07)	(1.61)		(0.17)	(0.18)		(0.16)	(0.17)
Feedbacks		−0.34	−0.78		0.14	0.17*		−0.02	0.02
		(0.43)	(0.61)		(0.08)	(0.09)		(0.08)	(0.08)
Years since Registration			0.41			−0.08			0.02
			(2.38)			(0.11)			(0.14)
Viable Items			−1.24			−0.03			−0.15
			(0.96)			(0.12)			(0.11)
Price			−0.99			0.03			0.12
			(0.73)			(0.17)			(0.17)
p1/p0:Feedbacks			−0.58			−0.02			0.03
			(1.89)			(0.12)			(0.15)
p1/p0:Years			−0.46			0.22			0.15
			(0.90)			(0.12)			(0.12)
p1/p0:Viable Items			−2.20			0.21			0.24
			(1.23)			(0.21)			(0.20)
Constant	1.91***	4.47***	5.39***	1.53***	1.12***	1.04***	1.53***	2.19***	2.11***
	(0.36)	(1.08)	(1.59)	(0.10)	(0.15)	(0.16)	(0.09)	(0.15)	(0.15)
Observations	321	319	308	1,873	1,858	1,844	2,194	2,177	2,152
Log Likelihood	-52.28	-20.55	-16.25	-507.16	-472.23	-465.06	-590.15	-526.22	-513.77
Akaike Inf. Crit.	108.56	51.11	54.51	1,018.32	954.46	952.12	1,184.30	1,062.45	1,049.54

Note: *p<0.05; **p<0.01; ***p<0.001

Table S15. Logistic regressions for eBay field experiments 1 and 2 of acceptance versus rejection of first buyer offer ratio (p_1/p_0) (z-score). (1), (2), (3) Experiment 1. (4), (5), (6) Experiment 2. (7), (8), (9) Experiments 1 and 2. All variables are z-scored except Number of Best Offer.

	logRT (hours)		
	(all)	(all)	(all)
	(1)	(2)	(3)
Offer/Price	-0.20	-0.25^{***}	-0.23^{***}
	(0.21)	(0.07)	(0.07)
Rejected	1.34^{*}	0.76^{***}	0.72^{***}
	(0.60)	(0.17)	(0.16)
Offer/Price:Rejected	1.11^{*}	0.28^{*}	0.25
	(0.50)	(0.14)	(0.13)
Constant	-1.10^{***}	-0.93^{***}	-0.97^{***}
	(0.27)	(0.12)	(0.11)
Observations	321	1,873	2,194
Log Likelihood	-663.72	-3,555.83	-4,231.55
Akaike Inf. Crit.	1,339.43	7,133.66	8,485.11
Bayesian Inf. Crit.	1,362.06	7,194.54	8,547.74
Note:	*p<0.05; **p<0.01; ***p<0.001		

Table S16. Linear regressions for eBay field experiments 1 and 2 for log RT (in hours) for all types of seller responses. (1) Regression includes random effects (clustered by seller) on the intercept. (2), (3) Regressions include random effects (clustered by seller) on the intercept and offer ratio and type of seller response. The random effects structure was chosen based on the model with the lowest AIC from a model comparison. The first offer as a fraction of list price (p_1/p_0) is z-scored.

	logRT (hours)					
	(accept)	(accept)	(accept)	(reject)	(reject)	(reject)
	(1)	(2)	(3)	(4)	(5)	(6)
Offer/Price (p1/p0)	−0.19	−0.16	−0.21	0.84	0.79	0.90
	(0.21)	(0.22)	(0.23)	(0.45)	(0.47)	(0.92)
Number of Best Offers		0.05	0.08		−0.01	0.03
		(0.35)	(0.35)		(0.40)	(0.44)
Item Relisted		0.44	0.60		0.43	0.52
		(0.42)	(0.42)		(0.53)	(0.59)
Price		−0.07	−0.11		−0.14	−0.17
		(0.14)	(0.15)		(0.22)	(0.25)
Feedbacks			0.24			−1.10
			(0.44)			(4.92)
Years since Registration			0.30			−0.34
			(0.27)			(1.22)
Viable Items			−0.31			0.15
			(0.27)			(1.03)
p1/p0:Feedbacks			0.23			−1.09
			(0.38)			(3.72)
p1/p0:Years			−0.24			−0.16
			(0.21)			(0.97)
p1/p0:Viable Items			0.11			0.16
			(0.22)			(0.84)
Constant	−1.10***	−1.43***	−1.52***	0.27	−0.08	−0.14
	(0.28)	(0.41)	(0.41)	(0.56)	(0.82)	(1.38)
Observations	242	242	237	79	77	71
Log Likelihood	-509.61	-510.04	-498.42	-158.83	-153.95	-138.15
Akaike Inf. Crit.	1,027.21	1,034.08	1,022.84	325.65	321.89	302.30
Bayesian Inf. Crit.	1,041.17	1,058.50	1,067.92	335.13	338.30	331.72
Note:				*p<0.05; **p<0.01; ***p<0.001		

Table S17. Linear regressions for eBay field experiment 1 of log(RT) on first buyer offer ratio (p_1/p_0) **(z-score) conditional on the seller accepting, or rejecting the offers.** These regressions include random effects (clustered by seller) on the intercept. The random effects structure was chosen based on the model with the lowest AIC from a model comparison. All variables are z-scored except Number of Best Offer.

	logRT (hours)					
	(accept)	(accept)	(accept)	(reject)	(reject)	(reject)
	(1)	(2)	(3)	(4)	(5)	(6)
Offer/Price (p1/p0)	-0.26^{***}	-0.25^{***}	-0.26^{***}	0.12	0.13	0.13
	(0.07)	(0.07)	(0.07)	(0.11)	(0.11)	(0.11)
Number of Best Offers		-0.16	-0.15		-0.02	-0.02
		(0.08)	(0.08)		(0.04)	(0.04)
Item Relisted		0.10	0.03		0.25	0.25
		(0.16)	(0.16)		(0.14)	(0.15)
Feedbacks			0.02			-0.15
			(0.13)			(0.18)
Years since Registration			0.31^{*}			-0.06
			(0.14)			(0.17)
Viable Items			-0.01			0.13
			(0.16)			(0.19)
p1/p0:Feedbacks			0.05			-0.11
			(0.07)			(0.13)
p1/p0:Years			-0.18^{*}			-0.08
			(0.08)			(0.12)
p1/p0:Viable Items			0.15			0.01
			(0.11)			(0.14)
Constant	-0.91^{***}	-1.09^{***}	-1.03^{***}	-0.03	-0.12	-0.12
	(0.13)	(0.16)	(0.16)	(0.15)	(0.17)	(0.18)
Observations	1,308	1,298	1,286	565	560	558
Log Likelihood	-2,608.27	-2,589.82	-2,569.63	-927.14	-921.76	-924.37
Akaike Inf. Crit.	5,228.55	5,195.64	5,167.27	1,866.29	1,859.51	1,876.75
Bayesian Inf. Crit.	5,259.61	5,236.99	5,239.50	1,892.31	1,894.14	1,937.29
Note:			*p<0.05; **p<0.01; ***p<0.001			

Table S18. Linear regressions for eBay field experiment 2 of log(RT) on first buyer offer ratio (p_1/p_0) (z-score) conditional on the seller accepting, or rejecting the offers. These regressions include random effects (clustered by seller) on the intercept and offer ratio. The random effects structure was chosen based on the model with the lowest AIC from a model comparison. All variables are z-scored except Number of Best Offer.

	logRT (hours)					
	(accept)	(accept)	(accept)	(reject)	(reject)	(reject)
	(1)	(2)	(3)	(4)	(5)	(6)
Offer/Price (p1/p0)	-0.24^{***}	-0.24^{**}	-0.26^{***}	0.11	0.11	0.12
	(0.07)	(0.07)	(0.07)	(0.10)	(0.11)	(0.11)
Number of Best Offers		-0.15	-0.14		-0.03	-0.02
		(0.08)	(0.08)		(0.05)	(0.05)
Item Relisted		0.15	0.09		0.17	0.19
		(0.15)	(0.15)		(0.14)	(0.15)
Feedbacks			0.08			-0.02
			(0.12)			(0.18)
Years since Registration			0.29^{*}			-0.16
			(0.12)			(0.16)
Viable Items			-0.02			0.11
			(0.14)			(0.19)
p1/p0:Feedbacks			0.10			-0.07
			(0.08)			(0.14)
p1/p0:Years			-0.19^{*}			-0.09
			(0.08)			(0.12)
p1/p0:Viable Items			0.12			-0.02
			(0.11)			(0.14)
Constant	-0.95^{***}	-0.99^{***}	-0.93^{***}	-0.14	-0.20	-0.22
	(0.11)	(0.13)	(0.14)	(0.14)	(0.17)	(0.18)
Observations	1,550	1,540	1,523	644	637	629
Log Likelihood	-3,120.71	-3,102.28	-3,070.76	-1,102.36	-1,091.27	-1,082.53
Akaike Inf. Crit.	6,253.43	6,220.56	6,169.51	2,216.72	2,198.54	2,193.07
Bayesian Inf. Crit.	6,285.50	6,263.28	6,244.11	2,243.53	2,234.20	2,255.29
Note:						*p<0.05; **p<0.01; ***p<0.001

Table S19. Linear regressions for eBay field experiments 1 and 2 of log(RT) on first buyer offer ratio (p_1/p_0) (z-score) conditional on the seller accepting, or rejecting the offers. These regressions include random effects (clustered by seller) on the intercept and offer ratio. The random effects structure was chosen based on the model with the lowest AIC from a model comparison. All variables are z-scored except Number of Best Offer.

	logRT (hours)								
	(counter)	(counter)	(counter)	(counter)	(counter)	(counter)	(counter)	(counter)	(counter)
	(1)	(2)	(3)	(4)	(5)	(6)	(7)	(8)	(9)
Offer/Price (p1/p0)	−0.06	−0.08	−0.01	0.07	0.08	0.07	0.05	0.06	0.05
	(0.19)	(0.19)	(0.20)	(0.09)	(0.09)	(0.09)	(0.08)	(0.08)	(0.08)
Number of Best Offers		−0.16	−0.16		−0.07	−0.06		−0.08	−0.07
		(0.13)	(0.13)		(0.06)	(0.06)		(0.06)	(0.06)
Item Relisted		0.16	0.27		−0.04	−0.06		−0.01	−0.01
		(0.47)	(0.48)		(0.20)	(0.20)		(0.18)	(0.18)
Feedbacks			0.33			−0.10			0.07
			(0.27)			(0.14)			(0.12)
Years since Registration			−0.24			0.10			−0.02
			(0.27)			(0.15)			(0.13)
Viable Items			0.04			0.20			0.17
			(0.28)			(0.12)			(0.11)
Price		0.12	0.07						
		(0.14)	(0.15)						
p1/p0:Feedbacks			−0.07			−0.04			−0.02
			(0.19)			(0.11)			(0.10)
p1/p0:Years			−0.06			0.10			0.06
			(0.21)			(0.10)			(0.09)
p1/p0:Viable Items			0.26			−0.05			−0.03
			(0.20)			(0.07)			(0.06)
Constant	-1.02^{***}	-1.03^{**}	-1.03^{*}	-0.77^{***}	-0.77^{***}	-0.77^{***}	-0.81^{***}	-0.76^{***}	-0.76^{***}
	(0.25)	(0.39)	(0.41)	(0.13)	(0.16)	(0.16)	(0.11)	(0.15)	(0.15)
Observations	171	171	171	771	756	749	942	927	920
Log Likelihood	-333.18	-334.00	-334.66	-1,563.93	-1,536.37	-1,529.92	-1,898.63	-1,870.94	-1,865.88
Akaike Inf. Crit.	674.36	682.01	695.32	3,135.86	3,084.73	3,083.84	3,805.27	3,753.89	3,755.77
Bayesian Inf. Crit.	686.93	704.00	736.16	3,154.45	3,112.50	3,139.27	3,824.66	3,782.88	3,813.66

Note: $^{*}p<0.05$; $^{**}p<0.01$; $^{***}p<0.001$

Table S20. Linear regressions for eBay field experiments 1 and 2 of log(RT) on first buyer offer ratio (p_1/p_0) (z-score) conditional on the seller countering the offers. (1), (2), (3) Experiment 1. (4), (5), (6) Experiment 2. (7), (8), (9) Experiment 1 and 2. Regressions include random effects (clustered by seller) on the intercept. The random effects structure was chosen based on the model with the lowest AIC from a model comparison. All variables are z-scored except Number of Best Offer.

	logRT (hours)		
	(reject)	(reject)	(reject)
	(1)	(2)	(3)
Offer/Price (p1/p0)	0.12	0.14	0.13
	(0.11)	(0.11)	(0.11)
Number of Best Offers		−0.02	−0.02
		(0.04)	(0.04)
Item Relisted		0.26	0.26
		(0.14)	(0.15)
Feedbacks			−0.15
			(0.18)
Years since Registration			−0.06
			(0.17)
Viable Items			0.12
			(0.19)
p1/p0:Feedbacks			−0.11
			(0.13)
p1/p0:Years			−0.09
			(0.12)
p1/p0:Viable Items			0.001
			(0.15)
RT Source Message	0.08	0.09	0.09
	(0.12)	(0.12)	(0.12)
Constant	−0.04	−0.14	−0.14
	(0.16)	(0.17)	(0.18)
Observations	565	560	558
Log Likelihood	-928.15	-922.68	-925.26
Akaike Inf. Crit.	1,870.30	1,863.35	1,880.53
Bayesian Inf. Crit.	1,900.65	1,902.30	1,945.39
Note:	*p<0.05; **p<0.01; ***p<0.001		

Table S21. Linear regressions for eBay field experiment 2 of log(RT) on first buyer offer ratio (p_1/p_0) **(z-score) conditional on the seller rejecting the offers controlling for the source of the response time.** Regressions include random effects (clustered by seller) on the intercept and offer ratio. The random effects structure was chosen based on the model with the lowest AIC from a model comparison. All variables are z-scored except Number of Best Offer.

	logRT (hours)		
	(reject)	(reject)	(reject)
	(1)	(2)	(3)
Offer/Price (p1/p0)	0.45^{***}	0.44^{***}	0.44^{***}
	(0.09)	(0.09)	(0.10)
Number of Best Offers		−0.04	−0.04
		(0.04)	(0.04)
Item Relisted		0.12	0.13
		(0.15)	(0.15)
Feedbacks			−0.04
			(0.14)
Years since Registration			−0.06
			(0.16)
Viable Items			0.09
			(0.16)
p1/p0:Feedbacks			−0.08
			(0.09)
p1/p0:Years			0.02
			(0.10)
p1/p0:Viable Items			−0.04
			(0.10)
Constant	0.79^{***}	0.73^{***}	0.74^{***}
	(0.15)	(0.17)	(0.18)
Observations	864	842	834
Log Likelihood	-1,532.55	-1,491.98	-1,483.57
Akaike Inf. Crit.	3,077.09	2,999.95	2,995.14
Bayesian Inf. Crit.	3,105.66	3,037.84	3,061.31
Note:	$^{*}p<0.05$; $^{**}p<0.01$; $^{***}p<0.001$		

Table S22. Linear regressions for eBay field experiments 1 and 2 of log(RT) on first buyer offer ratio (p_1/p_0) **(z-score) conditional on the seller rejecting the offers or letting the offer expire.** These regressions include random effects (clustered by seller) on the intercept and offer ratio. The random effects structure was chosen based on the model with the lowest AIC from a model comparison. All variables are z-scored except Number of Best Offer.

SI References

1. J. J. Heckman, Sample selection bias as a specification error. *Econometrica* **47**, 153–161 (1979).
2. O. Toomet, A. Henningsen, Sample selection models in R: Package sampleSelection. *J. Stat. Soft.* **27**, 1–23 (2008).
